# Beta-blocker therapy after myocardial infarction: an umbrella review

**DOI:** 10.1016/j.eclinm.2026.103990

**Published:** 2026-05-30

**Authors:** Pilar Cataldo-Miranda, Phuong Khanh Cao, Harvey Jia Wei Koh, Danijela Gasevic, Sri Chodapuneedi, Amminadab L. Eliakundu, Lorena Romero, Dion Stub, Sophia Zoungas, David M. Kaye, Stella Talic

**Affiliations:** a School of Public Health and Preventive Medicine, Monash University, Melbourne, Victoria, Australia; b Centre for Global Health, Usher Institute, The University of Edinburgh, 5-7 Little France Road, Edinburgh, EH16 4UX, UK; c The Ian Potter Library, Alfred Health, Melbourne, Victoria, Australia; d Monash Alfred Baker Centre for Cardiovascular Research, Melbourne, Victoria, Australia; e Victorian Heart Institute, Monash University, Melbourne, Victoria, Australia

**Keywords:** Systematic review, Meta-analysis, Beta-blocker, Myocardial infarction

## Abstract

**Background:**

Beta-blocker therapy remains central to post-myocardial infarction (MI) management, but its benefit in patients with midrange or preserved left ventricular ejection fraction (LVEF) in the reperfusion era remains unclear.

**Methods:**

We conducted an umbrella review and meta-analysis (PROSPERO CRD42024548877). Searches of MEDLINE, EMBASE, and Cochrane Library (January 2019–December 2025) identified systematic reviews and meta-analyses of adults (≥18 years) on beta-blockers for ≥1 year post–MI. Primary outcomes were all-cause mortality, cardiovascular mortality, and recurrent MI. Data from unique primary studies were pooled using random-effects meta-analyses stratified by study design: randomised controlled trials (RCTs), observational studies with propensity score matching (PSM), and non-PSM studies. Meta-regression assessed follow-up duration; subgroup analyses examined midrange (40–49%) and preserved (≥50%) LVEF. Sensitivity analyses were conducted to assess robustness, including the exclusion of studies with converted effect estimates, secondary validation analysis using aggregate estimates from included systematic reviews, and leave-one-out analyses.

**Findings:**

Nineteen systematic reviews, encompassing 52 primary studies (n = 495,827), were included. Data from RCTs (7 studies, n = 24,191), demonstrated a non-significant reduction in all-cause mortality (hazard ratio [HR] 0.89, 95% confidence interval [CI] 0.78–1.01) or cardiovascular mortality (HR 0.94, 95% CI 0.74–1.19). However, a significant risk reduction in recurrence of MI (HR 0.80, 95% CI 0.66–0.97) was observed. Observational studies generally reported larger protective associations for mortality, whereas PSM analysis showed concordant directionality with RCTs for recurrent MI, while non–PSM analysis yielded conflicting results. Meta-regression indicated that in RCTs, the benefit for cardiovascular mortality and recurrent MI attenuated with longer follow-up. In subgroup analyses, patients with preserved LVEF derived no significant mortality benefit from beta-blocker therapy in RCTs, while observational data suggested the greatest risk reductions occurred in those with midrange LVEF. Sensitivity analyses, including leave-one-out analyses, demonstrated stable effect estimates across outcomes, with no single study materially altering the direction of results. Validation analysis highlighted that larger effect sizes in prior meta-analyses were driven by observational data.

**Interpretation:**

Contemporary evidence from RCT confirms that beta-blocker therapy does not significantly reduce mortality but is associated with a reduction in recurrent MI. This benefit appears to attenuate over time and is absent in patients with preserved systolic function. Collectively, these findings challenge the universal, long-term application of historical guideline recommendations and support an individualised, LVEF-stratified approach to beta-blocker therapy.

**Funding:**

None.

**Research in context**

**Evidence before this study**

Beta-blocker therapy has been a cornerstone of post-myocardial infarction (MI) management, particularly for patients with reduced left ventricular ejection fraction (LVEF). Early randomised controlled trials (RCTs) showed reduction in all-cause and cardiovascular mortality, but contemporary MI care, including early reperfusion and advanced acute management, has changed patient outcomes. Over the past two decades, evidence has shown inconsistent effects of beta-blocker therapy on mortality and recurrent MI, with benefits differing across LVEF subgroups.

We systematically searched MEDLINE, EMBASE, and Cochrane Library for systematic reviews and meta-analyses published from January 2019 to December 2025 using the terms (“beta-blocker therapy”) AND (“myocardial infarction”) AND (“meta-analysis”), with no language restrictions. Reviews were eligible if they assessed long-term beta-blocker therapy (≥1 year) versus no use in adults and reported outcomes of all-cause mortality, cardiovascular mortality, or recurrent MI.

Existing syntheses were predominantly based on observational data of moderate-to-high quality but were limited by significant heterogeneity, variable follow-up durations, and inconsistent definitions of LVEF subgroups. While pooled estimates from these reviews suggested mortality benefits, they provided inconsistent evidence regarding the magnitude of benefit across different LVEF subgroups and the durability of effect over time in contemporary reperfusion-era populations.

**Added value of this study**

To our knowledge, this is the most methodologically rigorous contemporary synthesis on this topic. This umbrella review integrates evidence from 19 systematic reviews and 52 unique primary studies (n = 495,827), providing the most comprehensive evaluation of beta-blocker therapy post–MI in the reperfusion era. By directly re-analysing primary study data, we provide a design-stratified analysis that clarifies the highest level of evidence: RCTs show no significant benefit for all-cause or cardiovascular mortality but confirm a reduction in recurrent MI risk. In addition, unlike previous reviews, it incorporates meta-regression analyses and demonstrate attenuation of treatment effects over longer follow-up duration for cardiovascular outcomes. Prespecified subgroup analyses using guideline-based LVEF categories demonstrate no significant mortality benefit for patients with preserved LVEF (≥50%). The study also systematically evaluates evidence quality, quantifies the substantial overlap across prior reviews, potential publication bias, and confirms the robustness of the findings through comprehensive sensitivity analyses, providing a more nuanced and definitive estimate of therapy effectiveness for modern clinical practice.

**Implications of all the available evidence**

Findings indicate that universal, long-term beta-blocker therapy for mortality reduction in all post–MI patients is not supported in contemporary practice. Treatment benefit appears to be modified by LVEF and is most pronounced in the early phase post-event. These findings support a shift in clinical practice towards an individualised, phenotype-stratified approach that considers LVEF, comorbidities, arrhythmic risk, and functional status. This includes recommending periodic reassessment of ongoing treatment need, particularly in patients with preserved LVEF. Clinical guidelines should consider refining recommendations to reflect this nuanced evidence. Future research should prioritise pragmatic randomised trials in reperfusion-era populations to define optimal therapy duration, dosing, and patient selection criteria, thereby supporting updated guidelines that better reflect contemporary cardiovascular care.

## Introduction

Myocardial infarction (MI) remains a leading cause of morbidity and mortality worldwide, with long-term complications persisting despite advances in acute care.^1^ As the global cardiovascular disease burden continues rising,^1^ optimising post–MI management is crucial. Beta-blocker therapy has been a cornerstone of post–MI secondary prevention, based on pre-reperfusion trials,^2^, ^3^, ^4^, ^5^ with clear benefits in patients with reduced left ventricular ejection fraction (LVEF). However, contemporary MI management, marked by early revascularization and improved cardiac care, has decreased the prevalence of significantly reduced LVEF.^6^^,^^7^ Consequently, emerging data from randomised controlled trials (RCTs) and observational studies have questioned the magnitude and efficacy of beta-blocker therapy in patients with midrange or preserved LVEF.^6^

While earlier evidence focused on all-cause mortality,^6^ contemporary decision-making requires clarity on specific cardiovascular endpoints, particularly cardiovascular mortality and recurrent MI. In recent years, multiple systematic reviews and meta-analyses have addressed this question; however, these reviews have reached conflicting conclusions due to substantial methodological heterogeneity, including pooling of randomised and observational designs, inconsistent LVEF definitions, inclusion of populations not representative of contemporary post–MI patients, and variable appraisal of evidence certainty. Consequently, a conventional systematic review and meta-analysis risks reproducing these limitations. We therefore conducted an umbrella review to comprehensively map, appraise, and contextualise the existing evidence base, combined with a primary study–level meta-analysis to generate consistent and unbiased pooled estimates. This dual-layered approach allows both quantification of treatment effects by patient subgroup and study design, and a critical evaluation of the credibility, consistency, and gaps in the contemporary evidence on beta-blocker therapy following MI.

## Methods

This umbrella review and meta-analysis was registered in the PROSPERO database of systematic reviews (CRD42024548877)^8^ and conducted according to the Preferred Reporting Items for Systematic Reviews and Meta-Analyses (PRISMA) guidelines and Preferred Reporting Items for Overviews of Reviews (PRIOR).^9^^,^^10^

### Eligibility criteria for systematic reviews

We included published, peer-reviewed systematic reviews and meta-analyses of RCTs and/or observational studies evaluating adult patients (≥18 years) following MI. Reviews focussing on other cardiovascular events, non-revascularization surgeries, or patients with implanted mechanical cardiac devices were excluded. Only reviews assessing long-term beta-blocker therapy (≥1 year) were eligible. Reviews evaluating short-term use were excluded, and for reviews reporting mixed-duration data, only long-term findings were extracted.

To maintain consistency and interpretability, eligible reviews were required to compare beta-blocker therapy use to no use. Reviews comparing different agents or dosages were excluded. No minimum adherence or persistence thresholds were applied due to inconsistent definitions. Narrative reviews, scoping reviews, conference proceedings, and grey literature were excluded. The primary outcomes of interest were all-cause mortality, cardiovascular mortality and recurrent MI, and these criteria were applied both at the review level and at the level of individual primary studies.

Applying eligibility criteria at the primary study level allowed us to assess alignment across reviews and to facilitate subsequent extraction and re-analysis of primary study data, as described in the Data Extraction section. Primary study eligibility criteria followed the predefined protocol, with the following additional considerations: (1) Outcomes: Primary outcomes remained unchanged; for studies reporting composite outcomes, disaggregated data for individual endpoints were extracted where available. (2) Study design: Randomised controlled trials and observational studies published from 2000 onwards were eligible, reflecting the widespread adoption of reperfusion strategies. (3) Exposure: Definitions of beta-blocker exposure varied across studies and included prescription at hospital discharge, measures of adherence or persistence during follow-up, or treatment status at predefined time points. Accordingly, no minimum adherence or persistence thresholds were applied as inclusion criteria.

### Information sources and search strategy

We systematically searched MEDLINE, EMBASE, and Cochrane Library (Ovid and Wiley) with the assistance of a research librarian (LR) for systematic reviews and meta-analyses published from January 2019 to June 5, 2025 with no language restrictions. To ensure completeness and capture newly published evidence, the search was updated on December 20, 2025. This timeframe was chosen to capture the most recent, methodologically robust syntheses, minimise outdated reviews, and align with current standards of care, while incorporating newer trials and larger datasets for more precise, clinically relevant estimates.

The search strategy was guided by three concepts: “beta-blocker therapy”, “myocardial infarction”, and “meta-analysis”. The complete search strategy can be found in Supplemental Table S1.

### Selection process for reviews

After duplicate removal, two reviewers (PCM, PKC) independently screened all titles and abstracts. Any discrepancies were resolved by discussion, and if consensus was not reached, a third reviewer (LA) was consulted. Full-text articles were retrieved for further assessment, and studies deemed ineligible were documented with reasons for exclusion. The study selection process was recorded in COVIDENCE.^11^

### Data extraction methods

The umbrella review framework was used to systematically identify, appraise, and contextualise the existing body of systematic reviews addressing beta-blocker therapy after MI, thereby characterising sources of heterogeneity, overlap, and methodological limitations across reviews. To avoid propagating biases inherent to review-level pooling, all quantitative syntheses in the present study were based exclusively on unique primary study data extracted from eligible reviews. This hybrid approach preserves the strengths of an umbrella review while ensuring consistent outcome definitions, analytic methods, and avoidance of double-counting at the primary study level.

We extracted data from systematic reviews and their primary studies, but the main analysis relied solely on primary study data to ensure consistent outcome definitions and analytic methods. Two reviewers (PCM, PKC) independently extracted data, with discrepancies resolved through discussion with a third reviewer (SC). Standardised data collection forms, piloted on at least one review and primary study, were used and are available in Supplemental Tables S2 and S3.

### Risk of bias assessment

We assessed the quality of evidence at two levels. First, the methodological quality of included systematic reviews was independently assessed by three reviewers (PCM, PKC, SC) using the Joanna Briggs Institute (JBI) Critical Appraisal Checklist for Systematic Reviews and Research Syntheses.^12^^,^^13^ This tool was selected for its applicability across diverse review designs,^12^^,^^13^ including those incorporating non-randomised studies of interventions, which were common in the included reviews. Each item was scored “Yes” (1 point), “No”, “Unclear,” or “Not Applicable” (0 points), with a maximum score of 11. As the JBI manual does not provide standardised thresholds,^12^ we adopted a pragmatic categorisation scheme based on one recent cardiovascular umbrella review^14^: high quality (≥8/11), moderate quality (6–7/11), and low quality (≤5/11). Discrepancies were resolved through discussion.

Second, as data were extracted and analysed at the primary study level, we also assessed the methodological quality of individual studies. RCTs were evaluated using the Cochrane Risk of Bias 2 (RoB 2) tool^15^ and observational studies using the Risk Of Bias In Non-randomised Studies of Interventions (ROBINS-I) tool,^16^ both recommended for domain-based assessment of internal validity. Risk of bias was reported at the domain and study levels, classified as low, some concerns, or high for RCTs, and low, moderate, serious, or critical for non-randomised studies. Two reviewers independently assessed each study, with disagreements resolved by a third reviewer.

### Methods for synthesising findings

As most primary studies reported hazard ratios (HRs), outcomes were summarised using this measure of association. For studies without HRs but with incidence data, HRs were approximated using event rates and log-transformed confidence intervals (CIs).^17^ When alternative effect measures were reported, HRs and 95% CIs were calculated using established statistical conversion methods described by Fusar-Poli and Rauda.^18^ Studies lacking sufficient data for accurate conversion were excluded from meta-analysis. In cases of post hoc analyses, only original RCT results were included to avoid duplication. For studies with multiple follow-up periods, data from the longest follow-up were included.

### Meta-analysis approach

Random-effects meta-analyses were conducted for each outcome using the DerSimonian-Laird method.^19^ Analyses were stratified by study design: RCTs, observational using propensity score matching (PSM), and observational not using propensity score matching (non-PSM) to account for methodological differences and risk of bias. Forest plots were generated for each outcome by study type. Statistical heterogeneity was assessed using Cochran's Q and I^2^, with I^2^ values interpreted as low (<25%), moderate (25–50%), or high (>50%) per Higgins and Thompson.^20^

### Meta-regression

Univariate meta-regression examined the relationship between follow-up duration (years) and treatment effects for each study design when ≥3 studies were available^21^ (Supplemental Material). Bubble plots visualised effect sizes against follow-up duration, with point sizes proportional to study weights (inverse variance), and regression lines with 95% CIs overlaid.

### Subgroup analysis

As beta-blocker therapy benefits are well established in patients with reduced LVEF, subgroup analyses examined midrange (41–49%) and preserved (≥50%) LVEF categories, per 2022 American Heart Association (AHA) guidelines^22^ (Supplemental Material). Subgroup analyses used random-effects models, with interaction tests assessing statistical significance of subgroup differences.

### Overlap analysis

To avoid double-counting and inflate precision from overlapping primary studies,^23^^,^^24^ we quantified duplication using the Corrected Covered Area (CCA) method. A citation matrix was created with rows representing primary studies and columns representing reviews. Overlap was visualised using a heatmap and interpreted as slight (0–5%), moderate (6–10%), high (11–15%), and very high (>15%).^25^

### Sensitivity analyses

Sensitivity analyses were conducted to assess the robustness of findings. First, weexcluded studies with converted effect measures, which may introduce uncertainty or bias.^26^^,^^27^ To further contextualise our primary study-level meta-analyses, we performed a secondary validation analysis using aggregate effect estimates extracted from included systematic reviews and meta-analyses. These review-level summary estimates were independently analysed and not pooled with the primary data to avoid conflation (Supplemental Material).

Additionally, leave-one-out analyses were conducted by sequentially excluding each study and re-estimating pooled hazard ratios to evaluate the influence of individual studies on overall results. This multi-level strategy provides a comprehensive evaluation of beta-blocker therapy effects while highlighting consistency and discrepancies across evidence types.

### Publication bias

Publication bias was evaluated using complementary approaches. Egger's regression tested funnel plot asymmetry by regressing standardised effect estimates on their precision. Begg's rank correlation test used Kendall's tau to examine the association between standardised effect sizes and variances. Trim-and-fill analyses estimated number of potentially missing studies and provided bias-adjusted effect estimates. Funnel plots of log HRs against standard errors were visually inspected for asymmetry.

### Assessment of certainty of evidence

The certainty of evidence was assessed using the GRADE (Grading of Recommendations, Assessment, Development, and Evaluation) approach.^28^ Two reviewers (PCM, SC) independently classified the certainty of evidence as high, moderate, low, or very low.

### Statistical software and significance

All analyses were performed in R (version 4.5.0) with metafor package (version 4.8.0) for meta-analysis computations. Statistical significance was set at *p* < 0.05 for all tests. Results are reported as HRs with 95% CIs, with I^2^ statistics and τ^2^ values provided to quantify heterogeneity.

### Role of funding source

There was no funding source for this study.

## Results

**Fig. 1:**
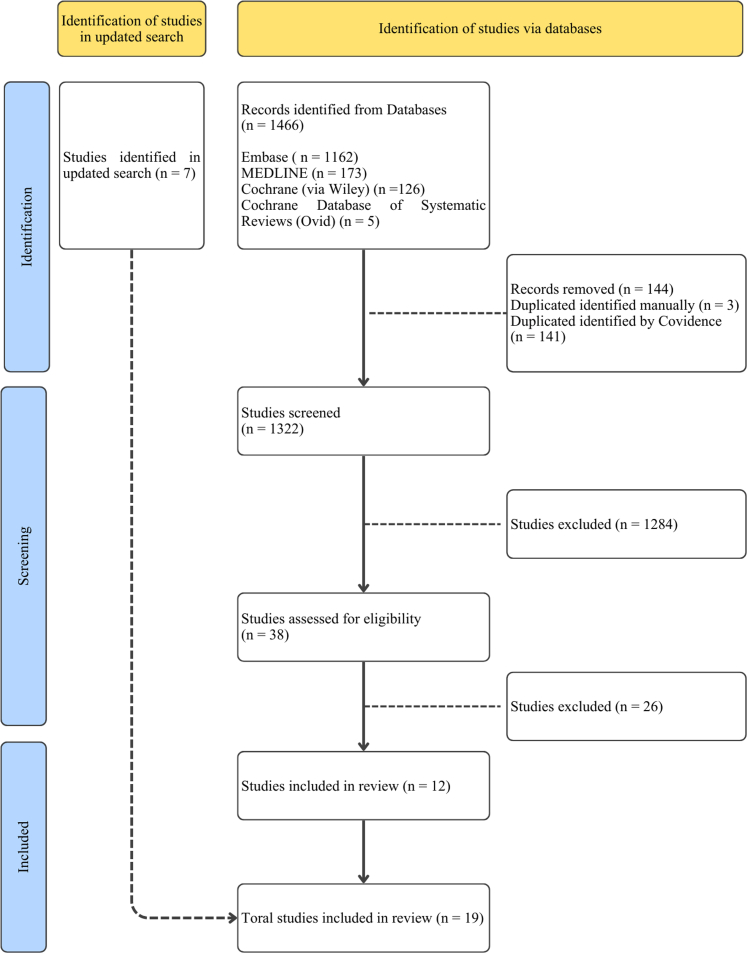
PRISMA Diagram. Caption: PRISMA flow diagram illustrating the study selection process for the umbrella review. The diagram details the number of records identified, screened, assessed for eligibility, and included, along with reasons for exclusions at each stage.

We identified 1466 references; 1320 remained after deduplication. Title and abstract screening excluded 1284 records. Of 38 full texts assessed for eligibility, 26 were excluded (Supplemental Table S4), leaving 12 systematic reviews for inclusion. An updated search conducted in December 2025 identified seven additional eligible systematic reviews and meta-analyses, resulting in a total of 19 systematic reviews included in this umbrella review (Fig. 1). Of these, three focused exclusively on observational studies,^29^, ^30^, ^31^ six on RCTs,^32^, ^33^, ^34^, ^35^, ^36^, ^37^ and ten included both designs.^38^, ^39^, ^40^, ^41^, ^42^, ^43^, ^44^, ^45^, ^46^, ^47^ Published between 2019 and 2025, these reviews encompassed primary studies conducted from 1966 to 2025 across 34 countries, predominantly in Europe and Asia (Table 1).

**Table 1:** Characteristics of included systematic reviews.

Systematic Review (Year)	Number (type) of included studies	Date range of included studies	Population	Study participants: age, sex, LVEF, type of MI	Total sample size	Intervention definition (sample size)	Control definition (sample size)	Outcomes assessed	Follow-up	JBI quality score	GRADE
Affas et al. (2025)^44^	6	2014–2024	STEMI patients with preserved LVEF (≥40%) after PCI	Mean age 63.5 Males range 75–83% Mean LVEF not reported Type of MI: STEMI	28,736	Beta-blocker users, any type of agent/dose	No beta-blocker use	All-cause mortality Recurrent MI Cardiovascular mortality Hospitalisation for heart failure Stroke	Mean not reported	7/11	All-cause mortality: Very Low Cardiovascular mortality: Very Low Recurrent MI: Low
	(4 observational and 2 RCTs)					(13,650)	(15,086)		(Range 1–4.5 years)		
Alnemer et al. (2025)^45^	16	2004–2024	Patients with MI and preserved LVEF (≥40%)	Age range 43.6–72.1 Males range 21.5–83% LVEF range 49–59.4% Type of MI: STEMI and NSTEMI	78,555	Beta-blocker users, any type of agent/dose	No beta-blocker use	All-cause mortality Cardiovascular mortality MACE Recurrent MI Rehospitalization	Mean not reported	7/11	All-cause mortality: Low Cardiovascular mortality: Low Recurrent MI: Moderate
	(13 observational and 3 RCTs)					(57,901)	(20,654)		(Range 0.5–5.2 years)		
Chen et al. (2025)^47^	18	2014–2024	Patients with MI and preserved or mildly reduced LVEF (≥40%)	Age range 58–68.6 Males range 68.1–83% Mean LVEF not reported Type of MI: STEMI and NSTEMI	66,282	Beta-blocker users, any type of agent/dose	No beta-blocker use	All-cause mortality Cardiovascular mortality MACE Recurrent MI Hospitalisation for heart failure Stroke	Mean not reported	9/11	All-cause mortality: Very Low Cardiovascular mortality: Very Low Recurrent MI: -
	(16 observational and 2 RCTs)					(46,546)	(19,736)		(Range 0.5–5 years)		
Chi et al. (2024)^39^	22	2010–2024	Adult patients aged over 18 experiencing MI with no reduced LVEF (<40%) or no history of heart failure	Mean age not reported Sex proportion not reported Mean LVEF not reported Type of MI not reported	285,685	Beta-blocker users, any type of agent	No beta-blocker use	All-cause mortality	Mean not reported	11/11	All-cause mortality: Low Cardiovascular mortality: - Recurrent MI: -
	(19 observational and 3RCTs)					(239,287)	(46,398)		(Range 1–12 years)		
DahlAarvick et al. (2019)^29^	16	2004–2017	Patients post-AMI in which none or only a minority of the population had a history of heart failure, or Killip Class <3, or LVEF >40% at baseline	Median age 64.6 (IQR 57.7–68.6) 75% males Median LVEF 53.7%^a^ Type of MI not reported	189,385	Beta-blocker users, any type of agent/dose	No beta-blocker use	All-cause mortality	Median 2.7 years	9/11	All-cause mortality: Low Cardiovascular mortality: - Recurrent MI: -
	(All observational)					(189,385)	(24,977)		(Range 0.5–5.2 years)		
Gomes et al. (2025)^40^	11	2010–2024	Patients post–MI with preserved ejection fraction	Mean age not reported 71.8% males Mean LVEF not reported Type of MI not reported	85,607	Beta-blocker users, any type of agent/dose	Placebo or absence of BBs	All-cause mortality Cardiovascular mortality Recurrent MI Stroke MACE Heart failure	Mean 3 years	4/11	All-cause mortality: Low Cardiovascular mortality: Low Recurrent MI: Low
	(9 observational and 2 RCTs)					(65,790)	(19,817)		range not reported		
(1) Hu et al. (2022)^42^	9	2013–2021	Patients with acute MI in the contemporary reperfusion era.	Mean age not reported Sex proportion not reported Mean LVEF not reported Type of MI: STEMI and NSTEMI	47,339	Use of beta-blockers after hospital discharge, any type of agent/dose	Non-use of beta-blockers after hospital discharge	All-cause mortality Cardiovascular mortality Recurrent MI Stroke MACE Revascularization Heart failure	Mean not reported	7/11	All-cause mortality: Moderate Cardiovascular mortality: Moderate Recurrent MI: moderate
	(8 observational and 1 RCT)					(24,329)	(23,010)		(Range 1–4 years)		
(2) Hu et al. (2022)^41^	15	2010–2022	Patients diagnosed with acute coronary syndrome without heart failure or left ventricular systolic dysfunction	Mean age not reported Males range 69–83.9% Mean LVEF not reported Type of MI: STEMI and NSTEMI	205,672	Beta-blocker users, any type of agent/dose	No beta-blocker use	All-cause mortality Cardiovascular mortality Recurrent MI Stroke MACE Revascularization Heart failure	Mean not reported	9/11	All-cause mortality: Moderate Cardiovascular mortality: Low Recurrent MI: Moderate
	(14 observational and 1 RCT)					(186,583)	(19,089)		(Range 1–5 years)		
Kim et al. (2022)^31^	5	2016–2021	Patients who had had MI and no heart failure at baseline.	Mean age not reported Sex proportion not reported Mean LVEF not reported Type of MI not reported	217,532	Beta-blocker prescription continued for 1 year after hospital discharge	No beta-blocker prescription for 1 year after hospital discharge	All-cause mortality	Mean not reported	7/11	All-cause mortality: Very Low Cardiovascular mortality: - Recurrent MI: -
	(All observational)					(196,631)	(20,901)		(Range 1–3 years)		
Kristensen et al. (2025)^35^	5	2018–2025	Patients with MI and a preserved LVEF (≥50%).	Median age 62 (IQR 55 to 71) 79.3% males Mean LVEF not reported Type of MI: STEMI and NSTEMI	17,801	Beta-blocker users, any type of agent/dose	No beta-blocker use	Composite outcome of all-cause mortality, recurrent MI and heart failure All-cause mortality Recurrent MI Heart failure Cardiovascular mortality Unplanned coronary revascularization Malignant ventricular arrhythmias	Median 3.6 years	7/11	All-cause mortality: Low Cardiovascular mortality: Low Recurrent MI: Moderate
	(All RCTs)					(8831)	(8970)		(IQR 2.3–4.6)		
Liang et al. (2022)^30^	29	1998–2020	Patients after MI where none or only a minority of patients had LVEF <40% at baseline.	Mean age not reported Sex proportion not reported Mean LVEF not reported Type of MI not reported	242,013	Beta-blocker users, any type of agent/dose	No beta-blocker use	All-cause mortality Cardiovascular mortality Recurrent MI Stroke MACE Revascularization Heart failure	Mean not reported	9/11	All-cause mortality: Moderate Cardiovascular mortality: Moderate Recurrent MI: Low
	(All observational)					(195,995)	(46,018)		(Range 0.5–5.2 years)		
Maqsood et al. (2021)^43^	12	2004–2019	STEMI patients with preserved LVEF (≥40%) after PCI	Mean age 64 years (± 3 years) 75% males Mean LVEF not reported Type of MI: STEMI	32,108	Beta-blocker users, any type of agent/dose	No beta-blocker use	All-cause mortality MACE	Mean not reported	9/11	All-cause mortality: Moderate Cardiovascular mortality: - Recurrent MI: -
	(11 observational and 1 RCT)					(19,740)	(12,368)		(Range 0.5–4.7 years)		
Rossello et al. (2025)^36^	4	2018–2025	Patients with MI and mildly reduced LVEF (40–49%) without heart failure.	Median age 63 (IQR 55–71) 81% males Median LVEF 45% (IQR 45–47.5%) Type of MI: STEMI and NSTEMI	1885	Beta-blocker users, any type of agent/dose	No beta-blocker use	Composite outcome of all-cause mortality, recurrent MI and heart failure All-cause mortality Recurrent MI Heart failure Cardiovascular mortality Unplanned coronary revascularization Malignant ventricular arrhythmias	Median 3.5 years	7/11	All-cause mortality: Moderate Cardiovascular mortality: Moderate Recurrent MI: Moderate
	(All RCTs)					(991)	(894)		(IQR 2.3–4.5)		
Sabina et al. (2025)^34^	3	2018–2024	Patients with MI and a preserved LVEF (≥40%).	Mean age not reported 80% males Mean LVEF not reported Type of MI not reported	9512	Treated with beta-blockers, any agent/dose	Not treated with beta-blockers	All-cause mortality Cardiovascular mortality Recurrent MI Stroke	Mean not reported	9/11	All-cause mortality: Low Cardiovascular mortality: Low Recurrent MI: Low
	(All RCTs)					(4748)	(4764)		(Range 3–4 years)		
Safi et al. (2019)^33^	63	1966–2018	Any patient (irrespective of age and sex) with suspected or diagnosed acute MI.	Mean age 57.4 years 74.5% males Mean LVEF not reported Type of MI: STEMI and NSTEMI	85,550	Use of any type of beta-blocker (including intravenous and oral therapy)	Placebo, no intervention or co-intervention	All-cause mortality Cardiovascular mortality Recurrent MI MACE Angina	Median 17.7 months at maximum follow-up	11/11	All-cause mortality: Moderate Cardiovascular mortality: Moderate Recurrent MI: Low
	(All RCTs)					(not reported)	(not reported)		(Range 0.5–5 years)		
Safi et al. (2021)^32^	21	1974–2018	Patients of any age diagnosed with MI in the non-acute, stable phase following an acute MI. Includes those with Killip class I/II or NYHA class I/II heart failure, or acute heart failure during hospitalisation as defined by trialists.	Mean age 56.9 years 82.7% males Mean LVEF not reported Type of MI not reported	22,085	Beta-blockers initiated during the non-acute phase after acute MI.	Placebo, no intervention or co-intervention	All-cause mortality Cardiovascular mortality Recurrent MI MACE Angina	Median 43.3 months	11/11	All-cause mortality: Low Cardiovascular mortality: Very Low Recurrent MI: Moderate
	(All RCTs)					(11,236)	(10,849)		(Range 0.8–5 years)		
Sidiq et al. (2025)^37^	4	2018–2025	Patients with MI and preserved or mildly reduced LVEF (≥40%)	Age range 61.3–65 Males range 77.5–81% Mean LVEF not reported Type of MI: STEMI and NSTEMI	19,826	Beta-blocker users, any type of agent/dose	No beta-blocker use	All-cause mortality Recurrent MI Heart failure	Median 3.65 years	6/11	All-cause mortality: Moderate Cardiovascular mortality: - Recurrent MI: Moderate
	(All RCTs)					(9892)	(9934)		(IQR 2.3–4.6)		
Singh et al. (2024)^38^	8	2004–2024	Adult patients with acute coronary syndrome and preserved LVEF (≥50%)	Mean age 63 years 75% males Mean LVEF not reported Type of MI not reported	23,697	Beta-blocker users, any type of agent/dose	No beta-blocker use	All-cause mortality Cardiovascular mortality Recurrent MI Hospitalisation for heart failure	Mean 3 years	9/11	All-cause mortality: Low Cardiovascular mortality: Very Low Recurrent MI: Low
	(6 observational and 2 RCTs)					(17,061)	(6636)		(Range 0.5–5.2 years)		
Yang et al. (2025)^46^	34	2000–2025	Adult patients following MI	Mean age not reported Sex proportion not reported Mean LVEF not reported Type of MI: STEMI and NSTEMI	233,303	Beta-blocker users, any type of agent/dose	No beta-blocker use	All-cause mortality Cardiovascular mortality MACE	Mean not reported	5/11	All-cause mortality: Low Cardiovascular mortality: Low Recurrent MI: -
	(30 observational and 4 RCTs)					(not reported)	(not reported)		(Range 0.5–5.2 years)		

RCT = Randomised Controlled Trial; MI = Myocardial Infarction; LVEF = Left Ventricular Ejection Fraction; JBI = Joanna Brigg's Institute; SD = Standard Deviation; IQR = Interquartile Range STEMI = ST- Elevation Myocardial Infarction; NSTEMI = Non-ST- Elevation Myocardial Infarction; MACE = Major Adverse Cardiac Event.

a Mean based on 10 studies.

Where reported, mean participant age ranged from 56.9^32^ to 64.6^29^ years, though eight reviews did not report age.^30^^,^^31^^,^^34^^,^^39^, ^40^, ^41^, ^42^^,^^46^ Male participants comprised 71.8%^40^ to 82.7%^32^ of samples; five reviews did not report sex distribution.^30^^,^^31^^,^^39^^,^^41^^,^^46^ Nine reviews included both ST-segment elevation MI (STEMI) and non-ST-segment elevation MI (NSTEMI) events^33^^,^^35^, ^36^, ^37^^,^^41^^,^^42^^,^^45^, ^46^, ^47^; two included only STEMI,^43^^,^^44^ and eight did not specify type of MI.^29^, ^30^, ^31^, ^32^^,^^34^^,^^38^, ^39^, ^40^ Regarding LVEF, 12 reviews specified an inclusion threshold. Nine reviews included patients with preserved or mildly reduced LVEF, defined as ≥40%.^29^^,^^30^^,^^34^^,^^37^^,^^39^^,^^43^, ^44^, ^45^^,^^47^ Two reviews included only patients with preserved LVEF, defined as ≥50%.^35^^,^^38^ One review included only patients with mildly reduced LVEF (40–49%).^36^ One review did not specify the threshold used to define preserved LVEF,^40^ three reviews did not use LVEF as inclusion criteria but excluded participants with heart failure,^31^^,^^32^^,^^41^ and three reviews made no distinctions in this regard.^33^^,^^42^^,^^46^

Of the 73 primary studies published from 2000 onward that were screened, 52 met the inclusion criteria (Table 2). The excluded primary studies are listed in Supplemental Table S5. Among included studies, six were RCTs,^50^^,^^52^^,^^54^, ^55^, ^56^, ^57^ and four were post hoc analyses of trials^48^^,^^49^^,^^51^^,^^53^; seven were observational studies reporting only PSM results,^82^, ^83^, ^84^, ^85^, ^86^, ^87^, ^88^ 24 were observational studies reporting only non-PSM results,^58^, ^59^, ^60^, ^61^, ^62^, ^63^, ^64^, ^65^, ^66^, ^67^, ^68^, ^69^, ^70^, ^71^, ^72^, ^73^, ^74^, ^75^, ^76^, ^77^, ^78^, ^79^, ^80^, ^81^ and 11 were observational studies reporting both PSM and non-PSM results.^89^, ^90^, ^91^, ^92^, ^93^, ^94^, ^95^, ^96^, ^97^, ^98^, ^99^

**Table 2:** Characteristics of included primary studies.

Author (year)	Type of study	Name of study/registry	Population	Study participants: Age (in years), gender, mean LVEF, type of MI	Total sample size	Intervention definition (sample size)	Control definition (sample size)	Outcomes assessed	Follow-up
RCTs									
Amano et al. (2023)^48^	Post-hoc analysis of CAPITAL-RCT	CAPITAL-RCT	STEMI patients with preserved LV function (≥40%) who underwent primary PCI	Mean age: 63.9 Intervention; 64.5 Control Males: 83% Intervention; 78% Control Mean LVEF: 58.1% Intervention; 58.3% Control Type of MI: All STEMI	794	Carvedilol within 7 days after primary PCI (394)	No beta-blocker therapy (400)	Primary outcome: composite of all-cause death, myocardial infarction, hospitalisation for acute coronary syndrome, and hospitalisation for heart failure. Secondary outcomes: composite of cardiac death, MI, and hospitalisation for heart failure, and the individual components of the primary outcome.	Median 3.9 years (IQR 3–4.6)
Bangalore et al. (2014)^49^	Post-hoc analysis of CHARISMA Trial	CHARISMA Trial (Clopidogrel for High Atherothrombotic Risk and Ischaemic Stabilisation, Management, and Avoidance)	**This review only included a subpopulation:** Patients age 45 years or older with prior MI.	Mean age: 64.5 Intervention; 64.6 Control Males: 79.4% Intervention; 79.3% Control Mean LVEF: Not reported Type of MI: Not reported	4772	Use of beta-blocker therapy (3451)	No beta-blocker therapy (1321)	Primary outcome: composite of first occurrence of nonfatal MI, stroke, or death from cardiovascular causes. Secondary outcomes: individual components of the primary outcome plus death from any cause, and hospitalisation for cardiovascular cause (unstable angina, a transient ischaemic attack, or a revascularization procedure).	Median 28 months
Dargie et al. (2001)^50^	Multi-centre double-blind Randomised controlled trial	CAPRICORN (Carvedilol Post-Infarct Survival Control in LV Dysfunction)	Post-AMI patients with left ventricular dysfunction (LVEF ≤40%) with or without heart failure	Mean age: 63 Intervention and Control Males: 73% Intervention; 74% Control Mean LVEF: 32.9% Intervention; 32.7% Control Type of MI: Not reported	1959	Carvedilol (975)	Placebo (984)	Co-primary outcomes: all-cause mortality and all-cause mortality or cardiovascular hospital admissions. Secondary outcomes: sudden death and hospital admission for heart failure. Other outcomes assessed: recurrent non-fatal myocardial infarction, and all-cause mortality or recurrent non-fatal myocardial infarction.	Mean 1.3 years
Hioki et al. (2016)^51^	Post-hoc analysis ALPS AMI study	ALPS - AMI (Assessment of Lipophilic vs. hydroPhilic Statin therapy in Acute Myocardial Infarction)	Post–MI Killip class 1 patients who underwent primary PCI.	Mean age: 65.2 Intervention; 66.3 Control Males: 80.1% Intervention; 83.9% Control Mean LVEF: 56% Intervention; 56.2% Control Type of MI: 82.1% STEMI Intervention; 81.3% STEMI Control	444	Use of beta-blocker therapy (251)	No beta-blocker therapy (193)	Primary outcome: All-cause mortality	Mean 1040 days (SD ± 186)
Ibanez et al. (2025)^52^	Multicountry, multicenter, open-label, single-blind randomised controlled trial	REBOOT Trial (Treatment with Beta-Blockers after Myocardial Infarction without Reduced Ejection Fraction)	Post–MI patients with preserved or mildly reduced LVEF (>40%) who underwent coronary angiography.	Mean age: 61.4 Intervention; 61.3 Control Males: 80.6% Intervention; 80.8% Control Mean LVEF: 57% Intervention; 57.2% Control Type of MI: 51% STEMI Intervention; 50.8% STEMI Control	8438	Use of beta-blocker therapy (4207)	No beta-blocker therapy (4231)	Primary outcome: composite of death from any cause, reinfarction, or hospitalisation for heart failure. Secondary outcomes: individual components of the primary outcome, death from cardiac causes, sustained ventricular tachycardia, ventricular fibrillation, and resuscitated cardiac arrest. Tertiary outcomes: unplanned revascularization and a composite of death from cardiac causes, stroke, or myocardial infarction	Median 3.7 years
McMurray et al. (2005)^53^	Post hoc analysis CAPRICORN Trial	CAPRICORN (Carvedilol Post-Infarct Survival Control in LV Dysfunction)	Post-AMI patients with left ventricular dysfunction (LVEF ≤40%) with or without heart failure	Mean age: 63 Intervention and Control Males: 73% Intervention; 74% Control Mean LVEF: 32.9% Intervention; 32.7% Control Type of MI: Not reported	1959	Carvedilol (975)	Placebo (984)	Primary outcomes: Atrial and ventricular arrhythmias	Mean 1.3 years
Munkhaugen et al. (2025)^54^	Multicountry, multicenter, open-label, single-blind randomised controlled trial	BETAMI–DANBLOCK trial (Norwegian Beta-Blocker Treatment after Acute Myocardial Infarction in Revascularized Patients without Reduced Left Ventricular Ejection Fraction) and (Danish Trial of Beta-Blocker Therapy after Myocardial Infarction without Heart Failure)	Post–MI patients without heart failure and with preserved or mildly reduced left ventricular ejection fraction (LVEF ≥40% in BETAMI; LVEF >40% in DANBLOCK).	Median age: 68 Intervention; 62 Control Males: 78.4% Intervention; 79.9% Control Mean LVEF: Not reported Type of MI: 47.8% STEMI Intervention; 47.2% STEMI Control	5574	Use of beta-blocker therapy (2783)	No beta-blocker therapy (2791)	Primary outcome: composite of death from any cause or major adverse cardiovascular events (new myocardial infarction, unplanned coronary revascularization, ischaemic stroke, heart failure, or malignant ventricular arrhythmias). Secondary outcomes: each component of the primary end point and hospitalisation for pacemaker implantation or second- or third-degree atrioventricular block.	Median 3.5 years (IQR 2.2–4.6)
Silvain et al. (2024)^55^	Multicenter, open-label, non-inferiority Randomised controlled trial	ABYSS Trial (The Assessment of β-blocker interruption 1 Year after an uncomplicated myocardial infarction on Safety and Symptomatic cardiac events requiring hospitalisation)	Post–MI patients with preserved left ventricular ejection fraction (LVEF ≥40%)	Mean age: 63.5 Intervention; 63.5 Control Males: 82.9% Intervention; 82.7% Control Mean LVEF: 60% Intervention; 60% Control Type of MI: 63.3% STEMI Intervention; 62.7% STEMI Control	3698	Interruption of beta-blocker therapy (1846)	Continuation of beta-blocker therapy (1852)	Primary outcome: composite of death, nonfatal myocardial infarction, nonfatal stroke, or hospitalisation for cardiovascular reasons. Secondary outcomes: change in score from baseline to 6 months and 12 months on the European Quality of Life–5 Dimensions (EQ-5D) questionnaire, composite of death, nonfatal myocardial infarction, or nonfatal stroke and a composite of death, nonfatal myocardial infarction, nonfatal stroke, or hospitalisation for heart failure.	Median 3 years (IQR 2–4)
Watanabe et al. (2018)^56^	Multi-centre open-label Randomised controlled trial	CAPITAL-RCT (Carvedilol Post-Intervention Long-Term Administration in Large-scale Randomised Controlled Trial)	STEMI patients with preserved LV function (≥40%) who underwent primary PCI	Mean age: 63.9 Intervention; 64.5 Control Males: 83% Intervention; 78% Control Mean LVEF: 58.1% Intervention; 58.3% Control Type of MI: All STEMI	794	Carvedilol within 7 days after primary PCI (394)	No beta-blocker therapy (400)	Primary outcome: composite of all-cause death, myocardial infarction, hospitalisation for acute coronary syndrome, and hospitalisation for heart failure. Secondary outcomes: individual components of the primary endpoint plus cardiac death, non-cardiac death, stroke, vasospastic angina, major bleeding, definite stent thrombosis, target-lesion revascularization, and any coronary revascularization.	Median 3.9 years (IQR 3–4.6)
Yndigegn et al. (2024)^57^	Multi-centre, registry-based, open-label, parallel-group Randomised controlled trial	REDUCE-AMI (Randomised Evaluation of Decreased Usage of Beta-Blockers after Acute Myocardial Infarction)	Post-AMI patients with preserved left ventricular ejection fraction (≥50%) who had undergone coronary angiography	Mean age: 65 Intervention and Control Males: 77.6% Intervention; 77.4% Control Mean LVEF: Not reported Type of MI: 35% STEMI Intervention; 35.5% STEMI Control	5020	Long-term treatment Metoprolol or Bisoprolol (2508)	No beta-blocker therapy (2512)	Primary outcome: composite of death from any cause or new myocardial infarction. Secondary outcomes: death from any cause, death from cardiovascular causes, myocardial infarction, hospitalisation for atrial fibrillation, and hospitalisation for heart failure.	Median 3.5 years (IQR 2.2–4.7)
Without PSM									
Andell et al. (2015)^58^	Nationwide retrospective cohort study	SWEDEHEART (Swedish Web-system for Enhancement and Development of Evidence-based care in Heart disease Evaluated According to Recommended Therapies)	Post–MI patients with concurrent COPD diagnosis	Mean age: 74 Intervention; 77 Control Males: 56.3% Intervention; 49.5% Control Mean LVEF: Not reported Type of MI: 25.4% STEMI Intervention; 17.1% STEMI Control	4858	Beta-blocker therapy prescribed at discharge (4086)	No beta-blocker therapy (772)	Primary outcome: All-cause mortality	Median 1033 days (IQR 1141)
Arós et al. (2006)^59^	Multicenter prospective cohort study	PRIAMHO II (Proyecto de Registro de Infarto Agudo de Miocardio HOspitalario)	**This review only included a subpopulation:** post–MI patients who either did not received medication or received only beta-blocker therapy	Mean age: 60.6 Intervention; 65.5 Control Males: 80.6% Intervention; 78% Control Mean LVEF: Not reported Type of MI: 66.4% STEMI Intervention; 65.3% STEMI Control	3029	Beta-blocker therapy only prescribed at discharge (1833)	No medications prescribed at discharge (1196)	Primary outcome: All-cause mortality	1 year (endpoint)
Bao et al. (2013)^60^	Multicenter retrospective cohort study	CREDO - Kyoto Registry (Coronary REvascularization Demonstrating Outcome Study in Kyoto)	Patients who underwent PCI within 24 h from onset of STEMI and survived the index hospitalisation	Mean age: 65.8 intervention; 68 Control Males: 77.8% Intervention; 72.2% Control Mean LVEF: 52.4% Intervention; 54.3% Control Type of MI: All STEMI	3692	Beta-blocker therapy prescribed at discharge (1614)	No beta-blocker therapy (2078)	Primary outcome: composite of cardiovascular mortality and recurrent MI Secondary outcomes: all-cause mortality, cardiovascular mortality, recurrent MI and hospitalisation for heart failure	Median 955 days (IQR 693–1248)
Chen et al. (2020)^61^	Single-centre retrospective cohort study	–	**This review only included a subpopulation**: post–MI patients who underwent PCI with adequate systolic function (LVEF ≥40%)	Mean age: 58 Intervention; 56 Control Males: 84.5% Intervention; 86.2% Control Mean LVEF: 60% Intervention and Control Type of MI: Not reported	1560	Beta-blocker therapy prescribed at discharge (1444)	No beta-blocker therapy (116)	Primary outcome: All-cause mortality Secondary outcomes: cardiovascular mortality, recurrent MI and MACCE (composite of all-cause mortality, nonfatal MI, unplanned target vessel revascularization, stent thrombosis, and stroke)	2 years (endpoint)
De Luca et al. (2005)^62^	Prospective cohort study	–	STEMI patients treated with primary angioplasty	Mean age: 60 Intervention; 61 Control Males: 77.8% Intervention; 77.7% Control Mean LVEF: 43% Intervention and Control Type of MI: All STEMI	1513	Beta-blocker therapy prescribed at discharge (1325)	No beta-blocker therapy (188)	Primary outcome: all-cause mortality	1 year (endpoint)
Dondo et al. (2017)^63^	Multicenter retrospective cohort study	MINAP (Myocardial Ischaemia National Audit Project)	Post–MI patients without heart failure or systolic dysfunction (LVEF <30%)	Mean age: 63.3 Intervention; 68.6 Control Males: 71.4% Intervention; 61.7% Control Mean LVEF: Not reported Type of MI: 53.7% STEMI Intervention; 35.2% STEMI Control	179,810	Beta-blocker therapy prescribed at discharge (170,475)	No beta-blocker therapy (9335)	Primary outcome: All-cause mortality	1 year (endpoint)
El Nasasra et al. (2020)^64^	Multicenter retrospective cohort study	ACSIS (Acute Coronary Syndrome Israeli Surveys)	Patients with acute coronary syndrome without reduced systolic function (LVEF ≥40%) and/or clinical signs of heart failure	Mean age: 60.8 Intervention; 62.2 Control Males: 79.2% Intervention; 78.3% Control Mean LVEF: Not reported Type of MI: Not reported	7392	Beta-blocker therapy prescribed at discharge (6007)	No beta-blocker therapy (1385)	Primary outcome: 30-day MACE (composite of 30-day all-cause mortality, recurrent MI, recurrent ischaemia, stent thrombosis, ischaemic stroke and urgent revascularization) Secondary outcomes: all-cause mortality	1 year (endpoint)
Holt et al. (2021)^65^	Nationwide retrospective cohort study	–	Patients aged 30–85 years with a first-time admission of MI, who underwent PCI or coronary angiography, were optimally treated (redeemed a prescription of acetyl-salicylic acid and statins) and had no heart failure	Mean age: 61 Intervention; 62 Control Males: 74.8% Intervention; 68.1% Control Mean LVEF: Not reported Type of MI: Not reported	30,177	Beta-blocker therapy prescription redeemed within the first 30 days following discharge (24,770)	No beta-blocker therapy during a 90-day period (5407)	Primary outcome: cardiovascular mortality, recurrent MI, composite outcome of cardiovascular mortality, recurrent MI, first time diagnosis of heart failure, stroke, angina, PCI, CAG or CABG. Secondary outcomes: composite outcome of adverse events related to beta-blocker therapy (claudication, hypoglycaemic episodes, pace-maker implantation, conduction blocks, hypotension and syncope)	33 months (endpoint)
Hwang et al. (2019)^66^	Nationwide retrospective cohort study	KAMIR-NIH (Korea Acute Myocardial Infarction Registry – NIH)	Post–MI patients	Mean age: 62.5 Intervention; 65.6 Control Males: 75.1% Intervention; 72.9% Control Mean LVEF: 52.35% Intervention; 52.5% Control Type of MI: 44.1% STEMI Intervention; 38.1% STEMI Control	11,909	Use of beta-blocker therapy up to one year after discharge maintaining type and dose (9894)	No beta-blocker therapy (2015)	Primary outcome: Cardiovascular mortality	1 year (endpoint)
Ishak et al. (2023)^67^	Nationwide retrospective cohort study	SWEDEHEART Registry	Post–MI patients without systolic dysfunction and heart failure (LVEF ≥50%)	Mean age: 64 Intervention; 65 Control Males: 74.9% Intervention; 72.9% Control Mean LVEF: Not reported Type of MI: 37.8% STEMI Intervention; 30.5% STEMI Control	43,618	Use of beta-blocker therapy use at 1 year (34,253)	No use of beta-blocker therapy at 1 year (9365)	Primary outcome: composite of all-cause mortality, recurrent MI, unscheduled revascularisation, and hospitalisation for heart failure. Secondary outcomes: individual components of primary outcome plus cardiovascular mortality and stroke.	Median 4.5 years (IQR not reported)
Ishikawa et al. (2000)^68^	Single-centre retrospective cohort study	–	Post–MI patients	Mean age: 59.1 Intervention; 61.5 Control Males: 78.6% Intervention; 78.3% Control Mean LVEF: Not reported Type of MI: Not reported	1483	Beta-blocker therapy prescribed at discharge (833)	No beta-blocker therapy (650)	Primary outcome: Cardiac events (composite of recurrent MI, sudden death, and death by congestive heart failure) Secondary outcomes: all-cause mortality, cardiovascular mortality	Mean 17.4 months (SD ± 20.9 months)
Jackevicius et al. (2020)^69^	Population-based retrospective cohort study	Canadian Institute for Health Information (CIHI) Discharge Abstract Database	Stable patients aged ≥65 years with previous MI within the past 3 years	Mean age: 77.8 Intervention and Control Males: 55.8% Intervention and Control Mean LVEF: Not reported Type of MI: 25.3% STEMI Intervention; 23.3% STEMI Control	33,811	Dispensation of beta-blocker therapy prescription within 100 days before index date (21,440)	No beta-blocker therapy dispensed within 100 days before index date (12,371)	Primary outcome: composite of all-cause mortality, hospitalisation for MI or angina. Secondary outcomes: all-cause mortality and composite of all-cause mortality or hospitalisation for MI	3 years (endpoint)
Lee et al. (2015)^70^	Single-centre retrospective cohort study	–	STEMI patients who underwent primary PCI within 24 h	Mean age: 56 Intervention; 61 Control Males: 82.1% Intervention; 74.3% Control Mean LVEF: 53% Intervention; 49% Control Type of MI: All STEMI	901	Use of beta-blocker therapy at hospital discharge (598)	No beta-blocker therapy (303)	Primary outcome: All-cause mortality Secondary outcomes: MACE (composite of cardiovascular mortality, recurrent MI, and target vessel revascularization)	Mean 54 months (SD ± 30)
Lee et al..(2016)^71^	Nationwide retrospective cohort study	–	**This review only included a subpopulation**: post–MI patients who underwent PCI and belonged to either “no drugs” and “BB only” groups	Mean age: 61.9 Intervention, 63 Control Males: 75.4% Intervention; 74.8% Control Mean LVEF: Not reported Type of MI: Not reported	7261	Beta-blocker therapy only prescribed at discharge (3683)	No medications prescribed at discharge (3578)	Primary outcome: All-cause mortality	Median 2.4 years (IQR 1.4–3.4)
Okuno et al. (2019)^72^	Nationwide retrospective cohort study	J-MINUET study (Japanese Registry of Acute Myocardial Infarction Diagnosed by Universal Definition)	Post–MI patients	Variables were reported by heart rate group, not beta-blocker therapy use	2799	Beta-blocker therapy prescribed at discharge (1919)	No beta-blocker therapy (880)	Primary outcome: MACE (composite of all-cause death, non-fatal MI, non-fatal stroke, heart failure, and urgent revascularization for unstable angina). Secondary outcomes: death; composite of death and non-fatal MI; composite of death, non-fatal MI, and non-fatal stroke; and composite of death, non-fatal MI, non-fatal stroke, and heart failure.	3 years (endpoint)
Ozasa et al. (2010)^73^	Multicenter retrospective cohort study	j-Cypher Registry	STEMI patients who underwent primary PCI within 24 h	Mean age: 66.4 Intervention; 68 Control Males: 75% Intervention; 76% Control Mean LVEF: 51% Intervention; 53.2% Control Type of MI: All STEMI	910	Beta-blocker therapy prescribed at discharge (349)	No beta-blocker therapy (561)	Primary outcome: All-cause mortality Secondary outcomes: MACE (composite of all-cause mortality, recurrent MI and heart failure hospitalisation)	3 years (endpoint)
Park et al. (2019)^74^	Single centre retrospective cohort study		Post–MI patients without systolic dysfunction and heart failure (LVEF ≥40%)	Mean age: 61 Intervention; 63.8 Control Males: 78.8% Intervention; 76.7% Control Mean LVEF: 55.9% Intervention; 56.1% Control Type of MI: 51.1% Intervention; 51.6% Control	2271	Beta-blocker therapy prescribed at discharge (1696)	No beta-blocker therapy (575)	Primary outcome: all-cause mortality. Secondary outcome: cardiovascular mortality	Median 1017 days (IQR 401–1804)
Peck et al. (2020)^75^	Multi-centre retrospective cohort study	MIG Registry (Melbourne Interventional Group)	Patients with acute coronary syndrome who underwent PCI	Mean age: 63.2 Intervention; 65.4 Control Males: 76.9% Intervention; 73.4% Control Mean LVEF: Not reported Type of MI: 47.4% STEMI Intervention; 33.8% STEMI Control	17,562	Use of beta-blocker therapy at 30 days post-PCI (14,636)	No beta-blocker therapy (2926)	Primary outcome: All-cause mortality	Mean 5.3 years (SD ± 3.5 years)
Rochon et al. (2000)^76^	Multicenter retrospective cohort study	–	**This review only included a subpopulation:** post–MI patients aged ≥66 years who either did not receive beta-blocker therapy or received standard dose (defined as a dose achieved with available tablet sizes but less than the doses used in the RCTs)	Mean age: 73.6 Intervention; 76.8 Control Males: 56.8% Intervention; 53.9% Control Mean LVEF: Not reported Type of MI: Not reported	10,617	Beta-blocker therapy dispensed in the 365 days post-discharge (3068)	No beta-blocker therapy (7549)	Primary outcome: Admission for heart failure Secondary outcomes: All-cause mortality	1 year (endpoint)
Sakagami et al. (2023)^77^	Nationwide retrospective cohort study	J-MINUET study (Japanese Registry of Acute Myocardial Infarction Diagnosed by Universal Definition)	Post–MI patients without reduced LVEF (≥40%) who underwent primary PCI	Mean age: 67 Intervention; 68.6 Control Males: 77% Intervention; 75.8% Control Mean LVEF: 55.6% Intervention; 59.1% Control Type of MI: 78.6% STEMI Intervention; 70% STEMI Control	1923	Beta-blocker therapy prescribed at discharge (1353)	No beta-blocker therapy (570)	Primary outcome: composite of all-cause death, non-fatal MI, and non-fatal stroke. Secondary outcomes: all-cause death, cardiovascular death, non-fatal MI, non-fatal stroke, heart failure admission, unstable angina pectoris with revascularization, and the composite of all-cause death, non-fatal MI, non-fatal stroke, and heart failure admission.	3 years (endpoint)
Shavadia et al. (2019)^78^	Multicenter retrospective cohort study	CRUSADE Registry (Can Rapid Risk Stratification of Unstable Angina Patients Suppress Adverse Outcomes With Early Implementation of the American College of Cardiology/American Heart Association Guidelines)	Post–MI patients ≥65 years of age alive at 3 years without a recurrent MI	Mean age: 75 Intervention and Control Males: 44.8% Intervention; 50.9% Control Mean LVEF: Not reported Type of MI: 8.7% STEMI Intervention; 7.3% STEMI Control	6893	Use of beta-blocker therapy at 3 years after index MI (4980)	No user of beta-blocker therapy at 3 years after index MI (1913)	Primary outcome: Composite of all-cause mortality, hospitalisation for MI, hospitalisation for ischaemic stroke, or hospitalisation for heart failure. Secondary outcomes: individual components of primary outcome	Median 8 years (IQR 5.2–9.2)
Siu et al. (2010)^79^	Single centre prospective cohort study	–	Post-STEMI patients with preserved systolic function (LVEF ≥50%) and negative stress test	Mean age: 62 Intervention; 65 Control Males: 72% Intervention; 82% Control Mean LVEF: 55% Intervention; 54% Control Type of MI: All STEMI	208	Beta-blocker therapy prescribed at discharge (154)	No beta-blocker therapy (54)	Primary outcomes: All-cause mortality, cardiovascular mortality, sudden and non-sudden cardiovascular mortality.	Mean 58.5 months (±2.7 months)
Song et al. (2019)^80^	Multicenter retrospective cohort study	KAMIR-NIH Registry	Post–MI patients	Variables were reported for the full sample and different LVEF group and not per beta-blocker therapy. The following results correspond to the full sample: Mean age: 64 Males: 74.4% Mean LVEF: Not reported Type of MI: 44.5% STEMI	10,785	Beta-blocker therapy prescribed at discharge (9163)	No beta-blocker therapy (1622)	Primary outcome: all-cause mortality Secondary outcomes: MI, rehospitalization for heart failure, MACE (composite of all-cause mortality, MI, and rehospitalization for heart failure)	Median 733 days (IQR 698–760)
Yamada et al. (2006)^81^	Single-centre prospective cohort study	–	Patients with first MI without needing a coronary artery bypass graft	Mean age: 62 Intervention; 66 Control Males: 75% Intervention; 77% Control Mean LVEF: 54% Intervention and Control Type of MI: Not reported	546	Beta-blocker therapy prescribed at discharge (400)	No beta-blocker therapy (146)	Primary outcome: All-cause mortality	Mean 2.0 years (SD ± 1.1 years)

**Author (year)**	**Type of study**	**Name of study/registry**	**Population**	**Study participants in matched population: Age (in years), gender, mean LVEF, type of MI**	**Total sample size**	**Intervention definition (sample size)**	**Control definition (sample size)**	**Outcomes assessed**	**Follow-up**
PSM only									
Bangalore et al. (2012)^82^	Retrospective multicentre observational study	REACH (Reduction of Atherothrombosis for Continued Health)	**This review only included a subpopulation**: patients aged 45 years or older with known prior MI.	Mean age: 68.7 Intervention; 68.5 Control Males: 75.2% Intervention; 74.9% Control Mean LVEF: Not reported Type of MI: Not reported	Full cohort: 14,043 Matched cohort: 6758	User of beta-blocker therapy at time of enrolment (Full cohort: 9451; Matched cohort: 3379)	Non user of beta-blocker therapy at time of enrolment (Full cohort: 4592; Matched cohort: 3379)	Primary outcome: composite of cardiovascular death, nonfatal MI, or nonfatal stroke. Secondary outcomes: primary outcome plus hospitalisation for atherothrombotic events or a revascularization procedure. Tertiary outcomes: all-cause mortality, cardiovascular mortality, non-fatal MI, non-fatal stroke, and hospitalisation as separate outcomes.	Median 43 months (IQR 30–45)
D'Ascenzo et al. (2018)^83^	Retrospective multicentre observational study	BleeMACS (Bleeding complications in a Multicenter registry of patients discharged with diagnosis of Acute Coronary Syndrome)	Patients with acute coronary syndrome who underwent PCI	Mean age: 66 Intervention and Control Males: 67.6% Intervention; 73.7% Control Mean LVEF: 54% Intervention and control Type of MI: 60.4% STEMI Intervention; 55.8% STEMI Control	Full cohort: 15,210 Matched cohort: 5870	Beta-blocker therapy prescribed at discharge (Full cohort: 12,275; Matched cohort: 2935)	No beta-blocker therapy (Full cohort: 2935; Matched cohort: 2935)	Primary outcome: all-cause mortality Secondary outcomes: in-hospital reinfarction, in-hospital heart failure, 1-year myocardial infarction, 1-year bleeding and 1-year composite of death and recurrent myocardial infarction.	1 year (endpoint)
Joo et al. (2022)^84^	Retrospective multicentre observational study	KAMIR-NIH (The Korea Acute Myocardial Infarction Registry-National Institute of Health)	Post–MI patients with mildly reduced systolic function (LVEF: 41–49%)	Mean age: 66.4 Intervention; 66.8 Control Males: 70.1% Intervention; 70.5% Control Mean LVEF: 45.7% Intervention; 45.5% Control Type of MI: 51.7% STEMI Intervention; 52.9% STEMI Control	Full cohort: 2904 Matched cohort: 1048	Beta-blocker therapy prescribed at discharge (Full cohort: 2508; Matched cohort: 685)	No beta-blocker therapy (Full cohort: 396; Matched cohort: 363)	Primary outcome: 2-year major adverse cardiac events (composite of cardiac death, myocardial infarction, revascularization, and re-hospitalisation due to heart failure). Secondary outcomes: each component of MACE plus all-cause death, stroke, 2-year major adverse cardiac and cerebrovascular events (composite of the primary endpoint and stroke), and 2-year MACE with non-cardiac death.	2 years (endpoint)
Konishi et al. (2016)^85^	Retrospective single-centre observational study	–	First STEMI patients with preserved systolic function (LVEF >40%) who underwent PCI	Mean age: 64.3 Intervention; 64.9 Control Males: 80.6% Intervention; 80.6% Control Mean LVEF: 56.4% Intervention; 56.3% Control Type of MI: All STEMI	Full cohort: 424 Matched cohort: 206	Beta-blocker therapy prescribed at discharge (Full cohort: 197; Matched cohort: 103)	No beta-blocker therapy (Full cohort: 227; Matched cohort: 103)	Primary outcomes: major adverse cardiac events, all-cause death and cardiac death.	Median 4.7 years (IQR 1.2–6.4)
Lee et al. (2020)^86^	Retrospective multicentre observational study	HIRA (The Health Insurance Review & Assessment)	**This review only included a subpopulation:** patients aged ≥18 years post first MI who underwent PCI.	Mean age: 65 Intervention and Control Males: 75.2% Intervention and Control Mean LVEF: Not reported Type of MI: Not reported	Full cohort: 38,246 Matched cohort: 14,666	Beta-blocker therapy prescribed at discharge (Full cohort: 30,815; Matched cohort: 7333)	No beta-blocker therapy (Full cohort: 7431; Matched cohort: 7333)	Primary outcome: all-cause mortality.	Median 2.2 years (IQR 1.2–3.3)
Raposeiras-Roubin et al. (2015)^87^	Retrospective single-centre observational study	CardioCHUS	Patients with acute coronary syndrome and preserved left ventricular systolic function (≥50%)	Mean age: 66.1 Intervention; 66.2 Control Males: 67.2% Intervention; 70.5% Control Mean LVEF: Not reported Type of MI: 30.1% STEMI Intervention; 25.9% STEMI Control	Full cohort: 3236 Matched cohort: 1110	Beta-blocker therapy prescribed at discharge (Full cohort: 2277; Matched cohort: 555)	No beta-blocker therapy (Full cohort: 959; Matched cohort: 555)	Primary outcome: overall mortality	Median 5.2 years (IQR 2–7.2)
Won et al. (2020)^88^	Retrospective multicentre observational study	KNHIS (Korean National Health Insurance Service)	Post first AMI patients who underwent PCI	Mean age: 62 Intervention; 61 Control Males: 75.19% Intervention; 75.22% Control Mean LVEF: Not reported Type of MI: Not reported	Full cohort: 81,752 Matched cohort: 21,248	Regular user of beta-blocker therapy defined as medication possession ratio (MPR) ≥80% (Full cohort: 63,885; Matched cohort: 10,624)	Non user of beta-blocker therapy defined as MPR = 0% (Full cohort: 17,867; Matched cohort: 10,624)	Primary outcome: composite of all-cause death. Secondary outcomes: myocardial infarction and all type of stroke.	Median 24.33 months (IQR 18.03–24.33)
With and without PSM									
Al-Bawardy et al. (2024)^89^^,^^a^	Multicountry retrospective observational study	Seven Arabian Gulf countries registries	Patients' post-acute coronary syndrome with LVEF >40%	Mean age: 55.3 Intervention; 57.4 Control Males: 80.5% Intervention; 72.5% Control Mean LVEF: Not reported Type of MI: 50.8% STEMI Intervention; 44.5% STEMI Control	Full cohort: 18,399 Matched cohort: 954	Beta-blocker therapy prescribed at discharge (Full cohort: 15,541; Matched cohort: 477)	No beta-blocker therapy (Full cohort: 2798; Matched cohort: 477)	Primary outcome: all-cause mortality.	1 year (endpoint)
Chen et al. (2021)^90^	Retrospective single-centre observational study	–	Patients' post-acute coronary syndrome who underwent PCI, were not complicated with clinical heart failure and had preserved systolic function (LVEF ≥50%)	Mean age: 58.7 Intervention; 60 Control Males: 83.8% Intervention; 81.7% Control Mean LVEF: 57.6% Intervention; 58% Control Type of MI: 93.3% STEMI Intervention; 90.9% STEMI Control	Full cohort: 2397 Matched cohort: 656	Beta-blocker therapy prescribed at discharge (Full cohort: 2060; Matched cohort: 328)	No beta-blocker therapy (Full cohort: 337; Matched cohort: 328)	Primary outcome: all-cause death. Secondary outcome: cardiac death and major adverse cardiovascular event (composite of cardiac death, non-fatal myocardial infarction, and non-fatal stroke).	Median 727 days (IQR 433–2016)
Choo et al. (2014)^91^	Retrospective multicentre observational study	COREA-AMI (COnvergent REgistry of cAtholic and chonnAm university for Acute MI)	Post–MI patients with preserved systolic function (LVEF ≥50%) who underwent PCI	Mean age: 62.9 Intervention; 63 Control Males: 69.5% Intervention and Control Mean LVEF: 60.7% Intervention; 60.2% Control Type of MI: 57.5% STEMI Intervention; 57.2% STEMI Control	Full cohort: 3019 Matched cohort: 1182	Oral beta-blocker therapy received within the first 24 h and maintained for at least 1 month after discharge. (Full cohort: 2424; Matched cohort: 591)	No beta-blocker therapy (Full cohort: 595; Matched cohort: 591)	Primary outcome: all-cause mortality Secondary outcomes: cardiac death, MI, stroke, and coronary revascularisation procedures.	3 years (endpoint)
Jeong et al. (2024)^92^^,^^a^	Nationwide retrospective observational study	KAMIR V (The Korean Acute Myocardial Infarction Registry V)	Post–MI patients with preserved LVEF (≥40%)	Mean age: 62.9 Intervention; 64.7 Control Males: 79.1% Intervention; 77.7% Control Mean LVEF: 54.8% Intervention; 55.5% Control Type of MI: 50.9% STEMI Intervention; 42.6% STEMI Control	Full cohort: 12,101 Matched cohort: 5266	Beta-blocker therapy prescribed at discharge (Full cohort: 9468; Matched cohort: 2633)	No beta-blocker therapy (Full cohort: 2633; Matched cohort: 2633)	Primary outcome: composite including all-cause mortality, any MI, and any revascularization, at 1-year follow-up after PCI. Secondary outcomes: each component of primary outcome.	Median 353 days (IQR 194–378 days)
Joo et al. (2021)^93^	Retrospective multicentre observational study	KAMIR-NIH	Post–MI patients	Mean age: 65.3 Intervention; 65.5 Control Males: 73.4% Intervention; 73.6% Control Mean LVEF: 52.1% Intervention and control Type of MI: Not reported	Full cohort: 12,200 Matched cohort: 5015	Beta-blocker therapy prescribed at discharge (Full cohort: 10,251; Matched cohort: 3240)	No beta-blocker therapy (Full cohort: 1949; Matched cohort: 1775)	Primary outcome: 1-year major adverse cardiac events (composite of cardiac death, MI, revascularization, and readmission due to heart failure). Secondary outcomes: all-cause death, 1-year major adverse cardiac and cerebrovascular events (composite of the primary endpoint and stroke, and each component of primary outcome)	1 year (endpoint)
Nakatani et al. (2013)^94^	Retrospective multicentre observational study	OACIS (Acute Coronary Insufciency Study)	Patients admitted <24 h of the onset of STEMI who underwent PCI	Mean age: 64.4 Intervention; 65.1 Control Males: 77.8% Intervention; 76.4% Control Mean LVEF: Not reported Type of MI: All STEMI	Full cohort: 5628 Matched cohort: 3846	Beta-blocker therapy prescribed at discharge (Full cohort: 2880; Matched cohort: 1923)	No beta-blocker therapy (Full cohort: 2748; Matched cohort: 1923)	Primary outcome: all-cause death (which was categorised as cardiac, noncardiac, or unknown)	Median 1430 days (IQR 454–1794)
Park et al. (2018)^95^	Retrospective single-centre observational study	–	**This review only included a subpopulation:** post–MI patients discharged alive.	Mean age: 64.2 Intervention; 64.78 Control Males: 75.4% Intervention; 74.4% Control Mean LVEF: 53.7% Intervention; 51.7% Control Type of MI: 51.2% STEMI Intervention; 53.7% STEMI Control	Full cohort: 2592 Matched cohort: 1258	Beta-blocker therapy prescribed at discharge (Full cohort: 1880; Matched cohort: 629)	No beta-blocker therapy (Full cohort: 712; Matched cohort: 629)	Primary outcome: all-cause mortality.	Median 1364 days (IQR 784–2190)
Park et al. (2021)^96^	Nationwide retrospective observational study	KAMIR-NIH Registry	Patients with acute myocardial infarction (MI)	Mean age: 61.4 Intervention; 63.1 Control Males: 76.4% Intervention; 76.3% Control Mean LVEF: 52.9% Intervention; 51.3% Control Type of MI: 49.2% STEMI Intervention; 57.5% STEMI Control	Full cohort: 4008 Matched cohort: 1760	Use of beta-blocker therapy at 1-year follow-up (Full cohort: 3177; Matched cohort: 1320)	No user of beta-blocker therapy at 1-year follow-up (Full cohort: 831; Matched cohort: 440)	Primary outcome: all-cause mortality Secondary outcome: cardiovascular mortality	2 years (endpoint)
Puymirat et al. (2016)^97^	Retrospective multicentre observational study	FAST-MI (French registry of Acute ST-elevation and non-ST-elevation Myocardial Infarction)	**This review only included a subpopulation**: Patients without history of heart failure, Killip class I throughout the hospital stay and without documentation of an ejection fraction of 40% or below before hospital discharge who had received beta-blocker therapy at discharge and were alive at one year.	Mean age: 65.1 Intervention; 65.7 Control Males: 69% Intervention; 72% Control Mean LVEF: Not reported Type of MI: 60% STEMI Intervention; 59% STEMI Control	Full cohort: 1383 Matched cohort: 372	Persistent use of beta-blocker therapy at one year (Full cohort: 1230; Matched cohort: 277)	No beta-blocker therapy (Full cohort: 153; Matched cohort: 95)	Primary outcome: all-cause mortality.	5 years (endpoint)
Wen et al. (2022)^98^	Retrospective multicentre observational study	–	Patients' post-acute coronary syndrome without heart failure and with preserved ejection fraction (LVEF ≥50%).	Mean age: 63 Intervention; 64 Control Males: 79.2% Intervention; 80.2% Control Mean LVEF: 59% Intervention and Control Type of MI: 60.6% STEMI Intervention; 62% STEMI Control	Full cohort: 2519 Matched cohort: 848	Beta-blocker therapy prescribed at discharge (Full cohort: 2049; Matched cohort: 424)	No beta-blocker therapy (Full cohort: 470; Matched cohort: 424)	Primary outcome: all-cause mortality Secondary outcomes: all-cause rehospitalization, cardiac death, recurrent myocardial infarction, new-onset heart failure rehospitalization, and major adverse cardiovascular events (composite endpoint event of cardiac death, recurrent myocardial infarction, new-onset heart failure rehospitalization).	Median 3.61 years (IQR 2.12–5.27)
Yang et al. (2014)^99^	Retrospective multicentre observational study	KAMIR-NIH	Post-STEMI patients who underwent PCI	Mean age: 66 Intervention; 65 Control Males: 72.2% Intervention; 74.7% Control Mean LVEF: 50% Intervention and Control Type of MI: All STEMI	Full cohort: 8510 Matched cohort: 3975	Beta-blocker therapy prescribed at discharge (Full cohort: 6873; Matched cohort: 2650)	No beta-blocker therapy (Full cohort: 1637; Matched cohort: 1325)	Primary outcome: all-cause death. Secondary outcomes: cardiac death, recurrent MI, any revascularization with PCI or coronary artery bypass graft, and major adverse cardiac events (composite of all-cause death, recurrent MI, and any revascularization during follow-up).	Median 367 days (IQR 157–440)

RCT = Randomised Controlled Trial; MI = Myocardial Infarction; LVEF = Left Ventricular Ejection Fraction; STEMI = ST- Elevation Myocardial Infarction; NSTEMI = Non-ST- Elevation Myocardial Infarction; MACE = Major Adverse Cardiac Event; PCI = Percutaneous Coronary Intervention; IQR = Interquartile Range; CAG = Coronary Angiogram; CABG = Coronary Artery Bypass Grafting.

a Non-matched population characteristics reported.

Overall, 495,827 patients were included. 383,095 (77.3%) were beta-blocker users and 112,632 (22.7%) were non-users. Follow-up ranged from 1 to 8 years (median 2.77, interquartile range [IQR] 1.41 to 3.63 years). Participant characteristics were broadly similar across study designs (Table 3), though non-PSM studies showed greater imbalances, with controls being older, more often female and more likely to present with NSTEMI. RCTs and PSM studies had more comparable age, sex, and STEMI proportions, and mean LVEF was consistent across all designs. Type of MI and LVEF were unreported in 28.6% and 49.0% of studies, respectively.

**Table 3:** Summary of participant characteristics by study design.

Variable	Beta-blocker users	Non-users	Missing data
	n = 383,095	n = 112,632	n (%)
Mean Age			
RCTs	64.1	63	0
PSM	64.4	64	0
Non-PSM	64.3	68.9	2 (6.1)
Males (%)			
RCTs	79.5	79.6	0
PSM	74	74.8	0
Non-PSM	77.2	68.1	2 (6.1)
STEMI (%)			
RCTs	52.6	52.3	2 (22.2)
PSM	56	52.5	3 (42.9)
Non-PSM	50.9	42.8	9 (27.3)
Mean LVEF			
RCTs	54.7	54.8	4 (44.4)
PSM	52.5	53.2	4 (57.1)
Non-PSM	53.9	51.8	16 (48.5)

RCTs = Randomised Controlled Trials; PSM = Propensity Score Matching; Non-PSM = Non-Propensity Score Matching; LVEF = Left Ventricular Ejection Fraction.

### Meta-analysis

#### Randomised controlled trials

Seven RCTs contributed to the all-cause mortality analysis, encompassing 24,191 post–MI patients, of whom 12,099 (50.0%) received beta-blocker therapy. Beta-blocker therapy use was not significantly associated with all-cause mortality, although the point estimate suggested a modest reduction in risk (HR 0.89, 95% CI 0.78–1.01; Q = 9.01, df = 6, *p* = 0.08; I^2^ = 16.1%). Five RCTs contributed to the cardiovascular mortality analysis, including 18,173 post–MI patients, of whom 9065 (49.9%) received beta-blocker therapy. Similarly, beta-blocker therapy was not significantly associated with cardiovascular mortality, though the point estimate suggested a small reduction in risk (HR 0.94, 95% CI 0.74–1.19; Q = 5.20, df = 4, *p* = 0.27; I^2^ = 34.7%).

**Fig. 2:**
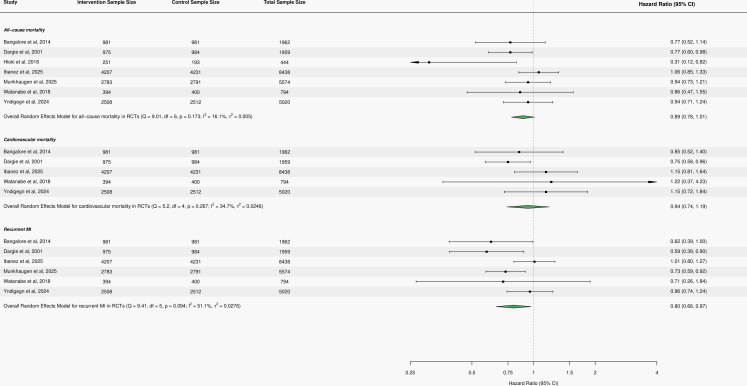
Forest plots of RCTs assessing the association between beta-blocker therapy and all-cause mortality, cardiovascular mortality, and recurrent MI. Caption: Forest plots of randomised controlled trials showing hazard ratios (HRs) for all-cause mortality, cardiovascular mortality, and recurrent MI in post–MI patients treated with beta-blocker therapy. The pooled random-effects estimates indicate no statistically significant association with all-cause mortality (HR 0.89, 95% CI 0.78–1.01) or cardiovascular mortality (HR 0.94, 95% CI 0.74–1.19), while beta-blocker therapy was associated with a significant reduction in recurrent MI (HR 0.80, 95% CI 0.66–0.97).

Six RCTs contributed to the recurrent MI analysis, comprising 23,747 post–MI patients, of whom 11,848 (49.9%) received beta-blocker therapy. In contrast to mortality outcomes, beta-blocker therapy was associated with a statistically significant reduction in recurrent MI, corresponding to an approximately 20% risk reduction (HR 0.80, 95% CI 0.66–0.97; Q = 9.41, df = 5, *p* = 0.09; I^2^ = 51.1%). Fig. 2 summarises the pooled results of the included randomised controlled trials.

#### Observational studies

Both PSM and non-PSM studies suggested lower all-cause (Supplemental Figure S1) and cardiovascular mortality (Supplemental Figure S2) among patients receiving beta-blocker therapy, with more pronounced effects observed in PSM studies; however, substantial heterogeneity was present across analyses. In contrast, findings for recurrent MI were inconsistent (Supplemental Figure S3): PSM studies were directionally concordant with RCTs, suggesting a non-significant reduction in risk, whereas non-PSM studies indicated a non-significant increase in risk.

Detailed sample sizes by study type for each outcome are presented in Supplemental Table S6.

#### Meta-regression analysis

**Fig. 3:**
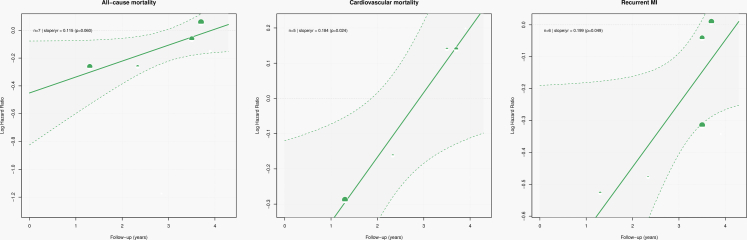
Meta-regression of follow-up duration and beta-blocker therapy treatment effects in RCTs. Caption: Meta-regression plots depicting the association between duration of follow-up (x-axis) and log hazard ratios for all-cause mortality, cardiovascular mortality, and recurrent MI in RCTs of beta-blocker therapy after MI. Each circle represents an individual trial, with size proportional to inverse-variance weight; the fitted regression line and 95% confidence band are shown. Longer follow-up was associated with attenuation of beta-blocker therapy effects on cardiovascular mortality (slope +0.18, *p* = 0.02), explaining 100% of between-study heterogeneity, and on recurrent MI (slope +0.20, *p* = 0.04), accounting for 67.5% of heterogeneity. For all-cause mortality, a trend toward attenuation with increasing follow-up was observed (slope +0.11, *p* = 0.06), explaining all heterogeneity. These findings suggest that the apparent benefits of beta-blocker therapy diminish over longer follow-up, particularly for cardiovascular outcomes.

Meta-regression analyses of RCTs indicated that longer follow-up duration was significantly associated with attenuation of the effect of beta-blocker therapy on cardiovascular mortality (slope +0.18, *p* = 0.02), explaining 100% of between-study heterogeneity, and on recurrent MI (slope +0.20, *p* = 0.04), explaining 67.5% of heterogeneity. For all-cause mortality, a similar but non-significant trend toward attenuation with longer follow-up was observed (slope +0.11, *p* = 0.06), accounting for all heterogeneity (Fig. 3). Meta-regression results from observational studies vary. In non-PSM studies, longer follow-up was significantly associated with modest attenuation of beta-blocker therapy effects on both all-cause and cardiovascular mortality (Supplemental Figure S4). In contrast, PSM studies showed no significant association between follow-up duration and treatment effects for any outcome (Supplemental Figure S5).

#### Subgroup analyses

Among patients with preserved LVEF (≥50%), RCTs showed no statistically significant association between beta-blocker therapy and all-cause mortality (HR 0.95, 95% CI 0.81–1.12; n = 5 studies, I^2^ = 1.4%), cardiovascular mortality (HR 1.16, 95% CI 0.88–1.52; n = 4 studies, I^2^ = 0%), or recurrent MI (HR 0.98, 95% CI 0.83–1.16; n = 4 studies, I^2^ = 0%). Only one RCT provided sufficient data for patients with midrange LVEF (40–49%), precluding subgroup analyses for this population across outcomes.

In observational studies, associations varied by LVEF subgroup and study design (Supplemental Table S7). Among patients with preserved LVEF, PSM analyses demonstrated significant risk reductions in all-cause mortality but no statistically significant associations with cardiovascular mortality or recurrent MI. Non-PSM studies in this subgroup showed no statistically significant associations for any outcome. For patients with midrange LVEF, PSM studies indicated significant risk reductions across all outcomes. Similarly, non-PSM analyses revealed significant risk reductions for all-cause mortality and recurrent MI in the midrange subgroup, while data for cardiovascular mortality were insufficient for meaningful analysis.

#### Risk of bias of included reviews

All 19 reviews clearly stated their review questions and inclusion criteria. Most employed appropriate search strategies (12/19, 63.2%),^29^, ^30^, ^31^, ^32^, ^33^, ^34^, ^35^^,^^38^^,^^39^^,^^41^^,^^42^^,^^46^ and ten used adequate sources (52.6%).^29^^,^^30^^,^^32^^,^^33^^,^^38^^,^^39^^,^^41^^,^^42^^,^^46^^,^^47^ Thirteen reviews (68.4%) applied suitable critical appraisal criteria,^29^, ^30^, ^31^, ^32^, ^33^, ^34^, ^35^^,^^39^^,^^40^^,^^43^, ^44^, ^45^^,^^47^ though only eight (42.1%) reported independent appraisal by two or more reviewers.^32^, ^33^, ^34^^,^^38^^,^^39^^,^^43^^,^^44^^,^^47^ Twelve reviews described methods to minimise data extraction errors (63.2%).^32^, ^33^, ^34^^,^^36^, ^37^, ^38^, ^39^^,^^42^, ^43^, ^44^^,^^46^^,^^47^ Eighteen reviews used appropriate synthesis methods (94.7%),^29^, ^30^, ^31^, ^32^, ^33^, ^34^, ^35^, ^36^, ^37^, ^38^, ^39^, ^40^, ^41^, ^42^, ^43^, ^44^, ^45^^,^^90^ while ten (52.6%) assessed publication bias.^29^^,^^30^^,^^32^^,^^33^^,^^39^^,^^41^, ^42^, ^43^^,^^45^^,^^46^ Sixteen reviews (84.2%) provided evidence-based recommendations,^29^, ^30^, ^31^, ^32^, ^33^, ^34^, ^35^, ^36^, ^37^, ^38^, ^39^^,^^41^, ^42^, ^43^^,^^45^^,^^47^ and suggested directions for future research.^29^, ^30^, ^31^, ^32^, ^33^, ^34^, ^35^, ^36^, ^37^, ^38^, ^39^^,^^41^^,^^43^, ^44^, ^45^^,^^47^

Based on preestablished categorisation, ten reviews (52.6%) were rated as high quality^29^^,^^30^^,^^32^, ^33^, ^34^^,^^38^^,^^39^^,^^41^^,^^43^^,^^47^; seven (36.8%) as moderate,^31^^,^^35^, ^36^, ^37^^,^^42^^,^^44^^,^^45^ and two (10.3%) as low^40^^,^^46^ (Supplemental Table S8).

#### Risk of bias of included primary studies

**Fig. 4:**
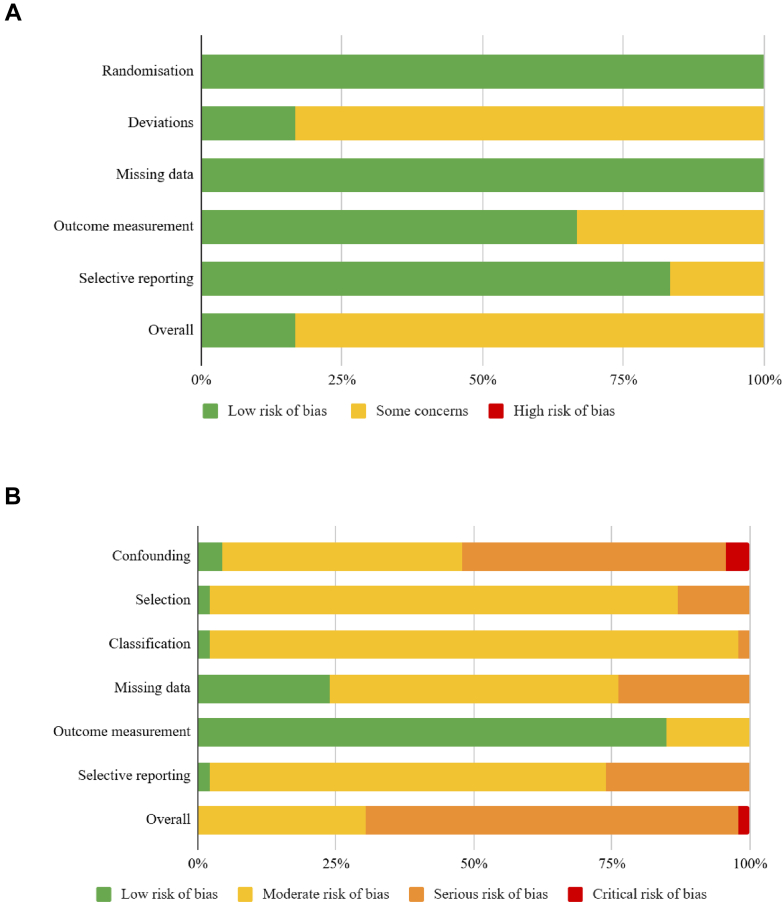
Risk of bias summary across included primary studies. Caption: Panel (A) presents domain-level risk of bias judgements for randomised controlled trials assessed using the Cochrane Risk of Bias 2 (RoB 2) tool. Panel (B) presents domain-level risk of bias judgements for observational studies assessed using the Risk Of Bias In Non-randomised Studies of Interventions (ROBINS-I) tool. Data are expressed as the percentage of studies within each risk of bias category.

Among the six included RCTs, overall risk of bias was low in one study^50^ and classified as “some concerns” in the remaining five^52^^,^^54^, ^55^, ^56^, ^57^; no study was rated as high risk of bias. All trials were at low risk for the randomisation process and missing outcome data.^50^^,^^52^^,^^54^, ^55^, ^56^, ^57^ Some concerns were most frequently identified in deviations from intended interventions,^52^^,^^54^, ^55^, ^56^, ^57^ with additional concerns in outcome measurement^55^^,^^56^ and selective reporting in a small number of studies.^56^ A summary of domain-level risk of bias judgements for RCTs is presented in Fig. 4 (A).

In contrast, observational studies were predominantly at serious risk of bias,^58^, ^59^, ^60^, ^61^, ^62^, ^63^, ^64^, ^65^, ^66^^,^^68^, ^69^, ^70^, ^71^, ^72^, ^73^, ^74^, ^75^, ^76^, ^77^, ^78^, ^79^^,^^81^, ^82^, ^83^, ^84^, ^85^^,^^87^^,^^88^^,^^95^, ^96^, ^97^ with a smaller proportion rated as moderate^67^^,^^86^^,^^89^, ^90^, ^91^, ^92^, ^93^, ^94^^,^^98^^,^^99^ and none at low overall risk. Studies using propensity score matching demonstrated comparatively improved methodological quality, though residual confounding remained.

Selection of participants and classification of interventions were generally rated as moderate risk, while missing data and outcome measurement were typically at low risk. Selective reporting was frequently rated as moderate to serious. A small number of studies were classified as having critical risk of bias, primarily driven by concerns in confounding and selection domains. Post hoc analyses were also included and generally demonstrated serious risk of bias, particularly related to confounding and selective reporting, reflecting their exploratory nature.^7^^,^^48^^,^^51^^,^^53^

A summary of domain-level risk of bias judgements for observational studies is presented in Fig. 4 (B). Detailed study-level assessments are provided in Supplemental Tables S9 and S10.

#### Certainty of evidence

Across outcomes, the GRADE certainty of evidence was limited, primarily due to reliance on observational studies, which led to frequent downgrading for risk of bias and inconsistency. Imprecision was a common limitation, while publication bias was frequently suspected or not assessed.

For all-cause mortality (n = 19), eight reviews were rated as having “moderate” certainty,^30^^,^^33^^,^^35^, ^36^, ^37^^,^^41^, ^42^, ^43^ eight as “low”,^29^^,^^32^^,^^34^^,^^38^, ^39^, ^40^^,^^45^^,^^46^ and three as “very low”^31^^,^^44^^,^^47^ (Supplemental Tables S11 and S12). For cardiovascular mortality (n = 14), five were rated as “moderate” certainty,^30^^,^^33^^,^^35^^,^^36^^,^^42^ five as “low”,^34^^,^^40^^,^^41^^,^^45^^,^^46^ and four had “very low”^32^^,^^38^^,^^44^^,^^47^ (Supplemental Tables S13 and S14). For recurrent MI (n = 13), seven were rated as “moderate”,^32^^,^^35^, ^36^, ^37^^,^^41^^,^^42^^,^^45^ and six as “low”^30^^,^^33^^,^^34^^,^^38^^,^^40^^,^^44^ (Supplemental Tables S15 and S16). The reviews by Safi et al.,^32^^,^^33^ which included exclusively RCTs, consistently received lower certainty ratings due to serious indirectness (younger pre-reperfusion era populations), and high risk of bias, as assessed by authors with the Cochrane tool.

#### Overlap analysis

Significant overlap was noted between the 19 systematic reviews, which included 52 unique primary studies (Supplemental Table S17). This yielded a CCA of 15.1%, indicating high overlap (Supplemental Figure S6). Among primary studies, two (3.9%)^56^^,^^91^ appeared in half or more (≥10) of the 19 systematic reviews. A further three studies (5.8%)^57^^,^^70^^,^^85^ appeared in nine reviews, one (1.9%)^99^ in eight reviews, and four (7.7%)^60^^,^^73^^,^^94^^,^^97^ in seven reviews, indicating a core group of frequently cited evidence. In contrast, 13 studies (25%) were cited in only one review.^59^^,^^61^^,^^62^^,^^68^^,^^72^^,^^75^^,^^77^^,^^80^^,^^86^^,^^88^^,^^89^^,^^92^^,^^96^ This high overlap indicates substantial redundancy across reviews, potentially inflating the perceived strength of evidence. By focussing only on unique primary studies in our analysis, we minimised double-counting and ensured methodological consistency.

#### Sensitivity analyses

Sensitivity analyses excluding studies with converted effect estimates were performed only for observational studies, as primary RCTs did not require conversion. In non-PSM studies, all-cause mortality showed a modest shift in the pooled HR from 0.78 to 0.83 (5.9% change) after excluding three studies, with statistical significance maintained. Cardiovascular mortality changed minimally, with the HR reducing from 0.80 to 0.78 (2.3% change), after excluding one study, and recurrent MI was essentially unchanged (<0.5% change), with the association remaining non-significant. In PSM studies, exclusion of a single study led to a slight change in all-cause mortality HR from 0.70 to 0.71 (2.2% change) with statistical significance preserved. Heterogeneity remained largely stable across all outcomes. These analyses confirm the robustness of the observational results. Detailed comparisons are provided in Supplemental Table S18.

Validation analyses based on systematic reviews and meta-analyses revealed significant protective associations of beta-blocker therapy with all-cause mortality (Odds Ratio [OR] 0.80, 95% CI 0.74–0.86; I^2^ = 76.3%), cardiovascular mortality (OR 0.81, 95% CI 0.72–0.92; I^2^ = 71.0%), and recurrent MI (OR 0.90, 95% CI 0.84–0.97; I^2^ = 66.6%) (Supplemental Figure S7). These results showed greater effect sizes and higher heterogeneity compared with the RCT data.

The discrepancy between RCT findings and validation analyses likely reflects the substantial contribution of observational studies in the published reviews, which tend to report larger effect estimates and exhibit greater heterogeneity. Given the superior internal validity of RCTs, these findings were prioritised in interpreting the efficacy of beta-blocker therapy, with the validation analysis providing contextual support rather than confirmatory evidence.

Finally, leave-one-out analyses demonstrated that pooled estimates were generally stable across study designs, with no single study materially altering the direction of effect estimates. Variability was more pronounced in observational studies, supporting stratification by study design, while RCT estimates remained comparatively stable. Within the RCT subgroup, sensitivity was primarily driven by Dargie et al.,^50^ a trial including patients with reduced LVEF, suggesting that observed variability reflects differences in underlying populations rather than instability of pooled estimates.

For recurrent MI, Ibáñez et al.,^52^ had the greatest influence, likely reflecting its larger sample size and corresponding statistical weight. Across outcomes, exclusion of individual studies resulted in only modest changes in effect sizes, with occasional changes in statistical significance but no consistent alteration in the direction of effect. Detailed results of the leave-one-out analyses are presented in Supplementary Table S19.

Overall, these sensitivity analyses confirm the robustness of the findings while highlighting the influence of study design, population characteristics, and residual confounding on effect estimates.

#### Publication bias

**Fig. 5:**
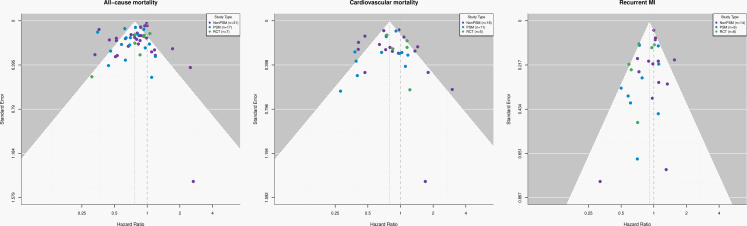
Funnel plots stratified by study type for all-cause mortality, cardiovascular mortality and recurrent MI. Caption: Funnel plots of hazard ratios (HRs) versus standard error for all-cause mortality, cardiovascular mortality, and recurrent myocardial infarction, stratified by study design. For all-cause mortality, no evidence of small-study effects was observed in RCTs (Egger's *p* = 0.08; Begg's *p* = 0.14; n = 7) or PSM studies (Egger's *p* = 0.78; Begg's *p* = 0.33; n = 18), whereas asymmetry was detected in non-PSM studies (Egger's *p* = 0.04; Begg's *p* = 0.83; n = 32). For cardiovascular mortality, Egger's and Begg's tests were non-significant across all study designs (RCTs: *p* = 0.35 and 1.00; PSM: *p* = 0.15 and 0.45; non-PSM: *p* = 0.74 and 0.37). Similarly, no evidence of small-study effects was observed for recurrent myocardial infarction (RCTs: *p* = 0.34 and 1.00; PSM: *p* = 0.34 and 1.00; non-PSM: *p* = 0.76 and 0.92). Overall, visual symmetry in RCT and PSM plots and the largely unchanged pooled estimates after trim-and-fill adjustment support the robustness of the findings, with caution warranted for non-PSM analyses of all-cause mortality.

Egger's and Begg's tests showed no evidence of small-study effects across RCTs and PSM study types for all outcomes. Asymmetry was detected for non-PSM studies for all-cause mortality (*p* =0.04) Trim-and-fill analyses suggested minimal impact on pooled estimates, with adjusted HRs remaining consistent with original findings. In PSM and RCT studies for recurrent MI, and in non-PSM studies for cardiovascular mortality, trim-and-fill adjustment shifted effect estimates modestly toward the null, in some cases resulting in loss of statistical significance; however, the direction of effect was unchanged. Overall, these results indicate that pooled estimates are generally robust, with limited influence of potential publication bias across outcomes, although some results, particularly in non-PSM analyses, may be sensitive to small-study effects or the influence of individual studies (Supplemental Table S20). Visual inspection of funnel plots demonstrated largely symmetrical distributions for RCTs and PSM studies across all outcomes, with effect estimates evenly dispersed around the pooled effects. Conversely, non-PSM studies for all-cause mortality showed noticeable asymmetry, consistent with small-study effects likely related to residual confounding rather than selective non-publication (Fig. 5).

## Discussion

To our knowledge, this umbrella review provides the most comprehensive evaluation to date of beta-blocker therapy effectiveness post–MI in the reperfusion era, synthesising data from 19 systematic reviews and 52 primary studies encompassing 495,827 patients across 34 countries. By re-analysing primary study data, we were able to explore heterogeneity by study design and LVEF subgroups, providing a more nuanced understanding than prior meta-analyses.

Meta-analysis across seven RCTs including 24,191 patients provides the most rigorous evidence on beta-blocker therapy after MI. Across all RCTs, beta-blocker therapy was not significantly associated with all-cause mortality (HR 0.89, 95% CI 0.78–1.01) or cardiovascular mortality (HR 0.94, 95% CI 0.74–1.19), aligning with previous systematic reviews.^31^^,^^34^^,^^36^^,^^37^ In contrast, beta-blocker therapy was associated with a significant 20% reduction in recurrent MI (HR 0.80, 95% CI 0.66–0.97), confirming a protective effect in preventing reinfarction, consistent with prior reviews.^32^^,^^42^

Analyses of observational studies, particularly PSM analyses, generally showed effect estimates in the same direction as RCTs, although statistical significance varied. This alignment suggests that well-designed observational studies can provide complementary evidence, especially for populations and outcomes under-represented in trials. Non-PSM studies tended to show larger and more heterogeneous effects, reflecting residual confounding, variable dosing, adherence differences, and variation across populations and settings. Real-world patients are often older and have more comorbidities, which may lead to greater absolute benefit compared with RCTs participants. While these studies provide valuable insight into real-world outcomes, they should be interpreted cautiously and primarily as context to inform clinical decision-making rather than as confirmatory evidence.

Discrepancies between RCTs and observational studies for cardiovascular mortality likely reflect several factors. First, era-dependent treatment effects: early trials showed cardiovascular mortality reduction through prevention of arrhythmias and rupture,^100^ whereas contemporary deaths are more often attributable to heart failure and non-ischaemic causes, where beta-blocker therapy may confer less incremental benefit.^101^^,^^102^ Second, observational studies often capture longer treatment durations, higher-risk patients, and reflect real-world dosing strategies, unlike controlled RCTs.^103^ Third, inclusion of patients with preserved LVEF and NSTEMI in modern RCTs may dilute the observed treatment effect, as these subgroups derive more modest benefit from beta-blocker therapy.^56^

Extending our main findings, meta-regression analysis exploring study-level follow-up suggests a key temporal pattern in the effectiveness of beta-blocker therapy. In RCTs, the reduction in cardiovascular mortality and recurrent MI associated with beta-blocker therapy attenuated over longer follow-up, indicating that the greatest benefit occurs early after MI (<2 years), aligning with acute phase cardioprotective mechanisms.^104^ Observational non-PSM analyses mirrored this attenuation, whereas PSM studies showed less dependence on follow-up duration, highlighting the confounding effects of treatment selection, adherence, and survivorship bias in real-world data. Potential drivers of this attenuation include ventricular remodelling completion and β-receptor desensitisation,^105^, ^106^, ^107^ treatment discontinuation and evolving background therapies.^108^ While this analysis is exploratory and should be interpreted with caution, it highlights the potential need for ongoing monitoring and reassessment of therapy in long-term survivors.

Subgroup analyses by LVEF revealed clinically important differences. In RCTs, beta-blockers showed no significant benefit for patients with preserved LVEF (≥50%), while data for midrange LVEF (40–49%) were limited. Observational studies suggest that midrange LVEF patients may experience the greatest reductions recurrent MI. Exploratory analyses indicated a 56–57% risk reduction in this subgroup, possibly due to greater neurohormonal activation in moderately reduced systolic function.^109^

Our findings are largely consistent with recent RCT evidence. The REDUCE-AMI^57^ and REBOOT^52^ trials, which evaluated beta-blockers in patients with preserved LVEF post–MI, found no significant reductions in all-cause mortality, aligning with our subgroup analyses that showed minimal benefit in patients with preserved LVEF. Conversely, evidence from the BETAMI-DANBLOCK^54^ trials suggests greater reductions in mortality and recurrent MI among patients with midrange LVEF, which is also reflected in our subgroup analysis results of observational studies. These patterns highlight that the clinical benefit of beta-blocker therapy may be heterogeneous, providing limited advantage in patients with preserved LVEF but more consistent benefit in those with midrange LVEF and underscore that contemporary post–MI management requires a more nuanced, individualised approach. Current guideline recommendations, which remain heavily influenced by historical trials,^110^, ^111^, ^112^, ^113^, ^114^, ^115^, ^116^ may require refinement. In practice, emphasis should be placed on early initiation of beta-blocker therapy in the acute phase, an LVEF-stratified approach to long-term therapy, and periodic re-assessment of ongoing need, particularly in patients with preserved systolic function.

It is important to consider the quality and certainty of the evidence when interpreting these findings. Only 10 systematic reviews (52.6%) were rated as high methodological quality, with most demonstrating appropriate search strategies, synthesis methods, and evidence-based conclusions. However, important limitations remained, particularly in the assessment of publication bias and transparency in data extraction processes. Despite this, the overall certainty of evidence for the effectiveness of beta-blocker therapy following myocardial infarction was low to moderate.

GRADE assessments, conducted at the level of the systematic reviews, indicated low to moderate certainty of evidence across outcomes. Downgrading was primarily driven by risk of bias and inconsistency, reflecting limitations in the underlying primary studies synthesised within each review. While RCT evidence was generally at low risk of bias, with only minor concerns unlikely to materially influence effect estimates, the evidence base was predominantly composed of observational studies at moderate to serious risk of bias. Residual confounding was the principal limitation, even among PSM analyses, alongside additional concerns related to participant selection, intervention classification, and selective reporting. These limitations reduce confidence in pooled estimates and contribute directly to lower certainty ratings.

In addition to these concerns, imprecision further limited confidence in the evidence, with wide confidence intervals frequently crossing the null in both RCT and observational meta-analyses. Publication bias was also commonly suspected or not formally assessed. Notably, even reviews restricted to RCTs were downgraded for indirectness, reflecting differences between historical trial populations and contemporary clinical settings. Furthermore, evidence from non-PSM studies suggested small-study effects for all-cause mortality, indicating potential overestimation of treatment effects.

Beyond these methodological considerations, several broader limitations in the literature were identified. Study designs ranged from rigorously conducted RCTs to observational analyses with heterogeneous populations and inherent biases. Variability in follow-up duration may contribute to attenuation of beta-blocker effects over time, while inconsistent definitions of LVEF subgroups limit comparability across clinically relevant strata. Differences in confounder adjustment further complicate interpretation of findings.

Taken together, these findings suggest that confidence in the effectiveness of post–MI beta-blocker therapy is constrained not by the conduct of the reviews themselves, but by the limited internal validity, indirectness, and redundancy of the underlying evidence base.

Within this context, this umbrella review has several strengths. It synthesises evidence from systematic reviews and meta-analyses, encompassing RCTs and observational studies, including PSM analyses. Consistent with principles of evidence hierarchy, clinical conclusions in this review are based primarily on RCTs, with observational analyses presented to contextualise findings, explore heterogeneity, and inform clinical scenarios not adequately addressed by existing trials. Our methodological rigour and focus on disaggregated LVEF subgroups enhance the clinical relevance and robustness of findings.

Nevertheless, several limitations should be acknowledged. Substantial heterogeneity, particularly among observational studies, reflects variability in patient populations, treatment protocols, follow-up duration, and confounder adjustment. Inconsistent reporting and classification of LVEF limited subgroup analyses, while variations in outcomes definitions impeded comparisons for certain endpoints. Although umbrella reviews are inherently susceptible to omission of primary studies not captured by included reviews, this risk was mitigated through an updated search conducted to December 2025, independent screening of all primary studies within eligible reviews against predefined inclusion criteria, and incorporation of newly published trials. Despite these measures, residual risk of missed evidence cannot be entirely excluded.

To maintain methodological consistency, we excluded studies reporting only composite outcomes, such as the ABYSS trial, diverging from some prior reviews. Finally, this review did not reassess the quality of individual primary studies, instead relying on the critical appraisals from included systematic reviews, some of which were missing or varied in rigour and consistency.

Future research should prioritise adequately powered, pragmatic head-to-head trials comparing beta-blocker therapy with alternative treatments in patients with preserved and midrange LVEF to reduce reliance on observational data. Standardising outcome measures and LVEF subgroup definitions, aligned with established guidelines, would enhance comparability, while examining long-term and patient-centred outcomes, including quality of life and functional status, would clarify real-world impact of beta-blocker therapy.

Additionally, although some primary studies stratified outcomes by STEMI versus NSTEMI, reporting was inconsistent. Consequently, while overall trends for beta-blocker therapy appear similar across MI types, formal subgroup analyses could not be performed. Future trials and observational studies should explicitly report MI type to enable more precise therapy recommendations for contemporary post–MI populations. Finally, developing predictive tools to identify patients most likely to benefit from beta-blocker therapy could support more personalised, evidence-based clinical decision-making.

In conclusion, this umbrella review provides the most comprehensive contemporary synthesis on beta-blocker therapy following MI. Findings indicate that while beta-blocker therapy reduces recurrent MI, its mortality benefit is less certain in modern populations, with effectiveness attenuating over longer follow-up and appearing limited to specific LVEF subgroups of the population. These findings underscore the importance of individualised, phenotype-stratified treatment decisions and highlight the need for updated clinical guidelines that reflect current evidence.

## Contributors

PCM and ST conceptualised the study and led the investigation and manuscript writing. DG, AE, LR, DS, SZ, and DMK contributed to drafting the initial manuscript. PCM and LR developed the search strategy. PCM, PKC, and AE conducted article screening. PCM, PKC, and SC assessed the quality of evidence and applied the GRADE approach. PCM, PKC, and SC contributed to data curation. PCM performed the data analysis, which was verified by HK. PCM, PKC, SC, HK, and ST had access to and verified the study data. All authors had full access to all data and accept responsibility for the decision to submit the manuscript for publication.

## Data sharing statement

Datasets generated and/or analysed during the current study are available from the corresponding author upon reasonable request.

## Declaration of interests

We declare no competing interests.
