# Supplementary material for: Beta-blocker therapy after myocardial infarction: an umbrella review
Source: eClinicalMedicine. 2026 May 30;96:103990. doi: 10.1016/j.eclinm.2026.103990 (PMC13240767; doi:10.1016/j.eclinm.2026.103990)
Supplement: Supplementary Material [file mmc1.docx]

**Supplementary Material**

**Beta-blocker therapy after myocardial infarction: An umbrella review**

Table of Contents

[Supplemental methods 4](#_Toc228913224)

[Meta-Regression 4](#_Toc228913225)

[Subgroup analysis 4](#_Toc228913226)

[Validation analysis 4](#_Toc228913227)

[Supplemental Table 1: Complete search strategy 5](#_Toc228913228)

[Supplemental Table 2: Data extracted from systematic reviews and primary studies 6](#_Toc228913229)

[Supplemental Table 3: Extra data extracted from primary studies 6](#_Toc228913230)

[Supplemental results 7](#_Toc228913231)

[Supplemental Table 4: Systematic reviews excluded after full-text screening. 7](#_Toc228913232)

[Supplemental Table 5: Excluded primary studies. 8](#_Toc228913233)

[Supplemental Figure 1: Stratified Forest plot of observational studies for all-cause mortality. 13](#_Toc228913234)

[Supplemental Figure 2: Stratified Forest plot of observational studies for cardiovascular mortality. 14](#_Toc228913235)

[Supplemental Figure 3: Stratified Forest plot of observational studies for recurrent MI. 15](#_Toc228913236)

[Supplemental Table 6: Sample sizes used in stratified meta-analyses. 16](#_Toc228913237)

[Supplemental Figure 4: Meta-regression of follow-up duration and beta-blocker therapy treatment effects in non-PSM studies. 17](#_Toc228913238)

[Supplemental Figure 5: Meta-regression of follow-up duration and beta-blocker therapy treatment effects in PSM studies. 18](#_Toc228913239)

[Supplemental Table 7: Associations of beta-blocker therapy with all-cause mortality, cardiovascular mortality, and recurrent MI in observational studies, stratified by LVEF subgroup. 19](#_Toc228913240)

[Supplemental Table 8: Quality of evidence assessment using the JBI tool. 20](#_Toc228913241)

[Supplemental Table 9: Domain-level risk of bias assessment of included randomised controlled trials using the Cochrane Risk of Bias 2 (RoB 2) tool 22](#_Toc228913242)

[Supplemental Table 10: Domain-level risk of bias assessment of included non-randomised studies using the Risk Of Bias In Non-randomised Studies of Interventions (ROBINS-I) tool. 23](#_Toc228913243)

[Supplemental Table 11: Certainty of evidence for all-cause mortality. 26](#_Toc228913244)

[Supplemental Table 12: GRADE assessment details for all-cause mortality. 29](#_Toc228913245)

[Supplemental Table 13: Certainty of evidence for cardiovascular mortality. 30](#_Toc228913246)

[Supplemental Table 14: GRADE assessment details for cardiovascular mortality. 32](#_Toc228913247)

[Supplemental Table 15: Certainty of evidence for recurrent MI. 33](#_Toc228913248)

[Supplemental Table 16: GRADE assessment details for recurrent MI. 35](#_Toc228913249)

[Supplemental Table 17: Mapping of primary studies included in the systematic reviews. 36](#_Toc228913250)

[Supplemental Figure 6: Heatmap of pairwise overlap of primary studies between included systematic reviews. 39](#_Toc228913251)

[Supplemental Table 18: Results for sensitivity analyses. 40](#_Toc228913252)

[Supplemental Figure 7: Forest plot for all-cause mortality, cardiovascular mortality and recurrent MI 41](#_Toc228913253)

[Supplemental Table 19: Results for leave-one-out analyses 42](#_Toc228913254)

[Supplemental Table 20: Summary of Egger’s and Begg’s tests and trim-and-fill analyses for all outcomes. 43](#_Toc228913255)

[References 44](#_Toc228913256)

Supplemental methods

Meta-Regression

The meta regression model was specified as:

*θᵢ = β₀ + β₁ × (follow-up timeᵢ) + εᵢ* where θᵢ represents the true effect size for study i, β₀ is the intercept, β₁ is the slope coefficient representing the change in log hazard ratio per additional year of follow-up, and εᵢ is the random error term. The proportion of between-study variance explained by follow-up time was calculated as R² = (τ²₀ - τ²₁)/τ²₀, where τ²₀ represents residual heterogeneity from the null model and τ²₁ represents residual heterogeneity after including the moderator. The significance of the moderator was tested using the Wald-type test (QM statistic).^1^

Subgroup analysis

Where available, the mean LVEF values for the intervention and control groups reported in primary studies were used for categorisation. If mean values were not reported but studies conducted subgroup analyses based on pre-specified LVEF ranges that align with our definitions, those groupings were used. If neither mean values nor subgroup analyses were provided, but an LVEF-based inclusion criterion was reported, this was used for categorisation, unless the criterion was ambiguous (for example, LVEF ≥40%, which could indicate either midrange or preserved function). Studies with ambiguous LVEF categorisation or no reported LVEF values were excluded from the subgroup analyses. A minimum of two studies per subgroup was required to estimate pooled effects and heterogeneity, with analyses further stratified by study design. This threshold supported exploratory rather than definitive subgroup inferences, given the limited power of small study numbers.

Validation analysis

To assess the consistency of our primary meta-analytic findings, we conducted validation analyses using aggregate summary estimates reported in the included systematic reviews and meta-analyses. These review-level results were analysed independently and not pooled with the primary study-level data. Included reviews reported effect estimates with varying confidence interval (CI) levels which, if not standardised, can result in misestimation of standard errors and weighting in meta-analysis. To enable appropriate synthesis, all estimates were recalibrated to a 95% CI level using z-score–based transformation.^2^ This approach preserved the underlying precision of the original estimate while enabling valid inverse-variance weighting across studies.

To allow pooled analysis, all reported effect estimates were converted to odds ratios (OR). Where systematic reviews reported hazard ratios (HRs) or relative risks (RRs), conversion was performed using established formulas.^3^ Clinical baseline risks were derived from relevant epidemiological data and set at 3% for all-cause mortality, 5% for cardiovascular mortality, and 8% for recurrent MI. Following standardisation, a random-effects meta-analysis was conducted using the DerSimonian and Laird method to pool effect estimates across systematic reviews.^4^ Heterogeneity was assessed using the I² statistic. Forest plots were generated to visually compare these pooled review-level estimates with those derived from the primary study-level analysis

**Supplemental Table 1: Complete search strategy**

**Supplemental Table 2: Data extracted from systematic reviews and primary studies**

| **Data from systematic reviews and primary studies** |
| --- |
| Citation details: Author, year, country |
| Study characteristics: Inclusion/exclusion criteria, databases searched, date range of included studies. |
| Intervention and control definitions |
| Statistical methods: Methods used, heterogeneity assessment, publication bias evaluation. |
| Methodological quality assessment: Tools and results |
| Certainty of evidence assessment: Tools and results |
| Sample demographics: Mean age, percentage of male participants, type of MI |
| Study outcomes: Total sample size, intervention and control group sizes, mean follow-up time, measure of association (type and value), confidence intervals |
| Study types and distribution: Ratio of observational studies to RCTs, mapping of primary studies |

MI = Myocardial Infarction; RCTs = Randomised Controlled Trials.

**Supplemental Table 3: Extra data extracted from primary studies**

| **Extra data from primary studies** |
| --- |
| Citation details: Study/registry name |
| Study characteristics: Study type (RCT, PSM or Non-PSM) |
| Sample demographics: Mean age, % male participants, type of MI, LVEF |

RCT = Randomised Controlled Trial; PSM = Propensity Score Matching; Non-PSM = Non-Propensity Score Matching; MI = Myocardial Infarction; LVEF = Left Ventricular Ejection Fraction.

Supplemental results

**Supplemental Table 4: Systematic reviews excluded after full-text screening.**

| **Author (Year)** | **Reason for exclusion** |
| --- | --- |
| Arero et al. (2021)^5^ | Systematic review with wrong patient population: only included patients without prior MI. |
| Ahmed et al. (2025)^6^ | Systematic review published as a letter to the editor. |
| Chi et al. (2024)^7^ | Abstract Poster |
| Chi et al. (2024)^8^ | Abstract Poster |
| Chi et al. (2025)^9^ | Abstract Poster |
| Clemente et al. (2024)^10^ | Abstract Poster |
| Dar and Jacob (2024)^11^ | Narrative review |
| De Filippo et al. (2022)^12^ | Systematic review with wrong intervention: it assessed the impact of multiple secondary prevention therapies instead of only beta-blocker therapy. |
| Desta et al. (2021)^13^ | Editorial |
| Gomez et al. (2025)^14^ | Abstract Poster |
| Harari and Bangalore (2020)^15^ | Editorial |
| Irtaza et al. (2024)^16^ | Systematic review not certified by peer review. |
| Jain et al. (2025)^17^ | Abstract Poster |
| Jansky et al. (2019)^18^ | Narrative review |
| Johner et al. (2024)^19^ | Narrative review |
| Lanham et al. (2019)^20^ | Narrative review |
| Martinez-Milla et al. (2019)^21^ | Narrative review |
| Ottani et al. (2022)^22^ | RCT protocol |
| Rosa et al. (2025)^23^ | Abstract Poster |
| Sabina et al. (2025)^24^ | Reply to letter to the editor |
| Vutthikraivit et al. (2019)^25^ | Abstract Poster |
| Zamiri et al. (2020)^26^ | Abstract Poster |
| Zamiri et al. (2020)^27^ | Systematic review with wrong patient population: included patients with acute coronary syndrome, not MI. |
| Zeitouni et al. (2019)^28^ | Narrative review |
| Zhang et al. (2021)^29^ | Protocol for systematic review comparing beta-blocker agents. |
| Zhao et al. (2020)^30^ | Narrative review |

RCTs = Randomised Controlled Trials; MI = Myocardial Infarction.

**Supplemental Table 5: Excluded primary studies.**

| **Author  (year)** | **Type of study** | **Population** | **Reason for exclusion** | **Total sample size** | **Results** |
| --- | --- | --- | --- | --- | --- |
| **RCTs** |  |  |  |  |  |
| Chen et al.  (2005)^31^ | Randomised placebo-controlled trial with a 2x2 factorial design [COMMIT (ClOpidogrel and Metoprolol in Myocardial Infarction Trial; also, Second Chinese Cardiac Study [CCS-2]] | Patients admitted to hospitals within 24 hours of suspected acute MI onset | Excluded because it assessed the efficacy and safety of early beta-blocker therapy (intravenous then oral metoprolol) until discharge or up to four weeks in hospital | 45,852  (metoprolol = 22,929; matching placebo = 22,923). | The use of early beta-blocker therapy in acute MI reduces the risks of reinfarction and ventricular fibrillation, but increases the risk of cardiogenic shock, especially during the first day or so after admission. |
| Coiro et al. (2017)^32^ | Post hoc analysis of the Carvedilol on Outcome after Myocardial Infarction in Patients with Left Ventricular Dysfunction trial (CAPRICORN), Eplerenone’s Neurohormonal Efficacy and Survival Study (EPHESUS), the Optimal Trial in Myocardial Infarction with Angiotensin II Antagonist Losartan (OPTIMAAL) and Valsartan in Acute Myocardial Infarction trial (VALIANT). | Patients with chronic obstructive pulmonary disease post-MI with reduced LVEF (≤ 40%), heart failure, or both | Wrong population: The study exclusively reports results for patients with chronic obstructive pulmonary disease, representing a distinct clinical subgroup. As our analysis focuses on the general post–MI population, inclusion of this study could reduce generalizability and bias the pooled estimates | 1,571 (Beta-blocker users = 822; non-users = 751) | Beta-blocker therapy was associated with improved outcomes among patients with chronic obstructive pulmonary disease post-MI. |
| Daga et al. (2003)^33^ | Randomised, double-blind, controlled, prospective study | Patients with acute MI | Excluded because it analysed the clinical and antioxidant effects of esmolol, an ultra-short acting beta-blocker on patients with acute MI. | 30 (esmolol = 15; control = 15) | The authors reported a significant reduction in heart rate and systolic blood pressure at 30 minutes of esmolol infusion (18.7% and 11.4% reduction respectively), implying that the antioxidant action of beta-blockers may decrease the reperfusion injury in MI. |
| Er et al. (2016)^34^ | Prospective, randomised, single-blind trial [The BEAT-AMI (BEtA-Blocker Therapy in Acute Myocardial Infarction)] | Patients with STEMI and successful PCI (Killip class I and II). | Excluded because it evaluated the role of intravenous esmolol-induced tight sympathetic control | 101 (intravenous esmolol = 50; placebo = 51) | Esmolol treatment statistically significantly decreased troponin T, CK, CK-MB and NT-proBNP release as surrogate markers for myocardial injury in patients with STEMI. |
| Hanada et al. (2012)^35^ | Randomised controlled trial | Patients with STEMI undergoing PCI within 12 hours after the onset of AMI were included. | Excluded because it assessed the intravenous administration of an ultra-short-acting beta-blocker, landiolol, in the very acute phase of MI. | 96  (landiolol = 47; control = 49) | Early intravenous administration of landiolol in patients with MI undergoing PCI is safe and has the potential to improve cardiac function and inhibit cardiac remodelling in the chronic phase. |
| Ibanez et al. (2013)^36^ | Randomised multicenter, parallel-group, single-blinded (to outcome evaluators) controlled trial [METOCARD-CNIC (The Effect of Metoprolol in Cardioprotection During an Acute Myocardial Infarction)] | Patients with Killip class II or less anterior STEMI undergoing PCI within 6 hours of symptoms onset | Excluded because it assessed the effects of early intravenous metoprolol before reperfusion on infarct size. | 270 (intravenous metoprolol pre-reperfusion = 139; no metoprolol pre-reperfusion = 131) | In patients with anterior Killip class II or less STEMI undergoing primary PCI, early intravenous metoprolol before reperfusion reduced infarct size and increased left ventricular ejection fraction with no excess of adverse events during the first 24 hours after STEMI. |
| Janosi et al. (2003)^37^ | Post hoc analysis of the Metoprolol CR/XL Randomized Intervention Trial in Heart Failure (MERIT-HF) trial | Patients with symptomatic chronic heart failure in New York Heart Association (NYHA) class II to IV and LVEF ≤ 40% | Wrong population: The trial was conducted between February 14, 1997, and October 31, 1998, which does not reflect our inclusion criteria for the reperfusion era. | 3,991 (metoprolol CR/XL = 1,990; placebo = 2,001) | In patients with symptomatic heart failure, metoprolol CR/XL improved survival, reduced heart-failure–related hospitalizations, improved NYHA class, and enhanced patient well-being. |
| Matsumoto et al. (2025)^38^ | Post hoc analysis of the Irbesartan in Heart Failure with Preserved Ejection Fraction Study (I-Preserve), the Treatment of Preserved Cardiac Function Heart Failure with an Aldosterone Antagonist Trial (TOPCAT), the Prospective Comparison of ARNI [angiotensin receptor–neprilysin inhibitor] with ARB [angiotensin receptor blocker] Global Outcomes in Heart Failure with Preserved Ejection Fraction trial (PARAGON-HF), and the Dapagliflozin Evaluation to Improve the Lives of Patients with Preserved Ejection Fraction Heart Failure trial (DELIVER) | Patients with heart failure and mildly or preserved LVEF (≥40%) | Wrong population: The sample included patients without previous MI which is our population of interest. | 16,951 (Beta-blocker users = 12,810; non-users = 4,141) | Beta-blocker therapy was not associated with worse heart failure outcomes in patients with mildly reduced or preserved ejection fraction, even after comprehensive adjustment for relevant prognostic factors. |
| Mitchell et al. (2002)^39^ | Multicenter prospective randomised controlled trial | Patients with acute coronary syndromes and relative contraindication to beta-blocker therapy | Excluded because it evaluated the role of intravenous esmolol at six weeks | 108  (intravenous esmolol = 55; standard therapy = 52) | The use of an ultra-short-acting beta-blocker such as esmolol might offer an alternative to patients with contraindications to standard beta-blocker therapy |
| Roolvink et al. (2016)^40^ | Double-blind, placebo-controlled international multicenter study [EARLY-BAMI (Early-Beta blocker Administration before reperfusion primary PCI in patients with ST-elevation Myocardial Infarction)] | STEMI patients presenting <12 h from symptom onset in Killip class I to II without atrioventricular block | Excluded because it assessed the effects of intravenous metoprolol before reperfusion on infarct size at 30 days. | 683 (metoprolol = 336; placebo = 346) | In a nonrestricted STEMI population, early intravenous metoprolol before PCI was not associated with a reduction in infarct size. |
| Shirotani et al. (2010)^41^ | Single-center, not strictly randomised prospective controlled clinical trial with parallel groups | Post-MI patients who underwent emergency PCI for an infarct-related artery showing a Thrombolysis in Myocardial Infarction trial flow grade of 0 or 1. | Excluded due to short term follow-up (four weeks) | 69 (oral atenolol = 35; control = 34) | This study showed that atenolol 50 mg/day did not increase coronary spasm in Japanese acute MI patients. It is suggested that beta-blockers can be safely used soon after coronary intervention for acute MI without the risk of increasing coronary spasm. |
| Tereshchenko et al. (2005)^42^ | Single-center randomised controlled trial | Patients with acute Q-wave MI and Killip class I-II heart failure | Excluded due to short term follow-up (30 days) | 40 (esmolol = 22; standard therapy = 18) | Esmolol treated patients had significantly lower in-hospital mortality, less frequently had postinfarction angina, and heart failure progression. |
| van Veldhuisen et al. (2009)^43^ | Post hoc analysis of the SENIORS Trial (Study of Effects of Nebivolol Intervention on Outcomes and Rehospitalization in Seniors With Heart Failure) | Elderly patients (≥70 years) with a clinical history of heart failure. | Wrong population: The sample included patients without previous MI which is our population of interest. | 2111 (Nebivolol = 1058; Placebo = 1053) | In elderly heart failure patients, nebivolol demonstrated a similar effect irrespective of LVEF, with comparable outcomes for all-cause mortality and cardiovascular hospitalizations. |
| **Author (year)** | **Type of study** | **Population** | **Reason for exclusion** | **Total sample size** | **Results** |
| **Observational studies** | | | | | |
| Emery et al. (2006)^44^ | Retrospective observational cohort study | Patients ≥18 years with confirmed NSTEMI | **Wrong intervention and follow-up:** The study evaluated early beta-blocker use (within 24 hours of the event), which may have included intravenous administration, and assessed outcomes only at 6 months, both of which did not meet our inclusion criteria. | 7,106 (early beta-blocker = 5,422; non-user = 1,684) | Early beta-blocker therapy was independently correlated with a lower mortality for all NSTEMI. There were fewer fatal and nonfatal arrhythmias and a lower incidence of cardiogenic shock. |
| Goldberger et al. (2015)^45^ | Retrospective observational cohort study | Post-MI patients enrolled in the OBTAIN registry | **Wrong intervention:** the study compared different doses of beta-blocker therapy. | 6,682 | All beta-blocker doses were associated with lower mortality versus no beta-blocker; however, no consistent survival benefit observed for higher (>50%) vs. lower doses. |
| Kernis et al. (2004)^46^ | Retrospective observational cohort study | Patients with MI who underwent PCI | **Wrong follow-up duration:** the study assessed outcomes at 6 months, which did not meet our inclusion criteria for long-term follow-up. | 2,442  (beta-blocker users = 1,661; non-users = 781) | Treatment with beta-blockers after successful primary PCI is associated with reduced six-month mortality, with the greatest benefit in patients with a low ejection fraction or multi-vessel coronary artery disease. |
| Kim et al. (2020)^47^ | Retrospective observational cohort study | Patients ≥18 years who underwent revascularization for MI without heart failure. | **Wrong comparison:** the study compared outcomes between individuals who received beta-blocker therapy for more than one year versus those who received it for less than one year, rather than comparing users to non-users. | 28,970 | Beta-blocker therapy for more than 1 year after MI was associated with reduced mortality. |
| Konishi et al. (2011)^48^ | Retrospective single-center observational cohort study | Post-MI patients who underwent primary coronary revascularization | **Wrong intervention:** The study assessed the additive effects of beta-blocker therapy on renin-angiotensin system inhibitor treatment in patients after AMI. | 251 (beta-blocker users = 171; non-users = 80) | For post-MI patients, combination therapy with renin–angiotensin system inhibitors and beta-blocker therapy is more effective than renin–angiotensin system inhibitors alone. |
| LaFon et al. (2024)^49^ | Prospective multicenter observational study | Chronic obstructive pulmonary disease patients post-MI | **Wrong population**: The study exclusively enrolled patients with chronic obstructive pulmonary disease, representing a distinct clinical subgroup. As our analysis focuses on the general post–MI population, inclusion of this study could reduce generalizability and bias the pooled estimates | 579 (beta-blocker users = 502; non-users = 77) | Beta-blocker therapy at hospital discharge was not associated with increased risk of all-cause mortality, hospitalizations, or revascularizations in patients with chronic obstructive pulmonary disease post-MI. |
| Padilla López et al. (2020)^50^ | Retrospective observational cohort study | Patients ≥18 years with first STEMI type 1 | **Wrong intervention:** the study aimed to establish the first prescription and its dispensation in the first 30 days of the four pharmacotherapeutic classes and did not assess beta-blocker therapy alone. | 576 | Not receiving the treatment due to lack of complete prescription after discharge or lack of complete primary adherence in patients who have type 1 STEMI can condition a higher 1-year cardiovascular mortality. |
| Thattassery and Gheorghiade (2004)^51^ | Narrative review | Post-MI patients with left ventricular systolic dysfunction and heart failure | **Wong study type:** narrative review | - | Patients with heart failure or systolic dysfunction after a MI should and without contraindications should be started on carvedilol prior to discharge. Patients should be gradually titrated to the recommended dose and continued indefinitely. |

STEMI = ST- Elevation Myocardial Infarction; MI = Myocardial Infarction; PCI = Percutaneous Coronary Intervention; NSTEMI = Non-ST- Elevation Myocardial Infarction; CK = Creatine Kinase; NT-proBNP = N-terminal pro-B-type natriuretic peptide.

**Supplemental Figure 1: Stratified Forest plot of observational studies for all-cause mortality.**

Caption: Forest plots of observational studies showing hazard ratios (HRs) for all-cause mortality in post–MI patients treated with beta-blocker therapy. The pooled random-effects estimates indicate a significant reduction in all-cause mortality in PSM studies (HR 0·71, 95% CI 0·61–0·84) and in non-PSM studies (HR 0·79, 95% CI 0·69–0·89), although substantial between-study heterogeneity was present (I² = 81·4% and 91·4%, respectively).


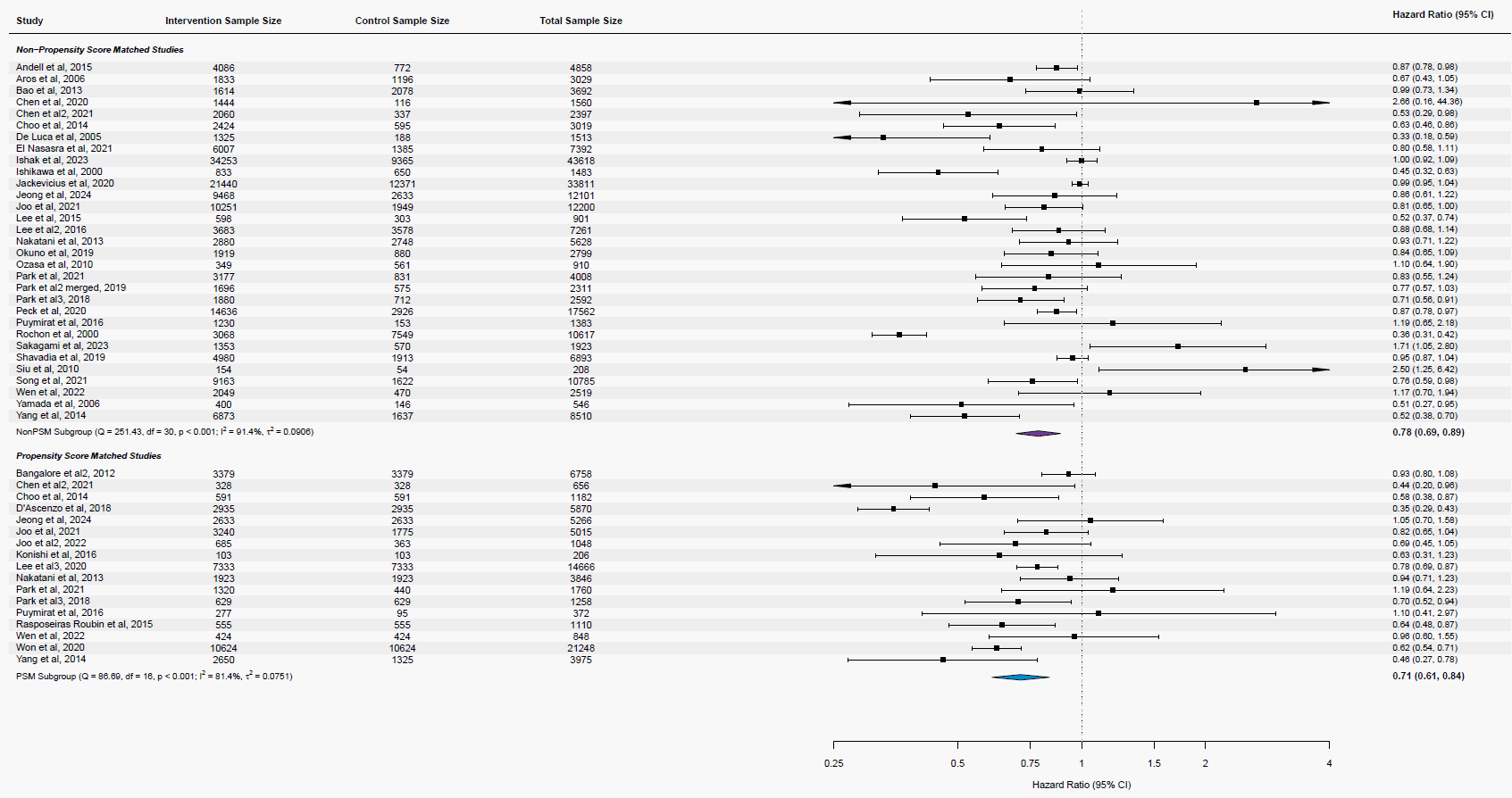


**Supplemental Figure 2: Stratified Forest plot of observational studies for cardiovascular mortality.**

Caption: Forest plots of observational studies showing hazard ratios (HRs) for cardiovascular mortality in post–MI patients treated with beta-blocker therapy. Beta-blocker therapy was associated with a significant reduction in cardiovascular mortality in PSM studies (HR 0·72, 95% CI 0·57–0·92) and in non-PSM studies (HR 0·80, 95% CI 0·65–0·98), with moderate to substantial heterogeneity across analyses (I² = 57·8% and 78·9%, respectively).


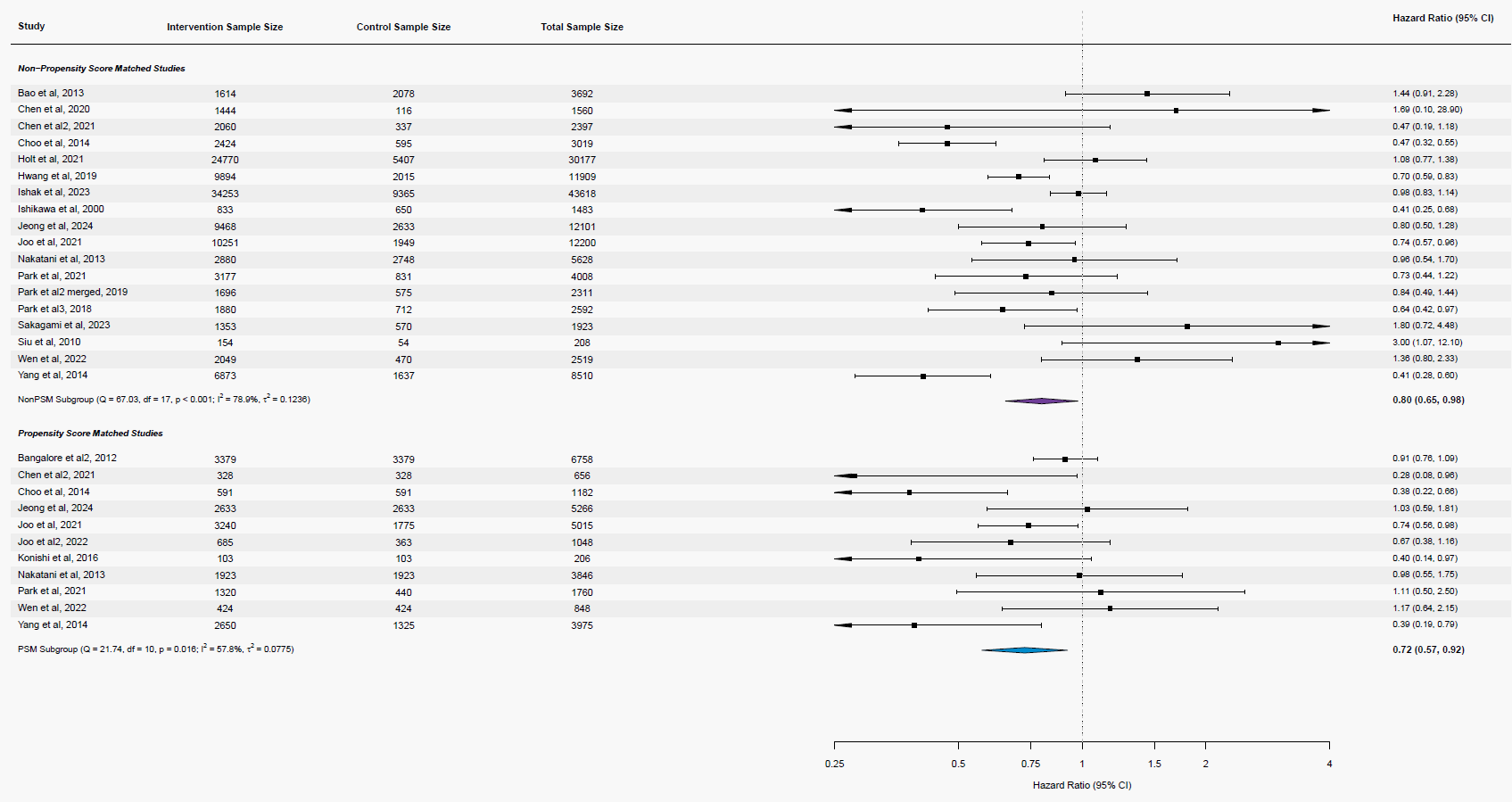


**Supplemental Figure 3: Stratified Forest plot of observational studies for recurrent MI.**

Caption: Forest plots of observational studies showing hazard ratios (HRs) for recurrent MI in post–MI patients treated with beta-blocker therapy. PSM studies suggested a non-significant reduction in recurrent MI (HR 0·83, 95% CI 0·67–1·02; I² = 39·4%), whereas non-PSM studies showed no evidence of benefit (HR 1·01, 95% CI 0·94–1·08) with no observed heterogeneity (I² = 0%).


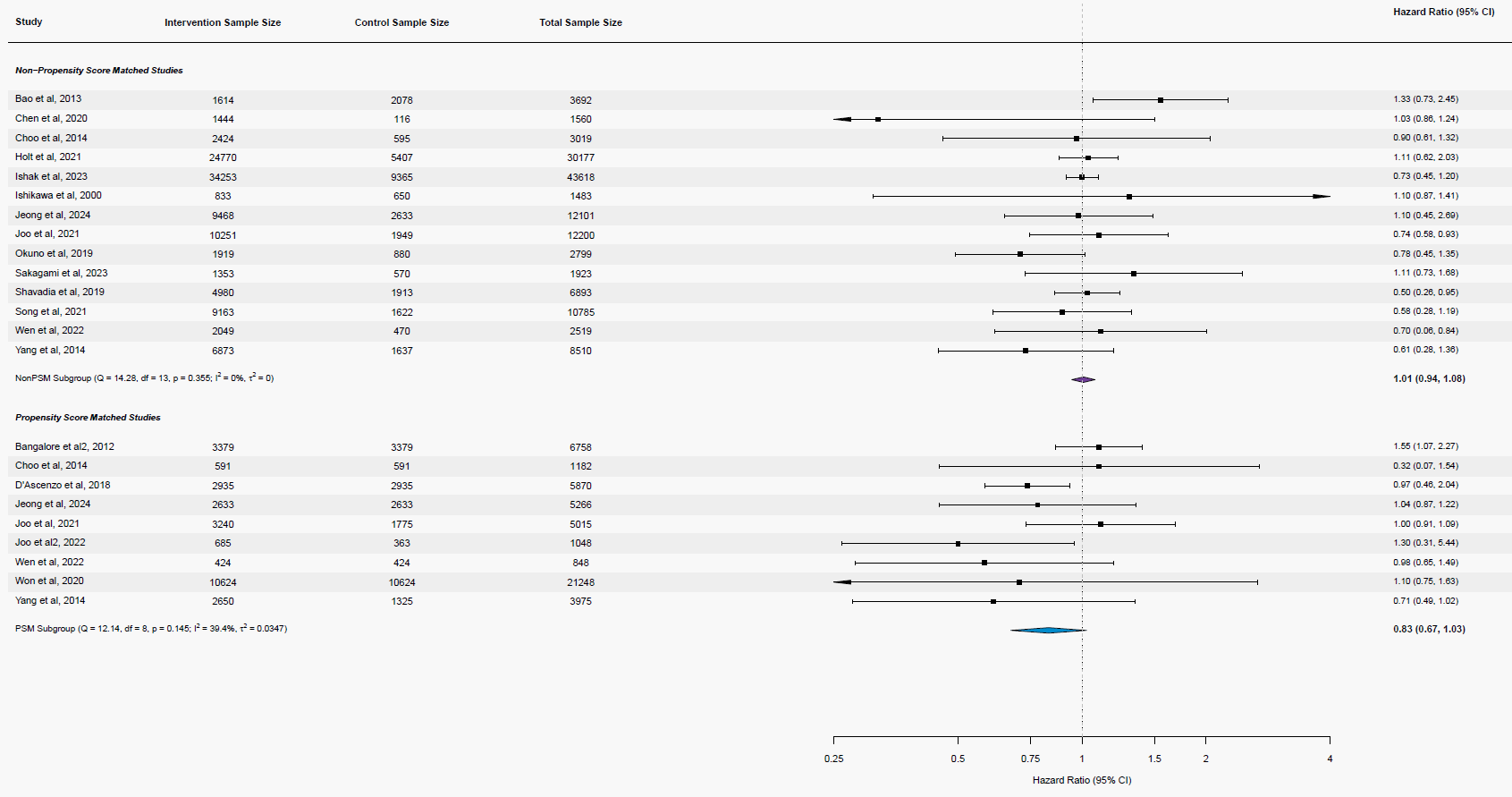


**Supplemental Table 6: Sample sizes used in stratified meta-analyses.**

| **Study Type** | **Total Participants** | **Beta-blocker users n (%)** | **Non-users n (%)** |
| --- | --- | --- | --- |
| **All-cause mortality*** |  |  |  |
| RCTs (n=7) | 24,191 | 12,099 (50%) | 12,092 (50%) |
| PSM (n=18) | 76,038 | 40,106 (52·7%) | 35,932 (47·3%) |
| Non-PSM (n=32) | 236,428 | 172,667 (73%) | 63,661 (27%) |
| Total (n=57) | 336,657 | 224,872 (66·8%) | 111,685 (33·2%) |
| **Cardiovascular mortality†** |  |  |  |
| RCTs (n=5) | 18,173 | 9,065 (49·9%) | 9,108 (50·1%) |
| PSM (n=11) | 30,560 | 17,276 (56·5%) | 13,284 (43·5%) |
| Non-PSM (n=18) | 149,855 | 117,073 (78·1%) | 32,742 (21·8%) |
| Total (n=34) | 198,588 | 143,414 (72·2%) | 55,134 (27·8%) |
| **Recurrent MI†** |  |  |  |
| RCTs (n=6) | 23,747 | 11,848 (49·9%) | 11,899 (50·1%) |
| PSM (n=9) | 51,210 | 27,161 (53%) | 24,049 (47%) |
| Non-PSM (n=14) | 141,279 | 111,394 (78·8%) | 29,885 (21·2%) |
| Total (n=29) | 216,236 | 150,403 (69·6%) | 65,833 (30·4%) |

RCTs = Randomised Controlled Trials; PSM = Propensity Score Matching; Non-PSM = Non-Propensity Score Matching.

*Studies excluded: Amano et al. (duplication of results), Silvain et al. (composite outcome), Dondo et al. (could not be converted due to lack of event rate data) and McMurray et al. (outcome reported not of interest).

**^†§^**Studies excluded: Amano et al. (duplication of results) and McMurray et al. (outcome reported not of interest).

**Supplemental Figure 4: Meta-regression of follow-up duration and beta-blocker therapy treatment effects in non-PSM studies.**

Caption: Meta-regression plots of non-PSM studies assessing the association between follow-up duration and beta-blocker treatment effects on all-cause mortality, cardiovascular mortality, and recurrent MI. Longer follow-up duration was significantly associated with modest attenuation of treatment effects for all-cause mortality (slope +0·09 per year, *p* = 0·005) and cardiovascular mortality (slope +0·18 per year, *p* = 0·03), explaining 6·7% and 18·2% of between-study heterogeneity, respectively. No significant association was observed for recurrent MI (slope +0·005 per year, *p* = 0·80).


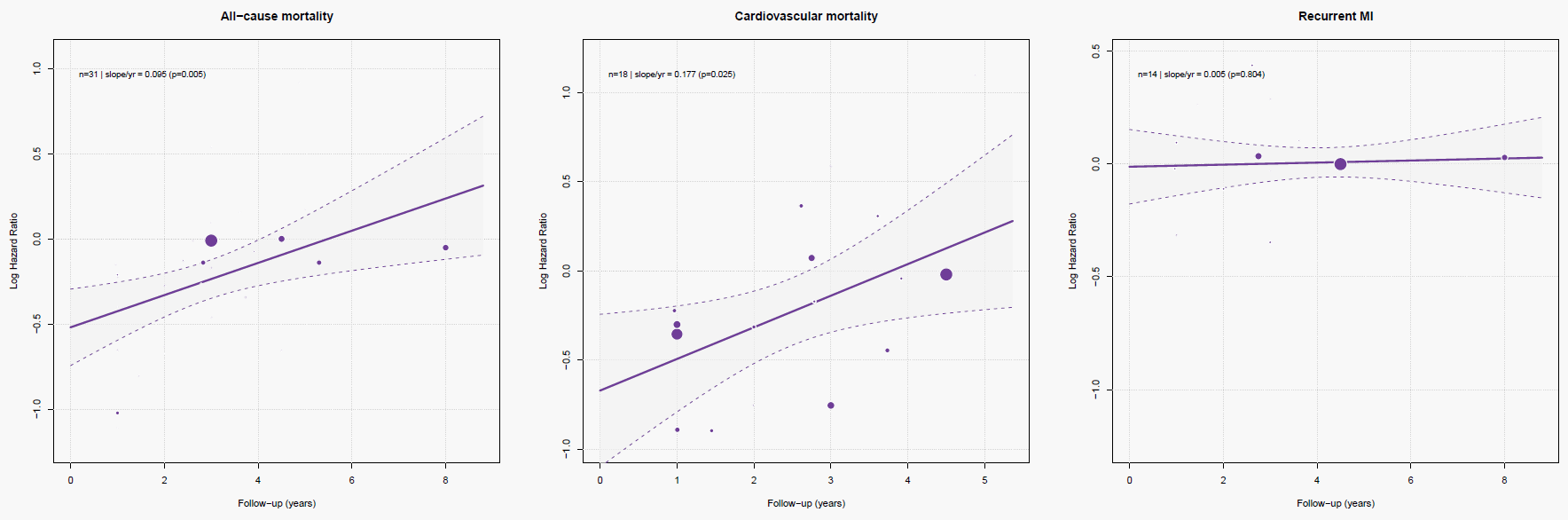


**Supplemental Figure 5: Meta-regression of follow-up duration and beta-blocker therapy treatment effects in PSM studies.**

Caption: Meta-regression plots of PSM studies examining the relationship between follow-up duration and beta-blocker treatment effects for all-cause mortality, cardiovascular mortality, and recurrent myocardial infarction. No significant associations were observed between follow-up duration and treatment effects for any outcome (all p > 0·05), with minimal heterogeneity explained (R² ≤ 22·4%).


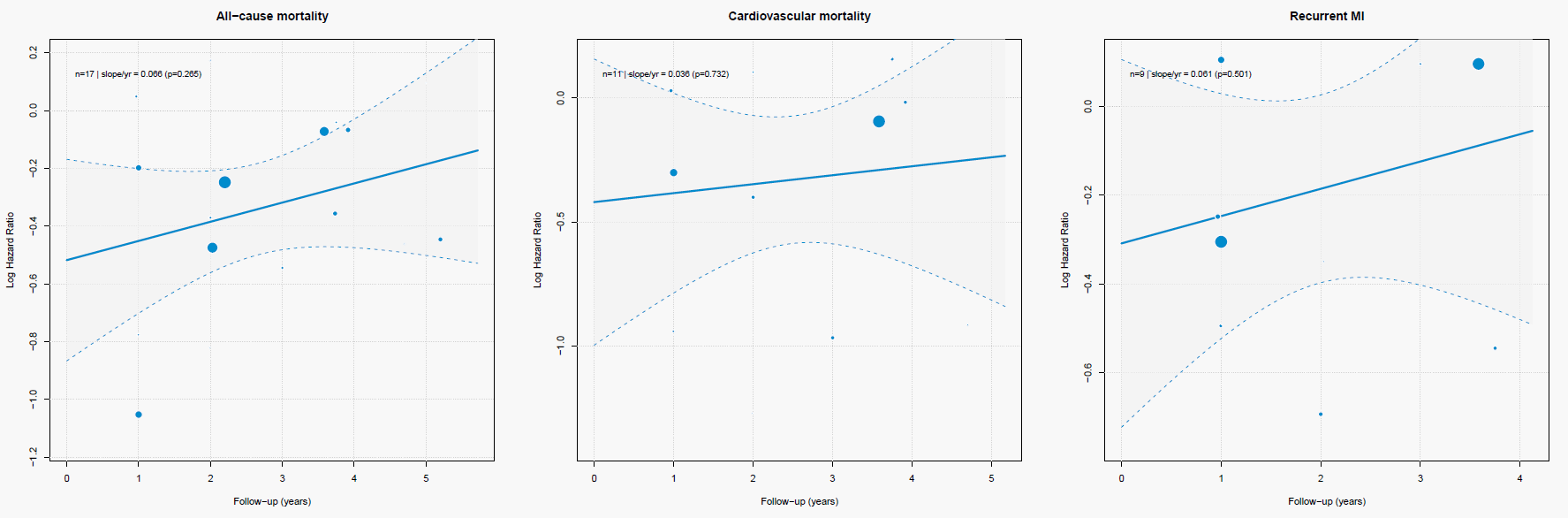


**Supplemental Table 7: Associations of beta-blocker therapy with all-cause mortality, cardiovascular mortality, and recurrent MI in observational studies, stratified by LVEF subgroup.**

| **Study Design** | **LVEF Subgroup** | **Outcome** | **HR (95% CI)** | **Number. of studies** | **I² (%)** |
| --- | --- | --- | --- | --- | --- |
| PSM | Preserved (≥50%) | All-cause mortality | 0·71 (0·53–0·94) | 11 | 81·3 |
|  |  | Cardiovascular mortality | 0·70 (0·44–1·10) | 8 | 67·4 |
|  |  | Recurrent MI | 0·85 (0·63–1·13) | 6 | 50·2 |
|  | Midrange (40–49%) | All-cause mortality | 0·70 (0·50–0·98) | 2 | 0 |
|  |  | Cardiovascular mortality | 0·64 (0·42–0·98) | 2 | 0 |
|  |  | Recurrent MI | 0·43 (0·26–0·72) | 2 | 0 |
| Non-PSM | Preserved (≥50%) | All-cause mortality | 0·88 (0·74–1·04) | 20 | 80 |
|  |  | Cardiovascular mortality | 0·86 (0·67–1·11) | 15 | 80·6 |
|  |  | Recurrent MI | 1·00 (0·92–1·09) | 10 | 0 |
|  | Midrange (40–49%) | All-cause mortality | 0·63 (0·44–0·91) | 4 | 54 |
|  |  | Cardiovascular mortality | N/A | N/A | N/A |
|  |  | Recurrent MI | 0·44 (0·29–0·68) | 2 | 0 |

PSM = Propensity Score Matching; Non-PSM = Non-Propensity Score Matching; LVEF = Left Ventricular Ejection Fraction; MI = Myocardial Infarction; HR = Hazard Ratio; CI = Confidence Interval.

**Supplemental Table 8: Quality of evidence assessment using the JBI tool.**

| **Question / Systematic review** | 1. Is the review question clearly and explicitly stated? | 2. Were the inclusion criteria appropriate for the review question? | 3. Was the search strategy appropriate? | 4. Were the sources and resources used to search for studies adequate? | 5. Were the criteria for appraising studies appropriate? | 6. Was critical appraisal conducted by two or more reviewers independently? | 7. Were there methods to minimise errors in data extraction? | 8. Were the methods used to combine studies appropriate? | 9. Was the likelihood of publication bias assessed? | 10. Were recommendations for policy and/or practice supported by the reported data? | 11. Were the specific directives for new research appropriate? | Overall quality rating | |
| --- | --- | --- | --- | --- | --- | --- | --- | --- | --- | --- | --- | --- | --- |
| **Affas et al.  (2025)**^52^ | Yes | Yes | Unclear | No | Yes | Yes | Yes | Yes | No | No | Yes | 7 / 11 | Moderate quality |
| **Alnemer et al. (2025)**^53^ | Yes | Yes | Unclear | No | Yes | No | Unclear | Yes | Yes | Yes | Yes | 7 / 11 | Moderate quality |
| **Chen et al. (2025)**^54^ | Yes | Yes | Unclear | Yes | Yes | Yes | Yes | Yes | No | Yes | Yes | 9 / 11 | High quality |
| **Chi et al. (2024)**^55^ | Yes | Yes | Yes | Yes | Yes | Yes | Yes | Yes | Yes | Yes | Yes | 11 / 11 | High quality |
| **Dahl Aarvik  et al. (2019)**^56^ | Yes | Yes | Yes | Yes | Yes | Unclear | Unclear | Yes | Yes | Yes | Yes | 9 / 11 | High quality |
| **Gomes et al. (2025)**^57^ | Yes | No | No | No | Yes | Unclear | Yes | Yes | No | No | No | 4 / 11 | Low quality |
| **(1) Hu et al. (2022)**^58^ | Yes | Yes | Yes | Yes | No | N/A | Unclear | Yes | Yes | Yes | No | 7 / 11 | Moderate quality |
| **(2) Hu et al. (2022)**^59^ | Yes | Yes | Yes | Yes | No | N/A | Yes | Yes | Yes | Yes | Yes | 9 / 11 | High quality |
| **Kim et al. (2022)**^60^ | Yes | Yes | Yes | No | Yes | Unclear | Unclear | Yes | No | Yes | Yes | 7 / 11 | Moderate quality |
| **Kristensen et al. (2025)**^61^ | Yes | Yes | Yes | No | Yes | Unclear | Unclear | Yes | No | Yes | Yes | 7 / 11 | Moderate quality |
| **Liang et al. (2022)**^62^ | Yes | Yes | Yes | Yes | Yes | Unclear | Unclear | Yes | Yes | Yes | Yes | 9 / 11 | High quality |
| **Maqsood et al. (2021)**^63^ | Yes | Yes | No | No | Yes | Yes | Yes | Yes | Yes | Yes | Yes | 9 / 11 | High quality |
| **Rossello et al. (2025)**^64^ | Yes | Yes | Yes | No | N/A | N/A | Yes | Yes | No | Yes | Yes | 7 / 11 | Moderate quality |
| **Sabina et al. (2025)**^24^ | Yes | Yes | Yes | No | Yes | Yes | Yes | Yes | No | Yes | Yes | 9 / 11 | High quality |
| **Safi et al. (2019)**^65^ | Yes | Yes | Yes | Yes | Yes | Yes | Yes | Yes | Yes | Yes | Yes | 11 / 11 | High quality |
| **Safi et al. (2021)**^66^ | Yes | Yes | Yes | Yes | Yes | Yes | Yes | Yes | Yes | Yes | Yes | 11 / 11 | High quality |
| **Sidiq et al. (2025)**^67^ | Yes | Yes | Unclear | No | N/A | N/A | Yes | Yes | No | Yes | Yes | 6 / 11 | Moderate quality |
| **Singh et al. (2024)**^68^ | Yes | Yes | Yes | Yes | Unclear | Yes | Yes | Yes | No | Yes | Yes | 9 / 11 | High quality |
| **Yang et al. (2025)**^69^ | Yes | No | Yes | Yes | N/A | N/A | Yes | No | Yes | No | No | 5 / 11 | Low quality |

**Supplemental Table 9: Domain-level risk of bias assessment of included randomised controlled trials using the Cochrane Risk of Bias 2 (RoB 2) tool**

| **Study** | **Randomisation** | **Deviations** | **Missing Data** | **Outcome Measurement** | **Selective Reporting** | **Overall** |
| --- | --- | --- | --- | --- | --- | --- |
| Dargie et al. (2001)^70^ | 🟢 | 🟢 | 🟢 | 🟢 | 🟢 | 🟢 |
| Ibanez et al. (2025)^71^ | 🟢 | 🟡 | 🟢 | 🟢 | 🟢 | 🟡 |
| Munkhaugen et al. (2025)^72^ | 🟢 | 🟡 | 🟢 | 🟢 | 🟢 | 🟡 |
| Silvain et al. (2024)^73^ | 🟢 | 🟡 | 🟢 | 🟡 | 🟢 | 🟡 |
| Watanabe et al. (2018)^74^ | 🟢 | 🟡 | 🟢 | 🟡 | 🟡 | 🟡 |
| Yndigegn et al. (2024)^75^ | 🟢 | 🟡 | 🟢 | 🟢 | 🟢 | 🟡 |

Traffic light symbols indicate risk of bias judgements for each domain and overall study assessment:
🟢 Low risk of bias 🟡 Some concerns 🔴 High risk of bias

**Supplemental Table 10: Domain-level risk of bias assessment of included non-randomised studies using the Risk Of Bias In Non-randomised Studies of Interventions (ROBINS-I) tool.**

| **Study** | **Confounding** | **Selection** | **Classification** | **Missing Data** | **Outcome** | **Reporting** | **Overall** |
| --- | --- | --- | --- | --- | --- | --- | --- |
| Al-Bawardy et al. (2024)^76^ | 🟡 | 🟡 | 🟡 | 🟡 | 🟢 | 🟡 | 🟡 |
| Amano et al. (2023)^77^ | 🟠 | 🟡 | 🟡 | 🟢 | 🟢 | 🟠 | 🟠 |
| Andell et al. (2015)^78^ | 🟠 | 🟡 | 🟡 | 🟢 | 🟢 | 🟡 | 🟠 |
| Arós et al. (2006)^79^ | 🟠 | 🟡 | 🟡 | 🟡 | 🟢 | 🟡 | 🟠 |
| Bangalore et al. (2012)^80^ | 🟡 | 🟡 | 🟡 | 🟡 | 🟢 | 🟡 | 🟠 |
| Bangalore et al. (2014)^81^ | 🟢 | 🟡 | 🟡 | 🟡 | 🟢 | 🟠 | 🟠 |
| Bao et al. (2013)^82^ | 🟡 | 🟡 | 🟡 | 🟡 | 🟢 | 🟠 | 🟠 |
| Chen et al. (2020)^83^ | 🟠 | 🟡 | 🟡 | 🟢 | 🟢 | 🟡 | 🟠 |
| Chen et al. (2021)^84^ | 🟡 | 🟡 | 🟡 | 🟢 | 🟢 | 🟡 | 🟡 |
| Choo et al. (2014)^85^ | 🟡 | 🟡 | 🟡 | 🟡 | 🟢 | 🟡 | 🟡 |
| D’Ascenzo et al. (2018)^86^ | 🟡 | 🟡 | 🟡 | 🟠 | 🟢 | 🟡 | 🟠 |
| De Luca et al. (2005)^87^ | 🟠 | 🟡 | 🟡 | 🟡 | 🟢 | 🟡 | 🟠 |
| Dondo et al. (2017)^88^ | 🟠 | 🟡 | 🟡 | 🟡 | 🟢 | 🟡 | 🟠 |
| El Nasasra et al. (2020)^89^ | 🟠 | 🟡 | 🟡 | 🟡 | 🟢 | 🟡 | 🟠 |
| Hioki et al. (2016)^90^ | 🟠 | 🟡 | 🟡 | 🟡 | 🟢 | 🟠 | 🟠 |
| Holt et al. (2021)^91^ | 🟠 | 🟡 | 🟡 | 🟢 | 🟢 | 🟡 | 🟠 |
| Hwang et al. (2019)^92^ | 🟠 | 🟡 | 🟡 | 🟡 | 🟢 | 🟡 | 🟠 |
| Ishak et al. (2023)^93^ | 🟡 | 🟡 | 🟡 | 🟢 | 🟢 | 🟢 | 🟡 |
| Ishikawa et al. (2000)^94^ | 🔴 | 🟠 | 🟡 | 🟡 | 🟡 | 🟠 | 🟠 |
| Jackevicius et al. (2020)^95^ | 🟠 | 🟡 | 🟡 | 🟢 | 🟢 | 🟡 | 🟠 |
| Jeong et al. (2024)^96^ | 🟡 | 🟡 | 🟡 | 🟢 | 🟢 | 🟡 | 🟡 |
| Joo et al. (2021)^97^ | 🟡 | 🟡 | 🟡 | 🟡 | 🟢 | 🟡 | 🟡 |
| Joo et al. (2022)^98^ | 🟡 | 🟡 | 🟡 | 🟠 | 🟢 | 🟡 | 🟠 |
| Konishi et al. (2016)^99^ | 🟡 | 🟡 | 🟡 | 🟠 | 🟢 | 🟡 | 🟠 |
| Lee et al. (2015)^100^ | 🟠 | 🟡 | 🟡 | 🟡 | 🟡 | 🟠 | 🟠 |
| Lee et al. (2016)^101^ | 🟠 | 🟡 | 🟡 | 🟡 | 🟢 | 🟡 | 🟠 |
| Lee et al. (2020)^102^ | 🟡 | 🟡 | 🟡 | 🟡 | 🟢 | 🟢 | 🟡 |
| McMurray et al. (2005)^103^ | 🟢 | 🟢 | 🟢 | 🟢 | 🟡 | 🟠 | 🟠 |
| Nakatani et al. (2013)^104^ | 🟡 | 🟡 | 🟡 | 🟡 | 🟢 | 🟡 | 🟡 |
| Okuno et al. (2019)^105^ | 🟠 | 🟡 | 🟡 | 🟠 | 🟢 | 🟡 | 🟠 |
| Ozasa et al. (2010)^106^ | 🟠 | 🟡 | 🟡 | 🟡 | 🟢 | 🟡 | 🟠 |
| Park et al. (2018)^107^ | 🟡 | 🟠 | 🟡 | 🟡 | 🟢 | 🟡 | 🟠 |
| Park et al. (2019)^108^ | 🟠 | 🟡 | 🟡 | 🟢 | 🟢 | 🟡 | 🟠 |
| Park et al. (2021)^109^ | 🟡 | 🟠 | 🟡 | 🟡 | 🟢 | 🟡 | 🟠 |
| Peck et al. (2020)^110^ | 🟠 | 🟡 | 🟡 | 🟢 | 🟢 | 🟡 | 🟠 |
| Puymirat et al. (2016)^111^ | 🟡 | 🟡 | 🟡 | 🟠 | 🟢 | 🟡 | 🟠 |
| Raposeiras-Roubin et al. (2015)^112^ | 🟡 | 🟡 | 🟡 | 🟠 | 🟢 | 🟡 | 🟠 |
| Rochon et al. (2000)^113^ | 🟠 | 🟡 | 🟡 | 🟢 | 🟢 | 🟡 | 🟠 |
| Sakagami et al. (2023)^114^ | 🟠 | 🟡 | 🟡 | 🟡 | 🟢 | 🟡 | 🟠 |
| Shavadia et al. (2019)^115^ | 🟠 | 🟠 | 🟡 | 🟢 | 🟢 | 🟡 | 🟠 |
| Siu et al. (2010)^116^ | 🔴 | 🟡 | 🟡 | 🟠 | 🟢 | 🟡 | 🟠 |
| Song et al. (2019)^117^ | 🟠 | 🟡 | 🟡 | 🟡 | 🟢 | 🟡 | 🟠 |
| Wen et al. (2022)^118^ | 🟡 | 🟡 | 🟡 | 🟡 | 🟢 | 🟡 | 🟡 |
| Won et al. (2020)^119^ | 🟡 | 🟡 | 🟡 | 🟠 | 🟢 | 🟡 | 🟠 |
| Yamada et al. (2006)^120^ | 🟠 | 🟡 | 🟡 | 🟠 | 🟢 | 🟡 | 🟠 |
| Yang et al. (2014)^69^ | 🟡 | 🟡 | 🟡 | 🟡 | 🟢 | 🟡 | 🟡 |

Traffic light symbols indicate risk of bias judgements for each domain and overall study assessment:
🟢 Low risk of bias 🟡 Moderate risk of bias 🟠 Serious risk of bias 🔴 Critical risk of bias

**Supplemental Table 11: Certainty of evidence for all-cause mortality.**

| **Systematic review** | **Effect measure** | **N of participants** | **Certainty of evidence** | **Comments** |
| --- | --- | --- | --- | --- |
| **Intervention / comparison** | **(95% CI)** | **(studies)** | **(GRADE)** |  |
| **Affas et al. (2025)**^52^ | RR 0·6 | 18,459 | Very Low | Moderate risk of bias and imprecision. Serious inconsistency and publication bias not assessed. |
| Use of beta-blockers after hospital discharge / non-use of beta blockers | (0·43 - 0·85) | (4 observational and 2 RCTs) |  |  |
| **Alnemer et al. (2025)**^53^ | HR 0·78 | 23,706 | Low | Moderate risk of bias and inconsistency as most included studies are observational. Serious publication bias |
| Use of beta-blockers / non-use of beta blockers | (0·67 - 0·91) | (9 observational and 2 RCTs) |  |  |
| **Chen et al. (2025)**^54^ | N / A | 19,113 | Very Low | Moderate risk of bias and imprecision. Serious inconsistency and publication bias not reported. |
| Use of beta-blockers / non-use of beta blockers |  | (5 observational and 2 RCTs) |  |  |
| **Chi et al. (2024)**^55^ | HR 0·89 | 332,083 | Low | Moderate risk of bias and inconsistency as most included studies are observational. Serious publication bias |
| Use of beta-blockers / non-use of beta blockers | (0·81 - 0·97) | (19 observational and 3 RCT) |  |  |
| **Dahl Aarvick et al. (2019)**^56^ | RR 0·74 | 189,385 | Low | Moderate risk of bias and serious inconsistency as all included studies are observational. Serious publication bias. |
| Use of beta-blockers / non-use of beta blockers | (0·64 - 0·85) | (16 observational) |  |  |
| **Gomes et al. (2025)**^57^ | HR 0·81 | 88,268 | Low | Moderate risk of bias and serious inconsistency and imprecision. Publication bias not assessed. |
| Use of beta-blockers / non-use of beta blockers or placebo | (0·67 - 0·98) | (9 observational and 1 RCTs) |  |  |
| **(1) Hu et al. (2022)**^58^ | OR 0·7 | 31,907 | Moderate | Moderate risk of bias (Although only PSM studies were included to minimise confounding, a quality assessment of each primary study was not done) |
| Use of beta-blockers after hospital discharge / non-use of beta blockers | (0·61 - 0·8) | (6 observational and 1 RCT) |  |  |
| **(2) Hu et al. (2022)**^59^ | OR 0·66 | 175,160 | Moderate | Moderate risk of bias and serious inconsistency as all included studies are observational. |
| Use of beta-blockers / non-use of beta blockers | (0·5 - 0·86) | (11 observational and 1 RCT) |  |  |
| **Kim et al. (2022)**^60^ | OR 0·8 | 217,532 | Very Low | Moderate risk of bias and imprecision. Serious inconsistency and publication bias not assessed. |
| Beta-blocker prescription continued for 1 year after hospital discharge / No prescription for 1 year after hospital discharge | (0·56 - 1·15) | (5 observational) |  |  |
| **Kristensen et al. (2025)**^61^ | HR 1·04 | 17,801 | Moderate | Serious imprecision (wide confidence intervals that include the possibility of no effect) and publication bias not assessed. |
| Use of beta-blockers / non-use of beta blockers | (0·89 - 1·21) | (5 RCTs) |  |  |
| **Liang et al. (2022)**^62^ | HR 0·67 | 219,452 | Moderate | Moderate risk of bias and serious inconsistency as all included studies are observational. |
| Use of beta-blockers / non-use of beta blockers | (0·56 - 0·8) | (24 observational) |  |  |
| **Maqsood et al. (2021)**^63^ | OR 0·64 | 11,939 | Moderate | Moderate risk of bias and imprecision. Serious inconsistency and publication bias not assessed. |
| Use of beta-blockers / non-use of beta blockers | (0·48 - 0·87) | (5 observational) |  |  |
| **Rossello et al. (2025)**^64^ | HR 0·78 | 1,855 | Moderate | Serious imprecision (wide confidence intervals that include the possibility of no effect) and publication bias not assessed. |
| Use of beta-blockers / non-use of beta blockers | (0·55 - 1·11) | (4 RCTs) |  |  |
| **Sabina et al. (2025)**^24^ | RR 0·96 | 9,512 | Low | Serious imprecision (wide confidence intervals that include the possibility of no effect) and publication bias not assessed. |
| Treated with beta-blockers / not treated with beta-blockers | (0·79 - 1·17) | (3 RCT) |  |  |
| **Safi et al. (2019)**^65^ | RR 0·93 | 25,210 | Moderate | Serious risk of bias (as all but one of the included primary studies were rated high risk) and indirectness (the trials predominantly enrolled younger patients and were conducted in the pre-reperfusion era, limiting their relevance to contemporary clinical practice) |
| Use of any type of beta-blocker (including intravenous and oral therapy) / placebo, no intervention or co-intervention | (0·86 - 0·99) * | (26 RCT) |  |  |
| **Safi et al. (2021)**^66^ | RR 0·81 | 22,085 | Low | Serious risk of bias (as all but one of the included primary studies were rated high risk), indirectness (the trials predominantly enrolled younger patients and were conducted in the pre-reperfusion era, limiting their relevance to contemporary clinical practice) and publication bias. |
| Use of any type of beta-blocker (including intravenous and oral therapy) / placebo, no intervention or co-intervention | (0·73 - 0·9) * | (21 RCT) |  |  |
| **Sidiq et al. (2025)**^67^ | RR 0·98 | 19,826 | Moderate | Serious imprecision (wide confidence intervals that include the possibility of no effect) and publication bias not assessed. |
| Use of beta-blockers / non-use of beta blockers | (0·86 - 1·12) | (4 RCTs) |  |  |
| **Singh et al. (2024)**^68^ | OR 0·64 | 23,697 | Low | Moderate risk of bias, inconsistency and imprecision. Serious publication bias |
| Use of beta-blockers / non-use of beta blockers | (0·45 - 0·9) | (6 observational and 2 RCT) |  |  |
| **Yang et al. (2025)**^69^ | OR 0·73 | 233,303 | Low | Moderate risk of bias and publication bias. Serious inconsistency. |
| Use of beta-blockers / non-use of beta blockers | (0·64 - 0·82) | (30 observational and 4 RCTs) |  |  |

*The confidence interval reported was 97.5%

N° = Number; CI = Confidence Interval; GRADE = Grading of Recommendations Assessment, Development, and Evaluation; HR = Hazard Ratio; RCTs = Randomized Controlled Trials; RR = Risk Ratio; OR = Odds Ratio; PSM = Propensity Score Matching.

**Supplemental Table 12: GRADE assessment details for all-cause mortality.**

| **Systematic review** | **Risk of bias** | **Inconsistency** | **Indirectness** | **Imprecision** | **Publication bias** |
| --- | --- | --- | --- | --- | --- |
| Affas et al. (2025)^52^ | Moderate | Serious | Low | Moderate | Serious |
| Alnemer et al. (2025)^53^ | Moderate | Moderate | Low | Low | Low |
| Chen et al. (2025)^54^ | Moderate | Moderate | Low | Serious | Serious |
| Chi et al. (2024)^55^ | Moderate | Moderate | Low | Low | Serious |
| Dahl Aarvick et al. (2019)^56^ | Moderate | Serious | Low | Low | Serious |
| Gomes et al. (2025)^57^ | Moderate | Serious | Low | Serious | Serious |
| (1) Hu et al. (2022)^58^ | Moderate | Low | Low | Low | Low |
| (2) Hu et al. (2022)^59^ | Moderate | Serious | Low | Low | Low |
| Kim et al. (2022)^60^ | Moderate | Serious | Low | Moderate | Serious |
| Kristensen et al. (2025)^61^ | Low | Low | Low | Serious | Serious |
| Liang et al. (2022)^62^ | Moderate | Serious | Low | Low | Low |
| Maqsood et al. (2021)^63^ | Moderate | Serious | Low | Moderate | Low |
| Rossello et al. (2025)^64^ | Low | Low | Low | Serious | Serious |
| Sabina et al. (2025)^24^ | Low | Low | Low | Serious | Serious |
| Safi et al. (2019)^65^ | Serious | Low | Serious | Low | Low |
| Safi et al. (2021)^66^ | Serious | Low | Serious | Low | Serious |
| Sidiq et al. (2025)^67^ | Low | Low | Low | Serious | Serious |
| Singh et al. (2024)^68^ | Moderate | Moderate | Low | Moderate | Serious |
| Yang et al. (2025)^69^ | Moderate | Serious | Low | Low | Moderate |

**Supplemental Table 13: Certainty of evidence for cardiovascular mortality.**

| **Systematic review** | **Effect measure** | **N of participants** | **Certainty of evidence** | **Comments** |
| --- | --- | --- | --- | --- |
| **Intervention / comparison** | **(95% CI)** | **(studies)** | **(GRADE)** |  |
| **Affas et al. (2025)**^52^ | RR 0·62 | 14,784 | Very Low | Moderate risk of bias. Serious inconsistency and imprecision (the confidence interval includes the possibility of no effect). Publication bias was not assessed. |
| Use of beta-blockers after hospital discharge / non-use of beta blockers | (0·28 - 1·34) | (4 observational and 2 RCTs) |  |  |
| **Alnemer et al. (2025)**^53^ | HR 0·76 | 46,346 | Low | Moderate risk of bias and inconsistency. |
| Use of beta-blockers / non-use of beta blockers | (0·59 - 0·99) | (7 observational and 2 RCTs) |  |  |
| **Chen et al. (2025)**^54^ | Only provides subgroup analyses results | 41,898 | Very Low | Moderate risk of bias and imprecision. Serious inconsistency and publication bias not reported. |
| Use of beta-blockers / non-use of beta blockers |  | (5 observational and 2 RCTs) |  |  |
| **Gomes et al. (2025)**^57^ | HR 0·83 | 68,981 | Low | Moderate risk of bias and serious inconsistency and imprecision. Publication bias not assessed. |
| Use of beta-blockers / non-use of beta blockers or placebo | (0·57 - 1·20) | (6 observational and 1 RCT) |  |  |
| **(1) Hu et al. (2022)**^58^ | OR 0·63 | 9,477 | Moderate | Moderate risk of bias (Although only PSM studies were included to minimise confounding, a quality assessment of each primary study was not done) |
| Use of beta-blockers after hospital discharge / non-use of beta blockers | (0·44 to 0·91) | (4 observational and 1 RCT) |  |  |
| **(2) Hu et al. (2022)**^59^ | OR 0·69 | 48,264 | Low | Moderate risk of bias and serious inconsistency as most included studies are observational. Serious imprecision (wide confidence intervals that include the possibility of no effect). |
| Use of beta-blockers / non-use of beta blockers | (0·47 to 1·03) | (9 observational and 1 RCT) |  |  |
| **Kristensen et al. (2025)**^61^ | HR 1·26 | 15,360 | Moderate | Serious imprecision (wide confidence intervals that include the possibility of no effect) and publication bias not assessed. |
| Use of beta-blockers / non-use of beta blockers | (0·94 - 1·7) | (5 RCTs) |  |  |
| **Liang et al. (2022)**^62^ | HR 0·62 | 35,392 | Moderate | Moderate risk of bias and serious inconsistency as al included studies are observational. |
| Use of beta-blockers / non-use of beta blockers | (0·49 to 0·78) | (11 observational) |  |  |
| **Rossello et al. (2025)**^64^ | HR 0·55 | 1,855 | Moderate | Serious imprecision (wide confidence intervals that include the possibility of no effect) and publication bias not assessed. |
| Use of beta-blockers / non-use of beta blockers | (0·28 - 1·06) | (4 RCTs) |  |  |
| **Sabina et al. (2025)**^24^ | RR 1·22 | 9,512 | Low | Serious imprecision (wide confidence interval that includes the possibility of no effect). Publication bias was not assessed. |
| Treated with beta-blockers / not treated with beta-blockers | (0·87 to 1·72) | (3 RCT) |  |  |
| **Safi et al. (2019)**^65^ | RR 0·9 | 22,457 | Moderate | Serious risk of bias (as all but one of the included primary studies were rated high risk), and indirectness (the trials predominantly enrolled younger patients and were conducted in the pre-reperfusion era, limiting their relevance to contemporary clinical practice) and publication bias. |
| Use of any type of beta-blocker (including intravenous and oral therapy) / placebo, no intervention or co-intervention | (0·83 to 0·98) * | (14 RCT) |  |  |
| **Safi et al. (2021)**^66^ | RR 0·73 | 21,763 | Very Low | Serious risk of bias (as all but one of the included primary studies were rated high risk), indirectness (the trials predominantly enrolled younger patients and were conducted in the pre-reperfusion era, limiting their relevance to contemporary clinical practice) and publication bias. Moderate inconsistency. |
| Use of any type of beta-blocker (including intravenous and oral therapy) / placebo, no intervention or co-intervention | (0·61 to 0·88) * | (19 RCT) |  |  |
| **Singh et al. (2024)**^68^ | OR 0·77 | 18,691 | Very Low | Moderate risk of bias. Serious inconsistency and imprecision (the confidence interval includes the possibility of no effect). Publication bias was not assessed. |
| Use of beta-blockers / non-use of beta blockers | (0·45 to 1·33) | (3 observational and 2 RCT) |  |  |
| **Yang et al. (2025)**^69^ | OR 0·79 | 144,566 | Low | Moderate risk of bias and publication bias. Serious inconsistency. |
| Use of beta-blockers / non-use of beta blockers | (0·70 - 0·89) | (30 observational and 4 RCTs) |  |  |

* The confidence interval reported was 98%

N° = Number; CI = Confidence Interval; GRADE = Grading of Recommendations Assessment, Development, and Evaluation; HR = Hazard Ratio; RCTs = Randomized Controlled Trials; RR = Risk Ratio; OR = Odds Ratio; PSM = Propensity Score Matching.

**Supplemental Table 14: GRADE assessment details for cardiovascular mortality.**

| **Systematic review** | **Risk of bias** | **Inconsistency** | **Indirectness** | **Imprecision** | **Publication bias** |
| --- | --- | --- | --- | --- | --- |
| Affas et al. (2025)^52^ | Moderate | Serious | Low | Serious | Serious |
| Alnemer et al. (2025)^53^ | Moderate | Moderate | Low | Low | Low |
| Chen et al. (2025)^54^ | Moderate | Moderate | Low | Serious | Serious |
| Gomes et al. (2025)^57^ | Moderate | Serious | Low | Serious | Serious |
| (1) Hu et al. (2022)^58^ | Moderate | Low | Low | Low | Low |
| (2) Hu et al. (2022)^59^ | Moderate | Serious | Low | Serious | Low |
| Kristensen et al. (2025)^61^ | Low | Low | Low | Serious | Serious |
| Liang et al. (2022)^62^ | Moderate | Serious | Low | Low | Low |
| Rossello et al. (2025)^64^ | Low | Low | Low | Serious | Serious |
| Sabina et al. (2025)^24^ | Low | Low | Low | Serious | Serious |
| Safi et al. (2019)^65^ | Serious | Low | Serious | Low | Low |
| Safi et al. (2021)^66^ | Serious | Moderate | Serious | Low | Serious |
| Singh et al. (2024)^68^ | Moderate | Serious | Low | Serious | Serious |
| Yang et al. (2025)^69^ | Moderate | Serious | Low | Low | Moderate |

**Supplemental Table 15: Certainty of evidence for recurrent MI.**

| **Systematic review** | **Effect measure** | **N of participants** | **Certainty of evidence** | **Comments** |
| --- | --- | --- | --- | --- |
| **Intervention / comparison** | **(95% CI)** | **(studies)** | **(GRADE)** |  |
| **Affas et al. (2025)**^52^ | RR 0·87 | 14,784 | Low | Moderate risk of bias. Serious imprecision (the confidence interval includes the possibility of no effect). Publication bias was not assessed. |
| Use of beta-blockers after hospital discharge / non-use of beta blockers | (0·71 - 1·07) | (4 observational and 2 RCTs) |  |  |
| **Alnemer et al. (2025)**^53^ | HR 0·98 | Nos specified | Moderate | Moderate risk of bias and imprecision. Publication bias was not assessed. |
| Use of beta-blockers / non-use of beta blockers | (0·82 - 1·17) |  |  |  |
| **Gomes et al. (2025)**^57^ | HR 1 | 77,161 | Low | Moderate risk of bias and serious imprecision· Publication bias not assessed. |
| Use of beta-blockers / non-use of beta blockers or placebo | (0·92 - 1·09) | (5 observational and 1 RCT) |  |  |
| **(1) Hu et al. (2022)**^58^ | OR 0·73 | 30,069 | Moderate | Moderate risk of bias (Although only PSM studies were included to minimise confounding, a quality assessment of each primary study was not done) |
| Use of beta-blockers after hospital discharge/ non-use of beta blockers | (0·62 to 0·86) | (4 observational and 1 RCT) |  |  |
| **(2) Hu et al. (2022)**^59^ | OR 1·11 | 45,194 | Moderate | Moderate risk of bias as most included studies are observational. Serious imprecision (wide confidence intervals that include the possibility of no effect). |
| Use of beta-blockers / non-use of beta blockers | (0·99 to 1·25) | (6 observational and 1 RCT) |  |  |
| **Kristensen et al. (2025)**^61^ | HR 0·89 | 17,801 | Moderate | Moderate imprecision (narrow confidence intervals that include the possibility of no effect) and publication bias not assessed. |
| Use of beta-blockers / non-use of beta blockers | (0·77 - 1·03) | (5 RCTs) |  |  |
| **Liang et al. (2022)**^62^ | HR 0·93 | 40,786 | Low | Moderate risk of bias and inconsistency as all included studies are observational. Serious imprecision (wide confidence intervals that include the possibility of no effect). |
| Use of beta-blockers / non-use of beta blockers | (0·78 to 1·11) | (9 observational) |  |  |
| **Rossello et al. (2025)**^64^ | HR 0·77 | 1,855 | Moderate | Serious imprecision (wide confidence intervals that include the possibility of no effect) and publication bias not assessed. |
| Use of beta-blockers / non-use of beta blockers | (0·50 - 1·18) | (4 RCTs) |  |  |
| **Sabina et al. (2025)**^24^ | RR 0·97 | 9,512 | Low | Serious imprecision (wide confidence interval that includes the possibility of no effect). Publication bias was not assessed. |
| Treated with beta-blockers / not treated with beta-blockers | (0·78 to 1·19) | (3 RCTs) |  |  |
| **Safi et al. (2019)**^65^ | RR 0·89 | 6,825 | Low | Serious risk of bias (as all but one of the included primary studies were rated high risk), indirectness (the trials predominantly enrolled younger patients and were conducted in the pre-reperfusion era, limiting their relevance to contemporary clinical practice) and imprecision (the confidence interval includes the possibility of no effect) |
| Use of any type of beta-blocker (including intravenous and oral therapy) / placebo, no intervention or co-intervention | (0·75 to 1·08) * | (14 RCTs) |  |  |
| **Safi et al. (2021)**^66^ | RR 0·76 | 19,606 | Moderate | Serious risk of bias (as all but one of the included primary studies were rated high risk), indirectness (the trials predominantly enrolled younger patients and were conducted in the pre-reperfusion era, limiting their relevance to contemporary clinical practice). |
| Use of any type of beta-blocker (including intravenous and oral therapy) / placebo, no intervention or co-intervention | (0·67 to 0·86) * | (19 RCTs) | ` |  |
| **Sidiq et al. (2025)**^67^ | RR 0·88 | 19,826 | Moderate | Serious imprecision (wide confidence intervals that include the possibility of no effect) and publication bias not assessed. |
| Use of beta-blockers / non-use of beta blockers | (0·75 - 1·04) | (4 RCTs) |  |  |
| **Singh et al. (2024)**^68^ | OR 1·00 | 18,691 | Low | Moderate risk of bias. Serious imprecision (the confidence interval includes the possibility of no effect). Publication bias was not assessed. |
| Use of beta-blockers / non-use of beta blockers | (0·82 to 1·22) | (3 observational and 2 RCTs) |  |  |

* The confidence interval reported was 98%

N° = Number; CI = Confidence Interval; GRADE = Grading of Recommendations Assessment, Development, and Evaluation; HR = Hazard Ratio; RCTs = Randomized Controlled Trials; RR = Risk Ratio; OR = Odds Ratio; PSM = Propensity Score Matching.

**Supplemental Table 16: GRADE assessment details for recurrent MI.**

| **Systematic review** | **Risk of bias** | **Inconsistency** | **Indirectness** | **Imprecision** | **Publication bias** |
| --- | --- | --- | --- | --- | --- |
| Affas et al. (2025)^52^ | Moderate | Low | Low | Serious | Serious |
| Alnemer et al. (2025)^53^ | Moderate | Low | Low | Moderate | Serious |
| Gomes et al. (2025)^57^ | Moderate | Low | Low | Serious | Serious |
| (1) Hu et al. (2022)^58^ | Moderate | Low | Low | Low | Low |
| (2) Hu et al. (2022)^59^ | Moderate | Low | Low | Serious | Low |
| Kristensen et al. (2025)^61^ | Low | Low | Low | Moderate | Serious |
| Liang et al. (2022)^62^ | Moderate | Moderate | Low | Serious | Low |
| Rossello et al. (2025)^64^ | Low | Low | Low | Serious | Serious |
| Sabina et al. (2025)^24^ | Low | Low | Low | Serious | Serious |
| Safi et al. (2019)^65^ | Serious | Low | Serious | Serious | Low |
| Safi et al. (2021)^66^ | Serious | Low | Serious | Low | Low |
| Sidiq et al. (2025)^67^ | Low | Low | Low | Serious | Serious |
| Singh et al. (2024)^68^ | Moderate | Low | Low | Serious | Serious |

**Supplemental Table 17: Mapping of primary studies included in the systematic reviews.**

| **Study Type** | **Primary studies** | **Affas et al. (2025)**^52^ | **Alnemer et al. (2025)**^53^ | **Chen et al. (2025)**^54^ | **Chi et al. (2024)**^55^ | **Dahl Aarvik et al. (2019)**^56^ | **Gomes et al. (2025)**^57^ | **(1) Hu et al. (2022)**^58^ | **(2) Hu et al. (2022)**^59^ | **Kim et al. (2022)**^60^ | **Kristensen et al. (2025)**^61^ | **Liang et al. (2022)**^62^ | **Maqsood et al. (2021)**^63^ | **Rossello et al. (2025)**^64^ | **Sabina et al. (2025)**^24^ | **Safi et al. (2019)**^65^ | **Safi et al. (2021)**^66^ | **Sidiq et al. (2025)**^67^ | **Singh et al. (2024)**^68^ | **Yang et al. (2025)**^69^ |
| --- | --- | --- | --- | --- | --- | --- | --- | --- | --- | --- | --- | --- | --- | --- | --- | --- | --- | --- | --- | --- |
| **RCTs** | | | | | | | | | | | | | | | | | | | | |
| 1 | Amano et al. (2023)^77^ |  |  |  |  |  |  |  |  |  |  |  |  |  |  |  |  |  | x | x |
| 2 | Bangalore et al. (2014)^81^ |  |  |  |  | x |  |  |  |  |  | x |  |  |  |  |  |  |  | x |
| 3 | Dargie et al. (2001)^70^ |  |  |  |  |  |  |  |  |  |  |  |  |  |  | x |  |  |  | x |
| 4 | Hioki et al. (2016)^90^ |  |  |  |  | x |  |  | x |  |  | x |  |  |  |  |  |  |  |  |
| 5 | Ibanez et al. (2025)^71^ |  |  |  |  |  |  |  |  |  | x |  |  | x |  |  |  | x |  |  |
| 6 | McMurray et al. (2005)^103^ |  |  |  |  |  |  |  |  |  |  |  |  |  |  |  |  |  |  | x |
| 7 | Munkhaugen et al. (2025)^72^ |  |  |  |  |  |  |  |  |  | x |  |  | x |  |  |  | x |  |  |
| 8 | Silvain et al. (2024)^73^ |  | x |  | x |  | x |  |  |  |  |  |  |  | x |  |  |  |  |  |
| 9 | Watanabe et al. (2018)^74^ | x | x | x | x |  |  | x | x |  | x |  | x | x | x | x | x | x |  | x |
| 10 | Yndigegn et al. (2024)^75^ | x | x | x | x |  | x |  |  |  | x |  |  |  | x |  |  | x | x | x |
| **Observational without PSM** | | | | | | | | | | | | | | | | | | | | |
| 1 | Andell et al. (2015)^78^ |  |  |  |  |  |  |  |  |  |  | x |  |  |  |  |  |  |  | x |
| 2 | Arós et al. (2006)^79^ |  |  |  |  |  |  |  |  |  |  | x |  |  |  |  |  |  |  |  |
| 3 | Bao et al. (2013)^82^ |  | x | x |  | x |  |  | x |  |  | x | x |  |  |  |  |  |  | x |
| 4 | Chen et al. (2020)^83^ |  |  |  |  |  |  |  | x |  |  |  |  |  |  |  |  |  |  |  |
| 5 | De Luca et al. (2005)^87^ |  |  |  |  |  |  |  |  |  |  |  | x |  |  |  |  |  |  |  |
| 6 | Dondo et al. (2017)^88^ |  |  |  | x | x |  |  | x | x |  | x |  |  |  |  |  |  |  |  |
| 7 | El Nasasra et al. (2020)^89^ |  |  | x | x |  | x |  | x | x |  |  |  |  |  |  |  |  |  |  |
| 8 | Holt et al. (2021)^91^ |  |  | x |  |  |  |  | x | x |  |  |  |  |  |  |  |  |  | x |
| 9 | Hwang et al. (2019)^92^ |  |  |  |  |  |  |  |  |  |  | x |  |  |  |  |  |  |  | x |
| 10 | Ishak et al. (2023)^93^ |  |  |  | x |  | x |  |  |  |  |  |  |  |  |  |  |  |  | x |
| 11 | Ishikawa et al. (2000)^94^ |  |  |  |  |  |  |  |  |  |  | x |  |  |  |  |  |  |  |  |
| 12 | Jackevicius et al. (2020)^95^ |  |  |  | x |  |  |  |  |  |  |  |  |  |  |  |  |  |  | x |
| 13 | Lee et al. (2015)^100^ | x | x | x | x | x |  |  | x |  |  | x | x |  |  |  |  |  | x | x |
| 14 | Lee et al. (2016)^101^ |  |  |  |  | x |  |  |  |  |  | x |  |  |  |  |  |  |  |  |
| 15 | Okuno et al. (2019)^105^ |  |  |  |  |  |  |  |  |  |  |  |  |  |  |  |  |  |  | x |
| 16 | Ozasa et al. (2010)^106^ |  | x | x |  | x |  |  | x |  |  | x | x |  |  |  |  |  |  | x |
| 17 | Park et al. (2019)^108^ |  |  |  | x |  |  |  |  |  |  |  |  |  |  |  |  |  |  | x |
| 18 | Peck et al. (2020)^110^ |  |  |  | x |  |  |  |  |  |  |  |  |  |  |  |  |  |  |  |
| 19 | Rochon et al. (2000)^113^ |  |  |  |  |  |  |  |  |  |  | x |  |  |  |  |  |  |  | x |
| 20 | Sakagami et al. (2023)^114^ |  |  | x |  |  |  |  |  |  |  |  |  |  |  |  |  |  |  |  |
| 21 | Shavadia et al. (2019)^115^ |  |  |  |  |  |  |  |  |  |  | x |  |  |  |  |  |  |  | x |
| 22 | Siu et al. (2010)^116^ |  |  | x | x |  | x |  |  |  |  | x |  |  |  |  |  |  |  | x |
| 23 | Song et al. (2021)^117^ |  |  |  |  |  | x |  |  |  |  |  |  |  |  |  |  |  |  |  |
| 24 | Yamada et al. (2006)^120^ |  |  |  |  | x |  |  |  |  |  | x |  |  |  |  |  |  |  | x |
| **Observational with PSM** | | | | | | | | | | | | | | | | | | | | |
| 1 | Bangalore et al. (2012)^80^ |  |  |  |  | x |  |  |  |  |  | x |  |  |  |  |  |  |  | x |
| 2 | D'Ascenzo et al. (2018)^86^ | x |  |  |  |  |  |  |  |  |  |  | x |  |  |  |  |  |  |  |
| 3 | Joo et al. (2022)^98^ |  |  | x | x |  |  |  |  |  |  |  |  |  |  |  |  |  |  |  |
| 4 | Konishi et al. (2016)^99^ |  | x | x | x | x |  | x | x |  |  | x | x |  |  |  |  |  |  | x |
| 5 | Lee et al. (2020)^102^ |  |  |  |  |  |  | x |  |  |  |  |  |  |  |  |  |  |  |  |
| 6 | Raposeiras-Roubin et al. (2015)^112^ |  | x |  | x | x | x |  |  |  |  | x |  |  |  |  |  |  | x | x |
| 7 | Won et al. (2020)^119^ |  |  |  |  |  |  | x |  |  |  |  |  |  |  |  |  |  |  |  |
| **Observational with and without PSM** | | | | | | | | | | | | | | | | | | | | |
| 1 | Al-Bawardy et al. (2024)^76^ |  | x |  |  |  |  |  |  |  |  |  |  |  |  |  |  |  |  |  |
| 2 | Chen et al. (2021)^84^ |  |  |  |  |  | x | x | x |  |  |  |  |  |  |  |  |  |  |  |
| 3 | Choo et al. (2014)^85^ |  | x | x | x | x | x | x | x |  |  | x | x |  |  |  |  |  | x | x |
| 4 | Jeong et al. (2024)^96^ |  |  | x |  |  |  |  |  |  |  |  |  |  |  |  |  |  |  |  |
| 5 | Joo et al. (2021)^97^ |  | x | x | x |  | x |  |  |  |  |  |  |  |  |  |  |  | x |  |
| 6 | Nakatani et al. (2013)^104^ |  |  | x | x | x |  | x |  |  |  | x | x |  |  |  |  |  |  | x |
| 7 | Park et al. (2018)^107^ |  |  |  | x |  |  |  |  |  |  |  |  |  |  |  |  |  |  | x |
| 8 | Park et al. (2021)^109^ |  |  |  | x |  |  |  |  |  |  |  |  |  |  |  |  |  |  |  |
| 9 | Puymirat et al. (2016)^111^ |  |  | x | x | x |  | x | x | x |  | x |  |  |  |  |  |  |  |  |
| 10 | Wen et al. (2022)^118^ |  | x | x | x |  | x |  | x |  |  |  |  |  |  |  |  |  | x |  |
| 11 | Yang et al. (2014)^121^ | x | x |  | x | x |  | x |  |  |  | x | x |  |  |  |  |  |  | x |

RCTs = Randomised Controlled Trials; PSM = Propensity Score Matching.

**Supplemental Figure 6: Heatmap of pairwise overlap of primary studies between included systematic reviews.**

Caption: Pairwise overlap of primary studies across 19 systematic reviews. Each cell shows the number of shared primary studies between two reviews, with darker colours representing greater overlap. A total of 52 unique primary studies were included. The overall Corrected Covered Area (CCA) was 15.1%, indicating high overlap.


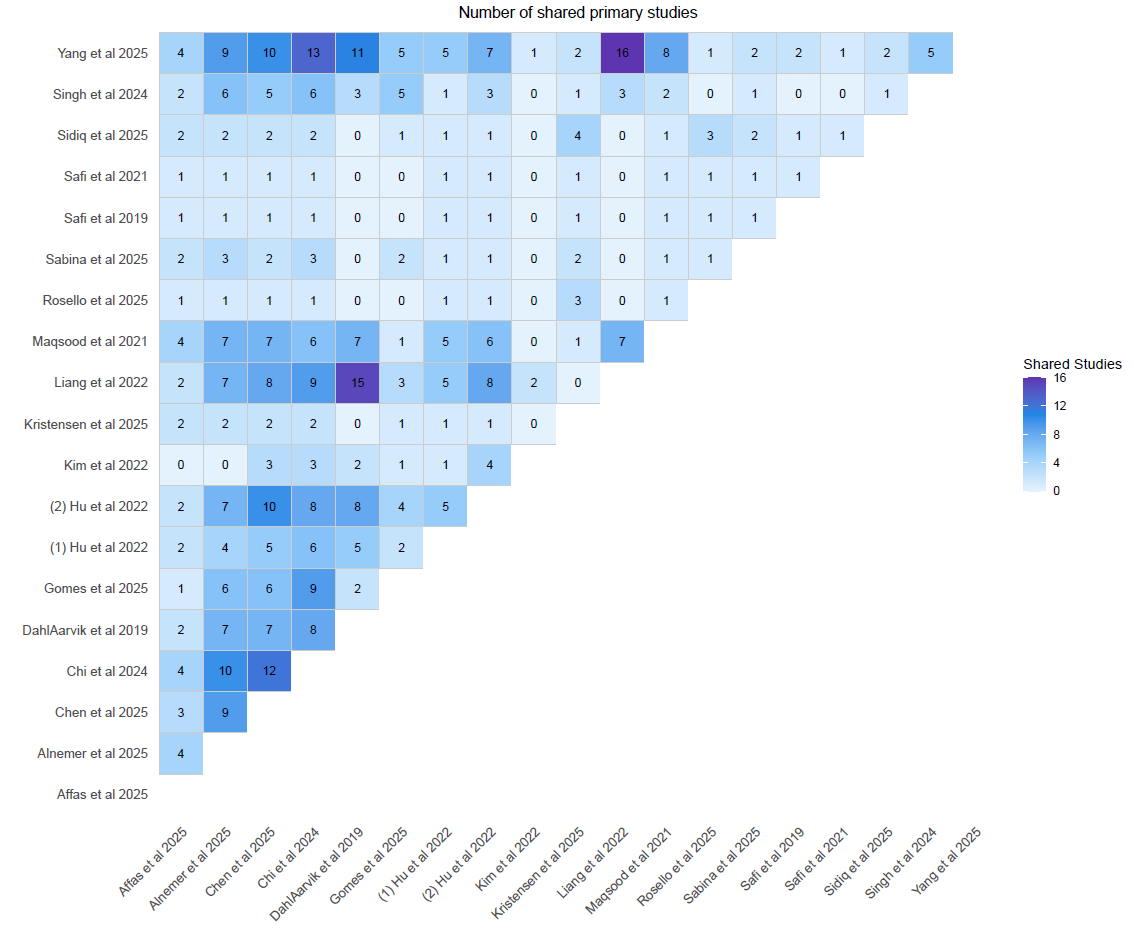


**Supplemental Table 18: Results for sensitivity analyses.**

| **Outcome** | **Study design** | **Number of studies  (original → sensitivity)** | **Original HR  (95% CI)** | **Sensitivity HR (95% CI)** | **Change in HR** | **I² (%) (original → sensitivity)** |
| --- | --- | --- | --- | --- | --- | --- |
| All-cause Mortality * | non-PSM | 32 → 29 | 0·78 (0·69 – 0·88) | 0·83 (0·75 – 0·91) | + 5·9% | 90·9 → 81·9 |
|  | PSM | 18 → 17 | 0·70 (0·59 - 0·82) | 0·71 (0·61 - 0·84) | + 2·2% | 81·4 → 81·4 |
| Cardiovascular mortality† | non-PSM | 18 → 17 | 0·80 (0·65 - 0·98) | 0·78 (0·63 - 0·97) | – 2·3% | 78·9 → 78·7 |
| Recurrent MI† | non-PSM | 14 → 13 | 1·01 (0·94 - 1·08) | 1·00 (0·93 - 1·08) | – 0·5% | 0 → 0 |

PSM = Propensity Score Matching; HR = Hazard Ratio; CI = Confidence Interval; MI = Myocardial Infarction.

* Studies excluded: Rochon et al., De Luca et al., and Al-Bawardy et al.

^†^ Study excluded: Holt et al.

**Supplemental Figure 7: Forest plot for all-cause mortality, cardiovascular mortality and recurrent MI**

Caption: Forest plots summarising pooled odds ratios (ORs) from published systematic reviews and meta-analyses evaluating the association between beta-blocker therapy and clinical outcomes following MI. Beta-blocker therapy was associated with significant reductions in all-cause mortality (OR 0·80, 95% CI 0·74–0·86; I² = 76·3%), cardiovascular mortality (OR 0·81, 95% CI 0·72–0·92; I² = 71·0%), and recurrent MI (OR 0·90, 95% CI 0·84–0·97; I² = 66·6%). Effect estimates were larger and heterogeneity higher than those observed in randomised controlled trials, reflecting the substantial contribution of observational evidence within published reviews.

**
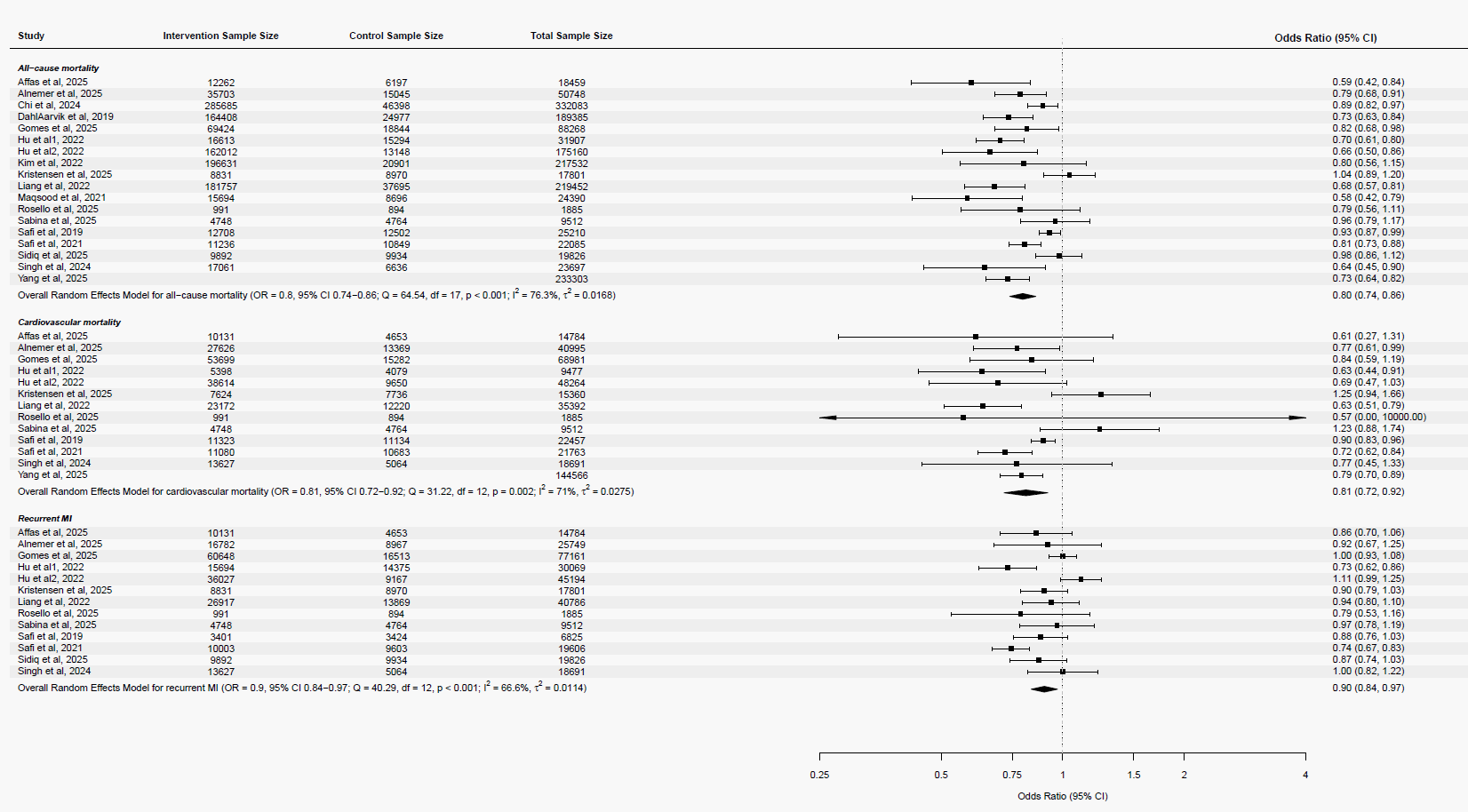
**

**Supplemental Table 19: Results for leave-one-out analyses**

| Outcome | Study Design | Number of studies | Original HR (95% CI) | HR range after removal | Most influential study | Change in HR > 5% | Change in statistical significance |
| --- | --- | --- | --- | --- | --- | --- | --- |
| All-cause mortality | Non-PSM | 32 | 0·78 (0·69–0·88) | 0·77 – 0·81 | Rochon et al. (2000) | No | No |
|  | PSM | 18 | 0·70 (0·59–0·82) | 0·68 – 0·75 | D’Ascenzo et al. (2018) | Yes | No |
|  | RCT | 7 | 0·89 (0·78–1·01) | 0·84 – 0·94 | Dargie et al. (2001) | No | Yes |
| Cardiovascular mortality | Non-PSM | 18 | 0·80 (0·65–0·98) | 0·76 – 0·83 | Yang et al. (2014) | No | Yes |
|  | PSM | 11 | 0·72 (0·57–0·92) | 0·69 – 0·81 | Choo et al. (2014) | Yes | No |
|  | RCT | 5 | 0·94 (0·74–1·19) | 0·86 – 1·07 | Dargie et al. (2001) | Yes | No |
| Recurrent MI | Non-PSM | 14 | 1·01 (0·94–1·08) | 0·99 – 1·02 | Bao et al. (2013) | No | No |
|  | PSM | 9 | 0·83 (0·67–1·03) | 0·77 – 0·87 | Bangalore et al. (2012) | Yes | Yes |
|  | RCT | 6 | 0·80 (0·66–0·97) | 0·75 – 0·84 | Ibañez et al. (2025) | Yes | Yes |

RCTs = Randomised Controlled Trials; PSM = Propensity Score Matching; HR = Hazard Ratio; CI = Confidence Interval; MI = Myocardial Infarction.

**Supplemental Table 20: Summary of Egger’s and Begg’s tests and trim-and-fill analyses for all outcomes.**

| **Outcome** | **Study type** | **Number of studies** | **Egger's z  (p-value)** | **Begg's z (p-value)** | **Original HR  (95% CI)** | **Adjusted HR (95% CI)** | **Studies trimmed** |
| --- | --- | --- | --- | --- | --- | --- | --- |
| All-cause mortality | RCT | 7 | -2·15 (0·08) | -0·52 (0·14) | 0·89 (0·78 - 1·01) | 0·92 (0·82 - 1·04) | 2 |
|  | PSM | 18 | -0·29 (0·78) | -0·18 (0·33) | 0·70 (0·59 - 0·82) | 0·70 (0·59 - 0·82) | 0 |
|  | Non-PSM | 32 | -2·15 (0·04) | -0·03 (0·83) | 0·78 (0·69 - 0·88) | 0·74 (0·65 - 0·84) | 4 |
| Cardiovascular mortality | RCT | 5 | 1·10 (0·35) | 0 (1) | 0·94 (0·74 - 1·19) | 0·93 (0·74 - 1·17) | 1 |
|  | PSM | 11 | -1·58 (0·15) | -0·20 (0·45) | 0·72 (0·57 - 0·92) | 0·80 (0·61 - 1·07) | 2 |
|  | Non-PSM | 18 | 0·34 (0·74) | 0·16 (0·37) | 0·80 (0·65 - 0·98) | 0·75 (0·61 - 0·92) | 3 |
| Recurrent MI | RCT | 6 | -1·09 (0·34) | -0·07 (1) | 0·80 (0·66 - 0·97) | 0·89 (0·73 - 1·08) | 3 |
|  | PSM | 9 | -1·01 (0·34) | 0·0 (1) | 0·83 (0·67 - 1·03) | 0·90 (0·73 - 1·10) | 2 |
|  | Non-PSM | 14 | -0·31 (0·76) | -0·03 (0·92) | 1·01 (0·94 - 1·08) | 1·01 (0·94 - 1·08) | 0 |

HR = Hazard Ratio; CI = Confidence Interval; MI = Myocardial Infarction; RCTs = Randomised Controlled Trials; PSM = Propensity Score Matching·

References

1. Wang N. Conducting Meta-analyses of Proportions in R. *J Behav Data Sci*. 2023;3:64–126.

2. Lin L, Aloe AM. Evaluation of various estimators for standardized mean difference in meta-analysis. *Stat Med*. 2021;40:403–426.

3. Fusar-Poli P, Radua J. Ten simple rules for conducting umbrella reviews. *Evid Based Ment Health*. 2018;21.

4. DerSimonian R, Laird N. Meta-Analysis in Clinical Trials Revisited. *Contemp Clin Trials*. 2015;45:139–145.

5. Arero AG, Vasheghani-Farahani A, Soltani D. Meta-Analysis of the Usefulness of Beta-Blockers to Reduce the Risk of Major Adverse Cardiovascular Events in Patients With Stable Coronary Artery Disease Without Prior Myocardial Infarction or Left Ventricular Dysfunction. *Am J Cardiol*. 2021;158:23–29.

6. Ahmed M, Ahsan A, Shafiq A, et al. Beta-Blockers in Patients With Myocardial Infarction: A Meta-Analysis. *Am J Ther*. 2025;32:e351–e354.

7. Chi KY, Lee PL, Chowdhury I, et al. Mortality effects of beta-blockers on myocardial infarction in patients without reduced ejection fraction or heart failure in the contemporary reperfusion era : a systematic review and meta-analysis. *Eur Heart J*. 2024;45:ehae666.1640.

8. Chi K-Y, Huang C-H, Nanna M, et al. The effect of beta-blockers on mortality in myocardial infarction among patients withoutreduced ejection fraction or heart failure: a systematic review with meta-analysis. *JACC*. 2024;83:1266–1266.

9. Chi K-Y, Lee PL, Chowdhury I, et al. Post-myocardial infarction beta-blockers for patients without reduced ejection fraction: a meta-analysis of contemporary randomized-controlled trials. *JACC*. 2025;85:1937–1937.

10. Clemente M, Lopes L, Navalha D, et al. Long-term effect of beta-blockers after acute myocardial infarction in patients with preserved ejection fraction: A systematic review and meta-analysis. *Circulation*. 2024;150:A4144767.

11. Dar JA, Jacob JR. Beta Blockers in Contemporary Cardiology: Is It Better to Cast Them Out? *Korean Circ J*. 2024;54:165–171.

12. De Filippo O, Russo C, Manai R, et al. Impact of secondary prevention medical therapies on outcomes of patients suffering from Myocardial Infarction with NonObstructive Coronary Artery disease (MINOCA): A meta-analysis. *Int J Cardiol*. 2022;368:1–9.

13. Desta L, Raposeiras-Roubin S, Ibanez B. The Art of Prescribing β-Blockers After Myocardial Infarction. *Circ Cardiovasc Interv*. 2021;14:e010720.

14. Gomez V, GAMBETTA M, Bacca C, Machado M, Pereira LG, Martignoni F. Is it middle child syndrome? the use of beta-blockers in acute mi patients with mildly reduced ejection fraction: a systematic review and meta-analysis of randomized controlled trials and observational studies with subgroup analysis. *JACC*. 2025;85:1667–1667.

15. Harari R, Bangalore S. Beta-blockers after acute myocardial infarction: an old drug in urgent need of new evidence! *Eur Heart J*. 2020;41:3530–3532.

16. Irtaza A, Juniad M, Saqlain M, et al. Efficacy and Safety of Beta Blockers for Patients with Myocardial Infarction in the Percutaneous Coronary Intervention Era: A Systematic Review and Meta-Analysis [Preprint]. *ResearchGate*. 2025. Published onlineMarch 17, 2025. https://doi.org/10.1101/2024.09.18.24313821.

17. Jain H, Odat RM, Durrani T, et al. Clinical outcomes with beta blockers after myocardial infarction: a metaanalysis of randomized controlled trials. *JACC*. 2025;85:1799–1799.

18. Janský P. Treatment with beta blockers in myocardial infarction in the era of primary PCI. *Interv Akutní Kardiologie*. 2019;18:96–98.

19. Johner N, Gencer B, Roffi M. Routine beta-blocker therapy after acute coronary syndromes: The end of an era? *Eur J Clin Invest*. 2024;54:e14309.

20. Lanham D, Ali S, Davis D, Rawle MJ. Beta-Blockers for the Secondary Prevention of Myocardial Infarction in People with Dementia: A Systematic Review. *J Alzheimers Dis JAD*. 2019;71:1105–1114.

21. Martinez-Milla J, Raposeiras-Roubin S, Pascual-Figal DA, Ibanez B. Role of Beta-blockers in Cardiovascular Disease in 2019. *Rev Espanola Cardiol Engl Ed*. 2019;72:844–852.

22. Ottani F, Staszewsky L, Latini R, a nome della Rete Italiana REBOOT (elenco completo nell’Addenda online). Beta-blockers after myocardial infarction with preserved systolic function: useful or redundant? *G Ital Cardiol 2006*. 2022;23:932–937.

23. Rosa CR, Diaz CAV, Natali LD, Borges E, Portilho N. Post-myocardial infarction beta-blockers therapy in patients with preserved ejection fraction and mildly reduced ejection fraction: a systematic review and meta-analysis. *JACC*. 2025;85:712–712.

24. Sabina M, Trube J, Luna G, Bizanti A. Reply to Acerbo et al. Do Beta-Blockers Really Matter in Patients with Myocardial Infarction Without Left Ventricular Systolic Dysfunction? Comment on “Sabina et al. Beta-Blockers in Patients with Myocardial Infarction and Preserved Left Ventricular Ejection: A Systematic Review and Meta-Analysis of Randomized Controlled Trials. J. Clin. Med. 2025, 14, 150.” *J Clin Med*. 2025;14:2249.

25. Vutthikraivit W, Rattanawong P, Klomjit S, et al. Long-term use of oral beta-blocker in patients with acute myocardial infarction who underwent primary coronary intervention: a systematic review and meta-analysis. *JACC*. 2019;73:1396–1396.

26. Zamiri N, Alradaddi H, Adli T, et al. Efficacy of betablockers in patients with acute coronary syndrome: a systematic review and meta analysis of randomized trials. *Eur Heart J*. 2020;41:ehaa946.1741.

27. Zamiri N, Alraddadi H, Adli T, et al. Beta Blockers in patients with Acute Coronary Syndrome: A Systematic Review and Meta-analysis of Randomized Trials in the Reperfurion Era. *Can J Cardiol*. 2020;36:S6–S7.

28. Zeitouni M, Kerneis M, Lattuca B, et al. Do Patients need Lifelong β-Blockers after an Uncomplicated Myocardial Infarction? *Am J Cardiovasc Drugs Drugs Devices Interv*. 2019;19:431–438.

29. Zhang J-G, Dai S-P, Liu H, Xu Z-S. Comparison of carvedilol versus metoprolol in patients with acute myocardial infarction: A protocol for systematic review and meta-analysis. *Medicine (Baltimore)*. 2021;100:e25855.

30. Zhao W, Zhao J, Rong J. Pharmacological Modulation of Cardiac Remodeling after Myocardial Infarction. *Oxid Med Cell Longev*. 2020;2020:8815349.

31. Chen ZM, Pan HC, Chen YP, et al. Early intravenous then oral metoprolol in 45,852 patients with acute myocardial infarction: randomised placebo-controlled trial. *Lancet Lond Engl*. 2005;366:1622–1632.

32. Coiro S, Girerd N, Rossignol P, et al. Association of beta-blocker treatment with mortality following myocardial infarction in patients with chronic obstructive pulmonary disease and heart failure or left ventricular dysfunction: a propensity matched-cohort analysis from the High-Risk Myocardial Infarction Database Initiative. *Eur J Heart Fail*. 2017;19:271–279.

33. Daga MK, Chaudhary M, Sharma B, et al. Effect of esmolol on oxidant status and antioxidant activity in acute myocardial infarction. *J Assoc Physicians India*. 2003;51:677–680.

34. Er F, Dahlem KM, Nia AM, et al. Randomized Control of Sympathetic Drive With Continuous Intravenous Esmolol in Patients With Acute ST-Segment Elevation Myocardial Infarction: The BEtA-Blocker Therapy in Acute Myocardial Infarction (BEAT-AMI) Trial. *JACC Cardiovasc Interv*. 2016;9:231–240.

35. Hanada K, Higuma T, Nishizaki F, et al. Randomized study on the efficacy and safety of landiolol, an ultra-short-acting β1-adrenergic blocker, in patients with acute myocardial infarction undergoing primary percutaneous coronary intervention. *Circ J Off J Jpn Circ Soc*. 2012;76:439–445.

36. Ibanez B, Macaya C, Sánchez-Brunete V, et al. Effect of early metoprolol on infarct size in ST-segment-elevation myocardial infarction patients undergoing primary percutaneous coronary intervention: the Effect of Metoprolol in Cardioprotection During an Acute Myocardial Infarction (METOCARD-CNIC) trial. *Circulation*. 2013;128:1495–1503.

37. Jánosi A, Ghali JK, Herlitz J, et al. Metoprolol CR/XL in postmyocardial infarction patients with chronic heart failure: experiences from MERIT-HF. *Am Heart J*. 2003;146:721–728.

38. Matsumoto S, Henderson AD, Shen L, et al. Beta-blocker use and outcomes in patients with heart failure and mildly reduced and preserved ejection fraction. *Eur J Heart Fail*. 2025;27:124–139.

39. Mitchell RG, Stoddard MF, Ben-Yehuda O, et al. Esmolol in acute ischemic syndromes. *Am Heart J*. 2002;144:E9.

40. Roolvink V, Ibáñez B, Ottervanger JP, et al. Early Intravenous Beta-Blockers in Patients With ST-Segment Elevation Myocardial Infarction Before Primary Percutaneous Coronary Intervention. *J Am Coll Cardiol*. 2016;67:2705–2715.

41. Shirotani M, Yokota R, Kouchi I, et al. Influence of atenolol on coronary artery spasm after acute myocardial infarction in a Japanese population. *Int J Cardiol*. 2010;139:181–186.

42. Tereshchenko SN, Kositsyna IV, Dzhaiani NA, Golubev AV, Kochetov AG. [The use of esmolol in patients with myocardial infarction complicated with acute left ventricular failure]. *Kardiologiia*. 2005;45:19–22.

43. van Veldhuisen DJ, Cohen-Solal A, Böhm M, et al. Beta-blockade with nebivolol in elderly heart failure patients with impaired and preserved left ventricular ejection fraction: Data From SENIORS (Study of Effects of Nebivolol Intervention on Outcomes and Rehospitalization in Seniors With Heart Failure). *J Am Coll Cardiol*. 2009;53:2150–2158.

44. Emery M, López-Sendón J, Steg PG, et al. Patterns of use and potential impact of early beta-blocker therapy in non-ST-elevation myocardial infarction with and without heart failure: the Global Registry of Acute Coronary Events. *Am Heart J*. 2006;152:1015–1021.

45. Goldberger JJ, Bonow RO, Cuffe M, et al. Effect of Beta-Blocker Dose on Survival After Acute Myocardial Infarction. *J Am Coll Cardiol*. 2015;66:1431–1441.

46. Kernis SJ, Harjai KJ, Stone GW, et al. Does beta-blocker therapy improve clinical outcomes of acute myocardial infarction after successful primary angioplasty? *J Am Coll Cardiol*. 2004;43:1773–1779.

47. Kim J, Kang D, Park H, et al. Long-term β-blocker therapy and clinical outcomes after acute myocardial infarction in patients without heart failure: nationwide cohort study. *Eur Heart J*. 2020;41:3521–3529.

48. Konishi M, Haraguchi G, Yoshikawa S, Kimura S, Inagaki H, Isobe M. Additive effects of β-blockers on renin-angiotensin system inhibitors for patients after acute myocardial infarction treated with primary coronary revascularization. *Circ J Off J Jpn Circ Soc*. 2011;75:1982–1991.

49. LaFon DC, Helgeson ES, Lindberg S, et al. β-Blocker Use and Clinical Outcomes in Patients With COPD Following Acute Myocardial Infarction. *JAMA Netw Open*. 2024;7:e247535.

50. Padilla López A, Alós-Almiñana M, Peris JE. Health Outcomes and Primary Adherence to Secondary Prevention Treatment after St-Elevation Myocardial Infarction: a Spanish Cohort Study. *J Cardiovasc Transl Res*. 2021;14:308–316.

51. Thattassery E, Gheorghiade M. Beta blocker therapy after acute myocardial infarction in patients with heart failure and systolic dysfunction. *Heart Fail Rev*. 2004;9:107–113.

52. Affas Z, Patel K, Abuzahrieh OR, et al. Prophylactic Beta-Blocker Therapy in Patients Who Underwent Primary Percutaneous Coronary Intervention for ST-Elevation Myocardial Infarction With Preserved Left Ventricular Ejection Fraction: A Systematic Review and Meta-Analysis. *Cureus*. 2025;17.

53. Alnemer KA. Exploring the Impact of Beta-Blockers Post-Acute Myocardial Infarction in Patients with Preserved Ejection Fraction: A Meta-Analysis. *J Clin Med*. 2025;14:NA-NA.

54. Chen Y, Qiu J, Ai T, Wu Y. The Role of Oral β-Blockers in Patients With Acute Myocardial Infarction with Preserved or Mildly Reduced Left Ventricular Systolic Function: A Systematic Review and Meta-Analysis. *Clin Ther*. 2025;47:788–797.

55. Chi K-Y, Lee P-L, Chowdhury I, et al. Beta-blockers for secondary prevention following myocardial infarction in patients without reduced ejection fraction or heart failure: an updated meta-analysis. *Eur J Prev Cardiol*. 2025;32:633–646.

56. Dahl Aarvik M, Sandven I, Dondo TB, et al. Effect of oral β-blocker treatment on mortality in contemporary post-myocardial infarction patients: a systematic review and meta-analysis. *Eur Heart J — Cardiovasc Pharmacother*. 2019;5:12–20.

57. Gomes RAF, Furtado LCC, Montenegro MV, Filho DCS. Beta-blockers in post-myocardial infarction with preserved ejection fraction: systematic review and meta-analysis. *Cardiovasc Diagn Ther*. 2025;15:398–413.

58. Hu M, Hu S, Gao X, Yang Y. Beta-Blocker Use after Discharge in Patients with Acute Myocardial Infarction in the Contemporary Reperfusion Era. *Med Kaunas Lith*. 2022;58.

59. Hu M-J, Wang X-N, Tan J-S, Yang Y-J. Association of beta-blocker therapy at discharge with clinical outcomes in patients without heart failure or left ventricular systolic dysfunction after acute coronary syndrome: An updated systematic review and meta-analysis. *Arch Cardiovasc Dis*. 2022;115:637–646.

60. Kim Y, Byun S, Kim H-Y, Kim D-B. Long-Term Beta-Blocker Therapy After Myocardial Infarction Without Heart Failure in the Reperfusion Era—Systemic Review and Meta-analysis. *J Cardiovasc Pharmacol*. 2022;79:650.

61. Kristensen AMD, Rossello X, Atar D, et al. Beta-Blockers after Myocardial Infarction with Normal Ejection Fraction. *N Engl J Med*. 2026;394(6):540-550.

62. Liang C, Zhang C, Gan S, Chen X, Tan Z. Long-Term Effect of β-Blocker Use on Clinical Outcomes in Postmyocardial Infarction Patients: A Systematic Review and Meta-Analysis. *Front Cardiovasc Med*. 2022;9.

63. Maqsood MH, Alam M, Atar D, Birnbaum Y. Efficacy of Long-Term Oral Beta-Blocker Therapy in Patients Who Underwent Percutaneous Coronary Intervention for ST-Segment Elevation Myocardial Infarction With Preserved Left Ventricular Ejection Fraction: A Systematic Review and Meta-analysis. *J Cardiovasc Pharmacol*. 2021;77:87–93.

64. Rossello X, Prescott EIB, Kristensen AMD, et al. β blockers after myocardial infarction with mildly reduced ejection fraction: an individual patient data meta-analysis of randomised controlled trials. *The Lancet*. 2025;406:1128–1137.

65. Safi S, Sethi NJ, Nielsen EE, Feinberg J, Jakobsen JC, Gluud C. Beta-blockers for suspected or diagnosed acute myocardial infarction. *Cochrane Database Syst Rev*. 2019;12:CD012484.

66. Safi S, Sethi NJ, Korang SK, et al. Beta‐blockers in patients without heart failure after myocardial infarction. *Cochrane Database Syst Rev*. 2021;2021:CD012565.

67. Sidiq SA, Truly TA, Minhas AMK, et al. Association between Oral Beta Blocker Therapy and long-term Outcomes after Myocardial Infarction in Individuals with Mildly Reduced or Preserved Left Ventricular Function – a Meta-Analysis of Contemporary Randomized Controlled Trials. *Cardiovasc Drugs Ther*. 2025. Published onlineDecember 13, 2025. https://doi.org/10.1007/s10557-025-07826-7.

68. Singh S, Bliden K, Tantry US, Gurbel PA, Kanjwal MY, Lundgren SW. Role of Beta Blockers After Acute Coronary Syndrome With Preserved Ejection Fraction. *Am J Cardiol*. 2025;235:73–75.

69. Yang W, Sun X, Zhang Y, Lu Z, Shu Z, Zhang K. β-blocker and clinical outcomes in patients after myocardial infarction: a systematic review and meta-analysis. *Eur J Clin Pharmacol*. 2025;81:1807–1817.

70. Dargie HJ. Effect of carvedilol on outcome after myocardial infarction in patients with left-ventricular dysfunction: the CAPRICORN randomised trial. *Lancet Lond Engl*. 2001;357:1385–1390.

71. Ibanez B, Latini R, Rossello X, et al. Beta-Blockers after Myocardial Infarction without Reduced Ejection Fraction. *N Engl J Med*. 2025;393(19):1889-1900.

72. Munkhaugen J, Kristensen AMD, Halvorsen S, et al. Beta-Blockers after Myocardial Infarction in Patients without Heart Failure. *N Engl J Med*. 2025;393(19):1901-1911.

73. Silvain J, Cayla G, Ferrari E, et al. Beta-Blocker Interruption or Continuation after Myocardial Infarction. *N Engl J Med*. 2024;391:1277–1286.

74. Watanabe H, Ozasa N, Morimoto T, et al. Long-term use of carvedilol in patients with ST-segment elevation myocardial infarction treated with primary percutaneous coronary intervention. *PloS One*. 2018;13:e0199347.

75. Yndigegn T, Lindahl B, Mars K, et al. Beta-Blockers after Myocardial Infarction and Preserved Ejection Fraction. *N Engl J Med*. 2024;390:1372–1381.

76. Al-Bawardy R, Alqarawi W, Al Suwaidi J, et al. The Effect of Beta-Blocker Post-Myocardial Infarction With Ejection Fraction >40% Pooled Analysis From Seven Arabian Gulf Acute Coronary Syndrome Registries. *Angiology*. 2025;76:476–486.

77. Amano M, Izumi C, Watanabe H, et al. Effects of Long-Term Carvedilol Therapy in Patients With ST-Segment Elevation Myocardial Infarction and Mildly Reduced Left Ventricular Ejection Fraction. *Am J Cardiol*. 2023;199:50–58.

78. Andell P, Erlinge D, Smith JG, et al. β‐Blocker Use and Mortality in COPD Patients After Myocardial Infarction: A Swedish Nationwide Observational Study. *J Am Heart Assoc*. 2015;4:e001611.

79. Arós F, Loma-Osorio A, Vila J, et al. Effect of combined beta-blocker and angiotensin-converting enzyme inhibitor treatment on 1-year survival after acute myocardial infarction: findings of the PRIAMHO-II registry. *Rev Esp Cardiol*. 2006;59:313–320.

80. Bangalore S, Steg G, Deedwania P, et al. β-Blocker use and clinical outcomes in stable outpatients with and without coronary artery disease. *JAMA*. 2012;308:1340–1349.

81. Bangalore S, Bhatt DL, Steg PG, et al. β-blockers and cardiovascular events in patients with and without myocardial infarction: post hoc analysis from the CHARISMA trial. *Circ Cardiovasc Qual Outcomes*. 2014;7:872–881.

82. Bao B, Ozasa N, Morimoto T, et al. β-Blocker therapy and cardiovascular outcomes in patients who have undergone percutaneous coronary intervention after ST-elevation myocardial infarction. *Cardiovasc Interv Ther*. 2013;28:139–147.

83. Chen Y, Tang X-F, Gao R-L, Yang Y-J, Xu B, Yuan J-Q. Association of β-Blocker Therapy at Discharge with Clinical Outcomes after Acute Coronary Syndrome in Patients without Heart Failure. *Cardiovasc Ther*. 2020;2020:4351469.

84. Chen R-Z, Liu C, Zhou P, et al. Prognostic impacts of β-blockers in acute coronary syndrome patients without heart failure treated by percutaneous coronary intervention. *Pharmacol Res*. 2021;169:105614.

85. Choo EH, Chang K, Ahn Y, et al. Benefit of β-blocker treatment for patients with acute myocardial infarction and preserved systolic function after percutaneous coronary intervention. *Heart Br Card Soc*. 2014;100:492–499.

86. D’Ascenzo F, Celentani D, Brustio A, et al. Association of Beta-Blockers with Survival on Patients Presenting with ACS Treated with PCI: A Propensity Score Analysis from the BleeMACS Registry. *Am J Cardiovasc Drugs Drugs Devices Interv*. 2018;18:299–309.

87. De Luca G, de Boer M-J, Ottervanger JP, et al. Impact of beta-blocker therapy at discharge on long-term mortality after primary angioplasty for ST-segment elevation myocardial infarction. *Am J Cardiol*. 2005;96:806–809.

88. Dondo TB, Hall M, West RM, et al. β-Blockers and Mortality After Acute Myocardial Infarction in Patients Without Heart Failure or Ventricular Dysfunction. *J Am Coll Cardiol*. 2017;69:2710–2720.

89. El Nasasra A, Beigel R, Klempfner R, et al. Comparison of Outcomes with or without Beta-Blocker Therapy After Acute Myocardial Infarction in Patients Without Heart Failure or Left Ventricular Systolic Dysfunction (from the Acute Coronary Syndromes Israeli Survey [ACSIS]). *Am J Cardiol*. 2021;143:1–6.

90. Hioki H, Motoki H, Izawa A, et al. Impact of oral beta-blocker therapy on mortality after primary percutaneous coronary intervention for Killip class 1 myocardial infarction. *Heart Vessels*. 2016;31:687–693.

91. Holt A, Blanche P, Zareini B, et al. Effect of long-term beta-blocker treatment following myocardial infarction among stable, optimally treated patients without heart failure in the reperfusion era: a Danish, nationwide cohort study. *Eur Heart J*. 2021;42:907–914.

92. Hwang D, Lee JM, Kim HK, et al. Prognostic Impact of β-Blocker Dose After Acute Myocardial Infarction. *Circ J Off J Jpn Circ Soc*. 2019;83:410–417.

93. Ishak D, Aktaa S, Lindhagen L, et al. Association of beta-blockers beyond 1 year after myocardial infarction and cardiovascular outcomes. *Heart Br Card Soc*. 2023;109:1159–1165.

94. Ishikawa K, Miyataka M, Kanamasa K, et al. beta-blockers reduce the incidence of cardiac events in post-myocardial infarction patients. *Jpn Heart J*. 2000;41:279–294.

95. Jackevicius CA, Krumholz HM, Ross JS, et al. Clinical Outcomes With Beta-Blocker Use in Patients With Recent History of Myocardial Infarction. *Can J Cardiol*. 2020;36:1633–1640.

96. Jeong J-C, Park J-I, Kim B-J, et al. Beta-blockers after percutaneous coronary intervention for acute myocardial infarction and non-reduced left ventricular ejection fraction. *Front Cardiovasc Med*. 2024;11:1447952.

97. Joo S-J, Kim S-Y, Choi J-H, et al. Effect of beta-blocker therapy in patients with or without left ventricular systolic dysfunction after acute myocardial infarction. *Eur Heart J Cardiovasc Pharmacother*. 2021;7:475–482.

98. Joo S-J, Kim S-Y, Lee J-G, et al. Association of the medical therapy with beta-blockers or inhibitors of renin-angiotensin system with clinical outcomes in patients with mildly reduced left ventricular ejection fraction after acute myocardial infarction. *Medicine (Baltimore)*. 2022;101:e30846.

99. Konishi H, Miyauchi K, Kasai T, et al. Long-term effect of β-blocker in ST-segment elevation myocardial infarction in patients with preserved left ventricular systolic function: a propensity analysis. *Heart Vessels*. 2016;31:441–448.

100. Lee Y-H, Park J-S, Tahk S-J, et al. β-Blocker Therapy in the Era of Primary Percutaneous Intervention for ST Elevation Myocardial Infarction. *Cardiology*. 2015;132:91–100.

101. Lee PH, Park G-M, Kim Y-H, et al. Effect of Beta Blockers and Renin-Angiotensin System Inhibitors on Survival in Patients With Acute Myocardial Infarction Undergoing Percutaneous Coronary Intervention. *Medicine (Baltimore)*. 2016;95:e2971.

102. Lee PH, Park G-M, Han S, et al. Beta-blockers provide a differential survival benefit in patients with coronary artery disease undergoing contemporary post-percutaneous coronary intervention management. *Sci Rep*. 2020;10:22121.

103. McMurray J, Køber L, Robertson M, et al. Antiarrhythmic effect of carvedilol after acute myocardial infarction: Results of the Carvedilol Post-Infarct Survival Control in Left Ventricular Dysfunction (CAPRICORN) trial. *J Am Coll Cardiol*. 2005;45:525–530.

104. Nakatani D, Sakata Y, Suna S, et al. Impact of beta blockade therapy on long-term mortality after ST-segment elevation acute myocardial infarction in the percutaneous coronary intervention era. *Am J Cardiol*. 2013;111:457–464.

105. Okuno T, Aoki J, Tanabe K, et al. Admission Heart Rate Is a Determinant of Effectiveness of Beta-Blockers in Acute Myocardial Infarction Patients. *Circ J*. 2019;83:1054–1063.

106. Ozasa N, Kimura T, Morimoto T, et al. Lack of effect of oral beta-blocker therapy at discharge on long-term clinical outcomes of ST-segment elevation acute myocardial infarction after primary percutaneous coronary intervention. *Am J Cardiol*. 2010;106:1225–1233.

107. Park JJ, Kim S-H, Kang S-H, et al. Effect of β-Blockers Beyond 3 Years After Acute Myocardial Infarction. *J Am Heart Assoc*. 2018;7:e007567.

108. Park JJ, Kim S-H, Kang S-H, et al. Differential Effect of β-Blockers According to Heart Rate in Acute Myocardial Infarction Without Heart Failure or Left Ventricular Systolic Dysfunction: A Cohort Study. *Mayo Clin Proc*. 2019;94:2476–2487.

109. Park CS, Yang H-M, Ki Y-J, et al. Left Ventricular Ejection Fraction 1 Year After Acute Myocardial Infarction Identifies the Benefits of the Long-Term Use of β-Blockers: Analysis of Data From the KAMIR-NIH Registry. *Circ Cardiovasc Interv*. 2021;14:e010159.

110. Peck KY, Andrianopoulos N, Dinh D, et al. Role of beta blockers following percutaneous coronary intervention for acute coronary syndrome. *Heart Br Card Soc*. 2021;107:728–733.

111. Puymirat E, Riant E, Aissaoui N, et al. β blockers and mortality after myocardial infarction in patients without heart failure: multicentre prospective cohort study. *BMJ*. 2016;354:i4801.

112. Raposeiras-Roubín S, Abu-Assi E, Redondo-Diéguez A, et al. Prognostic Benefit of Beta-blockers After Acute Coronary Syndrome With Preserved Systolic Function. Still Relevant Today? *Rev Espanola Cardiol Engl Ed*. 2015;68:585–591.

113. Rochon PA, Tu JV, Anderson GM, et al. Rate of heart failure and 1-year survival for older people receiving low-dose β-blocker therapy after myocardial infarction. *The Lancet*. 2000;356:639–644.

114. Sakagami A, Soeda T, Saito Y, et al. Clinical impact of beta-blockers at discharge on long-term clinical outcomes in patients with non-reduced ejection fraction after acute myocardial infarction. *J Cardiol*. 2023;81:83–90.

115. Shavadia JS, Holmes DN, Thomas L, et al. Comparative Effectiveness of β-Blocker Use Beyond 3 Years After Myocardial Infarction and Long-Term Outcomes Among Elderly Patients. *Circ Cardiovasc Qual Outcomes*. 2019;12:e005103.

116. Siu C-W, Pong V, Jim M-H, et al. Beta-blocker in post-myocardial infarct survivors with preserved left ventricular systolic function. *Pacing Clin Electrophysiol PACE*. 2010;33:675–680.

117. Song PS, Kim M, Seong S-W, et al. Heart failure with mid-range ejection fraction and the effect of β-blockers after acute myocardial infarction. *Heart Vessels*. 2021;36:1848–1855.

118. Wen X-S, Luo R, Liu J, et al. Short-term/long-term prognosis with or without beta-blockers in patients without heart failure and with preserved ejection fraction after acute myocardial infarction: a multicenter retrospective cohort study. *BMC Cardiovasc Disord*. 2022;22:193.

119. Won H, Suh Y, Kim GS, Ko YG, Hong MK. Clinical Impact of Beta Blockers in Patients with Myocardial Infarction from the Korean National Health Insurance Database. *Korean Circ J*. 2020;50:499–508.

120. Yamada K, Tsuji H, Tokunaga S, et al. Effect of beta-blockers on the mortality of Japanese patients with myocardial infarction. *Int J Cardiol*. 2006;108:309–313.

121. Yang JH, Hahn J-Y, Song YB, et al. Association of beta-blocker therapy at discharge with clinical outcomes in patients with ST-segment elevation myocardial infarction undergoing primary percutaneous coronary intervention. *JACC Cardiovasc Interv*. 2014;7:592–601.
